# An Ongoing Search for Multitarget Ligands as Potential Agents for Diabetes Mellitus and Its Long-Term Complications: New Insights into (5-Arylidene-4-oxothiazolidin-3-yl)alkanoic Acid Derivatives

**DOI:** 10.3390/ph18121863

**Published:** 2025-12-05

**Authors:** Rosanna Maccari, Rosaria Ottanà, Valerij Talagayev, Roberta Moschini, Francesco Balestri, Francesca Felice, Francesca Iannuccilli, Gemma Sardelli, Rebecca Sodano, Gerhard Wolber, Paolo Paoli, Antonella Del Corso

**Affiliations:** 1 Department of Chemical, Biological, Pharmaceutical and Environmental Sciences, University of Messina, Viale F. Stagno d’Alcontres 31, 98166 Messina, Italy; rottana@unime.it; 2 Molecular Design Group, Institute of Pharmacy, Freie Universität Berlin, Königin-Luisestr. 2 + 4, 14195 Berlin, Germany; v.talagayev@fu-berlin.de (V.T.); gerhard.wolber@fu-berlin.de (G.W.); 3 Biochemistry Unit, Department of Biology, University of Pisa, via S. Zeno, 51, 56123 Pisa, Italy; roberta.moschini@unipi.it (R.M.); francesca.felice@unipi.it (F.F.); gemma.sardelli@phd.unipi.it (G.S.); antonella.delcorso@unipi.it (A.D.C.); 4 Department of Scienze Biomediche Sperimentali e Cliniche, Sezione di Scienze Biochimiche, University of Firenze, Viale Morgagni 50, 50134 Firenze, Italy; f.iannuccilli@student.unisi.it (F.I.); rebecca.sodano@unifi.it (R.S.); paolo.paoli@unifi.it (P.P.)

**Keywords:** diabetes mellitus, enzyme inhibition, multitarget ligands, in silico studies, structure-activity relationships

## Abstract

**Background**: Diabetes mellitus is a multifactorial disease characterized by complex metabolic dysfunctions and chronic complications induced by hyperglycaemia. The design of multitarget ligands, capable of simultaneously controlling different pathogenic processes, was proposed as a promising approach to identify novel antidiabetic drugs endowed with improved efficacy. **Methods**: (5-Arylidene-4-oxothiazolidin-3-yl)alkanoic acid derivatives **1a**–**g** and **2a**–**g** were synthesized as potential multitarget antidiabetic agents. They were tested in vitro as inhibitors of both human recombinant AKR1B1 and PTP1B, and kinetic studies and molecular docking simulations for both enzymes were performed. Their effects on cellular glucose uptake, insulin signalling, and mitochondrial potential were assayed in cultures of murine C2C12 myocytes. A lipid accumulation assay was performed in HepG2 liver cells. The effects on high glucose-induced sorbitol accumulation were evaluated in lens HLE and retinal MIO-M1 cells. **Results**: All compounds displayed excellent AKR1B1 inhibitory activity (IC_50_ 0.03–0.46 μM **1a**–**g**; IC_50_ 0.48–6.30 μM **2a**–**g**); **1g** and **2e**–**g** also appreciably inhibited PTP1B at micromolar concentrations. Propanoic derivatives **2e**–**g** significantly stimulated glucose uptake in C2C12 myocytes, in an insulin-independent way, reduced lipid accumulation in HepG2 liver cells, and caused hyperpolarization of C2C12 mitochondria at 10 μM concentration. Derivative **2e** significantly reduced sorbitol accumulation in both HLE and MIO-M1 cells at a 5 μM concentration. **Conclusions**: The results reported here provided new insights into the mechanisms of action and structure/activity relationships of 4-thiazolidinone derivatives, underscoring the capability of compounds **2e**–**g** of eliciting insulin-mimetic effects independent of hormone signalling. Among them, compound **2e** also proved to inhibit AKR1B1-dependent sorbitol accumulation and, thus, emerged as a promising multitarget agent that can be considered for further investigations.

## 1. Introduction

The development of several serious pathologies, such as diabetes, cancer, and cardiovascular and neurodegenerative diseases, involves complex etiological processes, simultaneously including diverse pathogenic alterations mediated by multiple targets. For this reason, the management of these diseases by single-targeted drug therapies is often unsatisfactory, and, therefore, drug combinations are required to improve clinical efficacy by controlling different mechanisms implicated in the course of the disease. However, combination therapies may give rise to complications regarding drug–drug interactions, pharmacokinetics, toxicity, and patient compliance. Therefore, in recent years, multitargeted ligands have attracted considerable attention for developing possible alternatives to drug combinations in the hope that they could provide enhanced efficacy, lower risk of adverse reactions, technological advantages, and improved adherence to long-term treatments [1,2,3,4]. In this context, designed multiple ligands (DMLs) are molecules rationally designed to be simultaneously directed at two or more biological targets, which are selected because of their proven implications in the pathogenesis of a certain disease [1]. The discovery of DMLs is generally based on well-established knowledge of the structures of the selected targets and/or of the pharmacophoric features of their ligands; in fact, a feasible medicinal chemistry approach consists of merging the pharmacophoric moieties of ligands directed to each target in a single “hybrid” molecule [1,3,5]. The subsequently performed optimization involves several challenging steps, in which different strategies may be exploited with the aim of balancing the effectiveness of the DML towards the selected biomolecules and minimizing its undesired activities towards irrelevant targets, in addition to modulating its drug-like properties [1,3].

Among the multifactorial diseases that could benefit from the availability of DMLs as novel drugs, diabetes mellitus (DM) represents one of the most serious global health threats. Currently, about 590 million adults are estimated to suffer from DM, and their number is predicted to further increase to more than 850 million by 2050. Type 2 DM (T2DM) accounts for more than 96% of DM cases worldwide, with an alarming increase not only among adults but also among adolescents [6,7,8]. DM is a chronic disease characterized by insulin resistance and/or insufficient insulin secretion, which are responsible for hyperglycaemia. This latter can compromise various cellular functions, thus causing tissue damage and promoting the development of severe complications, such as retinopathy, nephropathy, neuropathies, and cardiovascular pathologies. For these reasons, DM is included among the leading causes of death and disability worldwide, and growing attention is required to improve the management of this disease as well as the prevention of its complications.

In the treatment of DM, the main therapeutic aim is the control of hyperglycaemia, which, however, represents a challenging goal and, in the case of T2DM, often requires drug combinations. On this basis, the search for DMLs that can be directed to control multiple dysfunctions implicated in the development of T2DM is considered an attractive approach to achieve novel therapeutic options.

In the last few years, we have focused on the search for new DMLs as potential antidiabetic agents by investigating 4-thiazolidinone derivatives targeting both protein tyrosine phosphatase 1B (PTP1B) and aldose reductase (AKR1B1), which are enzymes critically implicated in specific signalling alterations underlying the development of T2DM and its chronic complications [9,10,11].

PTP1B exerts pivotal functions in the control of insulin signalling, mainly via the dephosphorylation of specific phosphotyrosine residues of the insulin receptor. Alterations of the action or expression of PTP1B were shown to be critically implicated in the development of T2DM by promoting and sustaining insulin resistance in both the central nervous system and peripheral tissues [12,13]. PTP1B overexpression was also found to be responsible for resistance to leptin, a hormone produced by adipocytes that exerts anorexigenic effects in the hypothalamus and promotes energy expenditure [14,15]. Both insulin and leptin play pivotal roles in the central nervous system by regulating glucidic and energetic homeostasis; their coordinated actions in the hypothalamus are also crucial for metabolism in peripheral tissues [16]. PTP1B inhibition proved to be an effective strategy to improve the cellular signalling of both hormones and, therefore, the search for inhibitors of this enzyme could pave the way for the development of novel therapeutic agents for the management of T2DM and its associated pathologies, such as obesity and metabolic syndrome [17,18,19].

**Figure 1:**
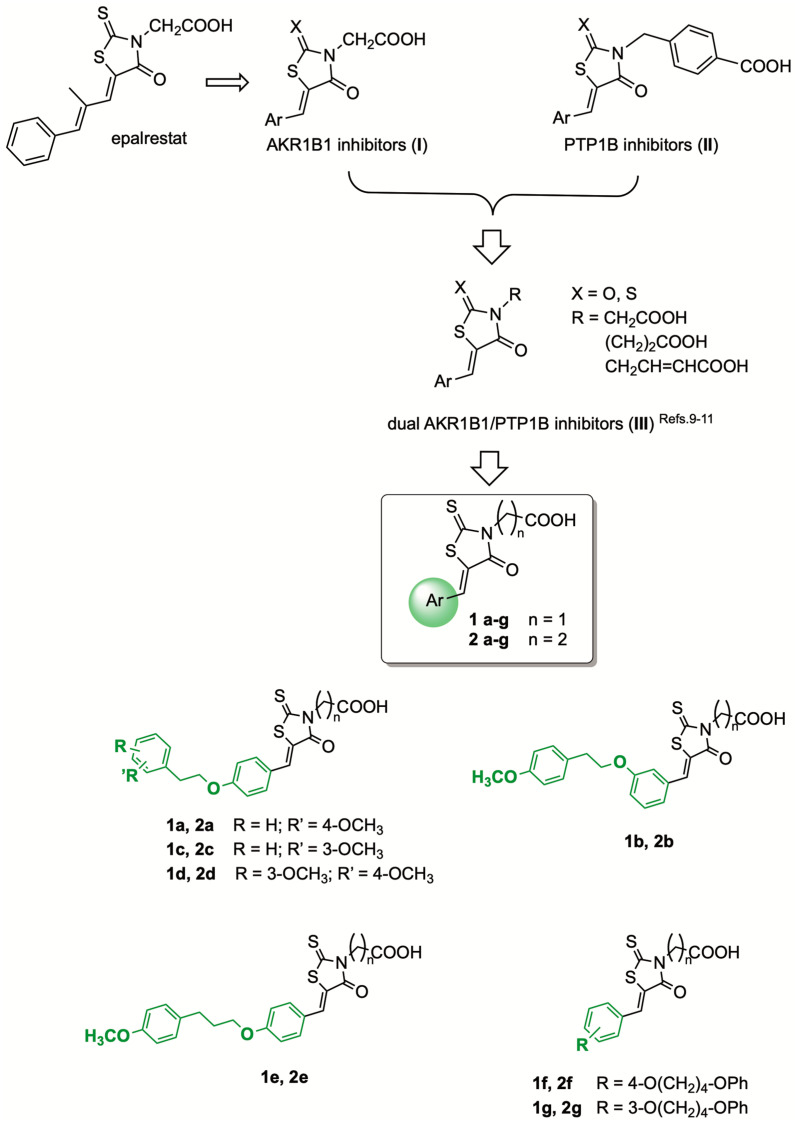
Design and structures of (5-arylidene-4-oxothiazolidin-3-yl)alkanoic acids **1a**–**g** and **2a**–**g**.

AKR1B1 is crucially involved in the etiopathology of diabetic long-term complications, since it catalyzes the first step of the polyol pathway, i.e., the NADPH-dependent reduction of glucose to sorbitol. Under hyperglycaemic conditions, the markedly increased AKR1B1-mediated metabolism of glucose through the polyol pathway promotes oxidative stress and inflammatory signalling, consequently triggering cellular dysfunctions and tissue damage, which are major causes of the onset and progression of chronic diabetic complications [20,21]. In fact, the inhibition of AKR1B1 proved to be an effective strategy to control hyperglycaemia-induced damage and, thus, to prevent or delay the development of DM-associated complications [20,21,22,23,24]. Among the numerous AKR1B1 inhibitors (ARIs) explored so far, the 4-oxo-2-thioxothiazolidine derivative epalrestat (Figure 1) is marketed in some Asian countries, such as Japan, for treating diabetic neuropathy [22].

Although PTP1B and AKR1B1 belong to different enzyme families and play different roles, some shared structural features can be observed in diverse ligands of these enzymes, thus suggesting that dual inhibitors could be designed. In fact, (5-arylidene-4-oxothiazolidin-3-yl)acetic acid derivatives (**I**, Figure 1), which were shown to act as potent ARIs [25], and 4-[(5-arylidene-4-oxothiazolidin-3-yl)methyl]benzoic acid derivatives (**II**, Figure 1), which exhibited significant PTP1B inhibitory properties [26], share a pharmacophoric acidic moiety (in position 3 of the thiazolidinone scaffold), and a hydrophobic portion (on C-5 of the heterocycle). On this basis, according to a knowledge-based multitarget approach, we recently obtained dual AKR1B1/PTP1B inhibitors (**III**, Figure 1) by merging the pharmacophores of 4-thiazolidinones of series **I** and **II**. Among derivative **III**, several compounds proved to act as dual AKR1B1/PTP1B inhibitors at low micromolar or submicromolar concentrations [9,10,11]. Interestingly, some of them also displayed significant cellular activities, such as insulin-sensitizing/mimetic effects in cultured C2C12 myoblasts as well as the capability of reducing intracellular sorbitol content in cultured human lens epithelial cells [10,11].

Pursuing this ongoing search for DMLs as potential antidiabetic agents, here we report a new series of (5-arylidene-4-oxothiazolidin-3-yl)alkanoic acids (**1a**–**g** and **2a**–**g**, Figure 1), synthesized with the aim of acquiring further insights into the role played by the 5-arylidene moiety on the activity profile of this class of DMLs. Starting from previously acquired data, which indicated that an extension of this portion may be beneficial for tuning the dual AKR1B1/PTP1B inhibitory activity, in compounds **1a**–**g** and **2a**–**g** the 5-arylidene moiety was modified by inserting one or two methoxy substituents on the distal phenyl ring and/or elongating the linker chain between the two aromatic rings. These structural modifications led to the identification of compounds capable of eliciting cellular insulin-mimetic effects independent of the signal transduction of the hormone and, simultaneously, to strongly inhibit AKR1B1.

## 2. Results and Discussion

### 2.1. Chemistry

**Scheme 1:**
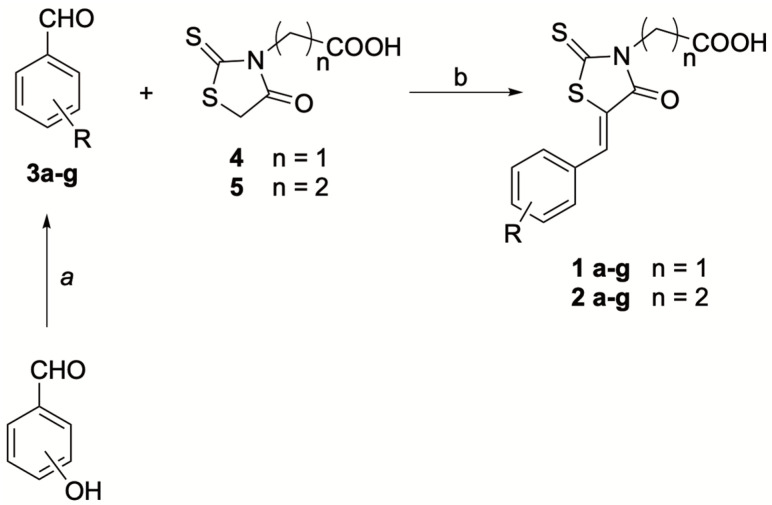
Synthesis of (5-arylidene-4-oxo-2-thioxothiazolidin-3-yl)acetic acids **1a**–**g** and 3-(5-arylidene-4-oxo-2-thioxothiazolidin-3-yl)propanoic acids **2a**–**g**. Reagents and conditions: (a) arylalkyl bromide, K_2_CO_3_, anhydrous DMF, 50–70 °C; (b) CH_3_COOH, CH_3_COONa, Δ.

(5-Arylidene-4-oxo-2-thioxothiazolidin-3-yl)alkanoic acids **1a**–**g**, **2a**–**g** were prepared by means of a convenient two-step synthesis, as depicted in Scheme 1. The reaction of *O*-alkylation of 3-hydroxybenzaldehyde or 4-hydroxybenzaldehyde with the appropriate arylyalkyl bromide, in the presence of potassium carbonate, produced arylalkoxy-substituted benzaldehydes **3a**–**g**. Then, the Knoevenagel condensation of aldehydes **3a**–**g** with (4-oxo-2-thioxothiazolidin-3-yl)acetic acid (**4**) or 3-(4-oxo-2-thioxothiazolidin-3-yl)propanoic acid (**5**), carried out in refluxing glacial acetic acid, in the presence of sodium acetate, provided compounds **1a**–**g** and **2a**–**g**, respectively (Scheme 1).

The structures of compounds **1a**–**g** and **2a**–**g** were assigned on the basis of analytical data and NMR spectroscopy (see Section 3 and Figures S1–S28). Besides standard ^1^H and ^13^C NMR 1D spectra, experiments with 2D techniques, such as ^1^H homocorrelated COSY and ^1^H-^13^C heterocorrelated gHSQCAD, were also performed to accomplish the unambiguous assignment of signals.

In the ^1^H NMR spectra, a singlet at 4.52–4.74 ppm, attributable to the methylene group on N-3 of acetic acid derivatives **1a**–**g**, or two coupled triplets at 2.61–2.64 ppm and 4.12–4.23 ppm, due to the resonance of the propanoic chain on N-3 of derivatives **2a**–**g**, were diagnostic. The carbon atoms of these methylene groups gave rise to signals in the range between 42.5 ppm and 47.3 ppm for acetic acid derivatives **1** and between 31.4 ppm and 69.6 ppm for the propanoic analogues **2**. Moreover, in the ^13^C NMR spectra of all synthesized compounds **1a**–**g**, **2a**–**g**, other diagnostic signals were due to the resonance of the carbonyl and thiocarbonyl groups of the (4-oxo-2-thioxothiazolidin-3-yl)alkanoic scaffold, in particular, a singlet at 193.6–193.8 ppm, attributable to the thiocarbonyl group in position 2 of the heterocyclic core, and two different singlets in the range between 166.8 ppm and 172.3 ppm, attributable to the carbonyl group in position 4 of the thiazolidinone and the carboxylic group of the alkanoic chain.

All derivatives **1**, **2** were obtained only as *Z* isomers; in fact, their ^1^H NMR and ^13^C NMR spectra showed only one set of signals, including a diagnostic singlet in the range 7.69–7.86 ppm of ^1^H-NMR spectra, which can be attributed to the resonance of the 5-methylidene proton.

### 2.2. In Vitro Enzyme Inhibition

The in vitro inhibitory activity of compounds **1a**–**g** and **2a**–**g** was evaluated against both human recombinant PTP1B, by using *p*-nitrophenylphosphate as substrate, and human recombinant AKR1B1, by using L-idose as substrate. Sodium metavanadate and sorbinil were the respective reference drugs.

Compounds **1a**–**g** and **2a**–**g** behaved as potent ARIs, showing excellent inhibitory ability against AKR1B1 with nanomolar or low micromolar IC_50_ values (Table 1). Acetic acid derivatives **1a**–**g** showed IC_50_ values lower than that of sorbinil, in the range between 0.03 μM (**1a**, **1g**) and 0.46 μM (**1e**). When the acetic chain on N-3 was replaced by the propanoic acid residue (compounds **2a**–**g**), less marked AKR1B1 inhibitory ability was observed (Table 1), with a decrement ranging from very slight (2e vs. 1e 1.5-fold) to significant (2b vs. 1b almost 78-fold); however, all compounds **2a**–**g** produced appreciable AKR1B1 inhibition, with IC_50_ values between 0.48 μM and 6.30 μM.

Among derivatives **1a**–**e**, which are methoxy-substituted on the distal phenyl ring of the 5-arylidene moiety, 4-[2-(4-methoxyphenyl)ethoxy]benzylidene-substituted compound **1a** was the most potent AKR1B1 inhibitor (IC_50_ = 0.03 μM); its isomers **1b** and **1c** were slightly less active (1.3-fold and 2.7-fold, respectively). Moreover, 3,4-dimethoxy-substituted analogue **1d** was shown to be 4-fold and 1.5-fold less effective than **1a** and **1c**, respectively, thus indicating that a methoxy group in the *meta* position of the distal phenyl ring is less tolerated than in the *para* position. Moreover, the insertion of a methylene in the linker chain was detrimental, leading to a 3-arylpropoxy derivative, **1e**, which was 15-fold less active than its 2-arylethoxy counterpart **1a** (Table 1).

A similar trend was observed in propanoic acid analogues **2a**–**e**, with 3,4-dimethoxy substituted analogue **2d**, which proved to be the least effective (IC_50_ = 6.30 μM) among all AKR1B1 inhibitors **1**, **2** (Table 1). However, in series **2**, it is worth noting that the 4-[3-(4-methoxyphenyl)propoxy]benzylidene-substituted derivative **2e** displayed significant AKR1B1 inhibitory activity (IC_50_ = 0.69 μM), similar to that of 2-(4-methoxyphenyl)ethoxy analogue **2a** (IC_50_ = 0.48 μM).

Moreover, phenoxybutoxy-substituted analogues **1f**, **1g**, and **2f** proved to be from 2- to 51-fold more potent AKR1B1 inhibitors than their phenylbutoxy counterparts, which we investigated previously [11].

Regarding PTP1B, acetic acid derivatives **1a**–**g** were found to be moderately or scarcely active toward the enzyme; compound **1g** showed the most appreciable inhibitory effect with an IC_50_ value of 27.7 μM (Table 1). Among propanoic acid analogues **2**, compounds **2e**–**g** were the most interesting PTP1B inhibitors, showing IC_50_ values in the range between 12.1 μM and 24.6 μM (Table 1). Comparing the PTP1B inhibitory ability of compounds **1**, **2** with that of the previously reported analogues [9,10,11] provided further evidence that the 5-arylidene moiety plays a crucial role in determining the PTP1B inhibitory effectiveness of (5-arylidene-4-oxothiazolidin-3-yl)alkanoic acid derivatives. In fact, the insertion of one or two methoxy groups on the distal phenyl ring of the phenylethoxy tail was detrimental, leading to analogues with moderate or marginal effects on PTP1B activity in both acetic and propanoic acid series **1a**–**d** and **2a**–**d**. On the other hand, the 4-[3-(4-methoxyphenyl)propyloxy]benzylidene derivative **2e** showed a noteworthy PTP1B inhibitory activity (IC_50_ = 12.1 μM), also proving to be the most potent PTP1B inhibitor among all compounds **1**, **2**; its activity toward PTP1B was appreciably higher than that of the previously reported 3-phenylpropyloxy analogue, which was almost inactive at a concentration of 5 μM [11]. Overall, comparing our current and previous data relative to both (5-arylidene-4-oxo-2-thioxothiazolidin-3-yl)acetic acid and 3-(5-arylidene-4-oxo-2-thioxothiazolidin-3-yl)propanoic acid series showed that the elongation of the linker connecting the two phenyl rings of the 5-arylidene moiety can, in general, improve PTP1B inhibitory effectiveness, but also suggests the existence of a cut-off which might correspond with the length of a pentatomic chain.

Out of the newly synthesized alkanoic acid derivatives **1**, **2**, compound **2e** stood out as the most interesting dual AKR1B1/PTP1B inhibitor, because of its significant ability to inhibit both target enzymes at submicromolar and low micromolar concentrations, respectively (Table 1). Compounds **2f** and **2g** also showed appreciable activity profiles, thus deserving further investigation along with **2e**. Analogue **1g** was also selected for additional assays, because it showed excellent AKR1B1 inhibitory effectiveness along with PTP1B inhibitory potency similar to that of **2f** and **2g**.

### 2.3. Kinetic Studies

**Figure 2:**
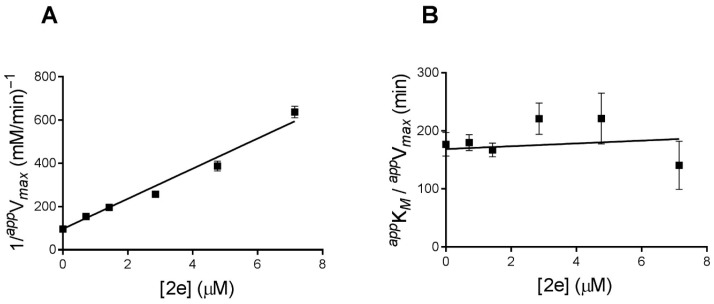
Kinetic characterization of compound **2e** as an AKR1B1 inhibitor. Kinetic measurements reported in Supplementary Figure S29 were used. Panel (**A**) refers to the 1/*^app^V_m_*_ax_ versus [I] plot. Panel (**B**) refers to the *^app^K_M_*/*^app^V_max_* versus [I] plot. Error bars represent the standard deviations of the mean from at least three independent experiments.

**Figure 3:**
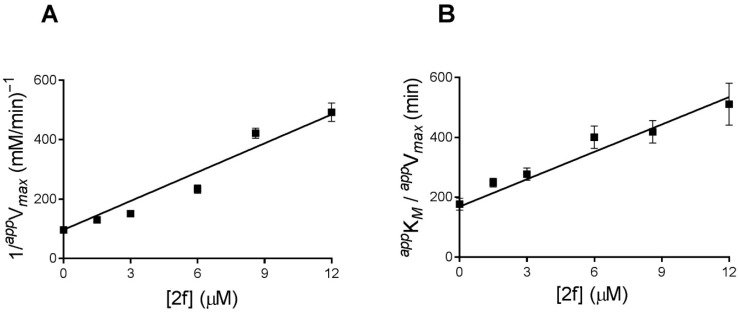
Kinetic characterization of compound **2f** as an AKR1B1 inhibitor. Kinetic measurements reported in Supplementary Figure S30 were used. Panel (**A**) refers to the 1/*^app^V_m_*_ax_ versus [I] plot. Panel (**B**) refers to the *^app^K_M_*/*^app^V_max_* versus [I] plot. Error bars (when not visible are within the symbol size) represent the standard deviations of the mean from at least three independent experiments.

**Figure 4:**
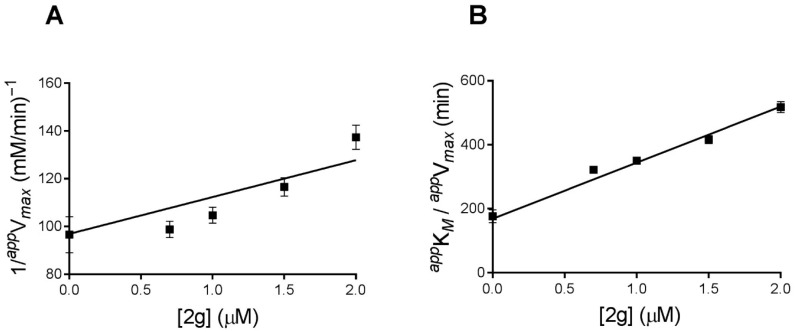
Kinetic characterization of compound **2g** as an AKR1B1 inhibitor. Kinetic measurements reported in Supplementary Figure S31 were used. Panel (**A**) refers to the 1/*^app^V_m_*_ax_ versus [I] plot. Panel (**B**) refers to the *^app^K_M_*/*^app^V_max_* versus [I] plot. Error bars (when not visible are within the symbol size) represent the standard deviations of the mean from at least three independent experiments.

**Figure 5:**
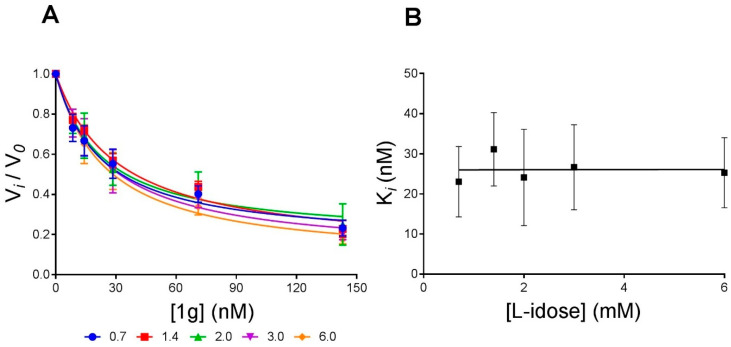
Kinetic characterization of compound **1g** as an AKR1B1 inhibitor. Panel (**A**): experimental kinetic data (see Supplementary Figure S32) were fitted to the Morrison equation (see Section 3). The numbers alongside the symbols refer to the L-idose concentration used in the assay. Error bars (when not visible are within the symbol size) represent the standard deviations of the mean from at least three independent experiments. Panel (**B**): The apparent inhibition constant (K_i_) values were plotted against substrate concentration and fitted by nonlinear regression analysis to Equation (1) (see Section 3). Error bars (when not visible are within the symbol size) represent the standard deviations of the mean from at least three independent experiments.

Compounds **1g** and **2e**–**g** were further characterized to assess their mechanism of action toward AKR1B1. Based on the measured IC_50_ values, compound **1g** was considered a tight-binding inhibitor. The experimental data of the reaction rates measured at different L-idose concentrations in the presence of different inhibitor concentrations are reported in the Supplementary Materials (Figures S29–S32). The secondary plots of ^app^V_max_ and ^app^K_M_ for compounds **2e**–**g** are reported in Figure 2, Figure 3 and Figure 4. For compound **1g**, the primary kinetic data (Figure S32) were fitted to the Morrison equation (Figure 5A), and the secondary plot of K_i_ as a function of L-idose concentration is reported in Figure 5B. For all the selected compounds, the resulting K_i_ and K′_i_ values are reported in Table 2. From these values, it turned out that compounds **2f** and **2g** acted as mixed-type inhibitors, while **2e** behaved as an uncompetitive inhibitor. The most potent analogue, **1g**, acted as a pure noncompetitive inhibitor, with almost identical K_i_ and K′_i_ values (Table 2).

**Figure 6:**
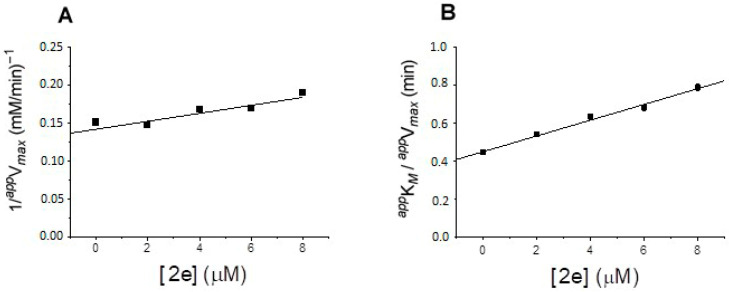
Kinetic characterization of compound **2e** as a PTP1B inhibitor. Panel (**A**) and Panel (**B**) report the secondary plots of 1/*^app^V_m_*_ax_ (Panel (**A**)) and *^app^K_M_*/*^app^V_max_* (Panel (**B**)), obtained from primary plots of kinetic measurements (see Supplementary Materials), as a function of the inhibitor concentration. Error bars (when not visible, they are within the symbol size) represent the standard deviations of the mean from at least three independent experiments.

To determine the mechanism of PTP1B inhibition by compounds **1g** and **2e**–**g**, further kinetic analyses were performed. A dilution assay with the selected compounds showed that PTP1B fails to recover enzymatic activity after extensive dilution in the assay buffer following incubation with compounds **1g**, **2f**, and **2g** (Figure S33). This finding suggests that these compounds might behave as irreversible or slow-binding inhibitors. To clarify this aspect, we analyzed the time course of PTP1B hydrolysis rates after diluting an aliquot of the enzyme into samples containing increasing concentrations of compounds **1g**, **2f**, and **2g**. The data obtained are presented in Figures S34–S36. In all cases, the hydrolysis rate of the control sample appeared to be linear over the observation period, indicating that the steady-state rate was reached immediately. In contrast, in the presence of compounds **1g**, **2f**, and **2g**, a lag time was observed before the full onset of inhibition, suggesting that these compounds act as slow-binding inhibitors. Furthermore, additional tests were carried out using a fixed inhibitor concentration and increasing concentrations of pNPP to evaluate whether the compounds act as competitive or non-competitive inhibitors. In the case of compound **1g**, we observed that PTP1B activity remained low at 5 and 10 mM pNPP concentrations, while it strongly increased at 20 mM pNPP (Figure S37). This indicates that a high substrate concentration can overcome the inhibitory effect caused by compound **1g**, suggesting that it acts as a competitive PTP1B inhibitor. Conversely, tests with both compounds **2f** and **2g** showed that the hydrolysis rate of PTP1B did not change with increasing pNPP concentrations, proving that these compounds behave as non-competitive inhibitors (Figures S38 and S39). Moreover, kinetic analyses revealed that the most active PTP1B inhibitor (compound **2e**) acts as a reversible, non-competitive mixed-type inhibitor (Figure 6 and Figures S40–S43). As concerns compounds **1g**, **2f**, and **2g**, the determination of Ki values was carried out using the equation derived from the appropriate kinetic models (see Supplementary Materials).

### 2.4. Molecular Docking Studies

AKR1B1 kinetic studies revealed that compounds **1g**, **2f**, and **2g** can interact with both the free enzyme and the ES complex, while compound **2e** can interact only with the ES complex. To mechanistically understand the binding mode, molecular docking studies were conducted on the AKR1B1 enzyme, targeting both the catalytic binding site and the AKR1B1-idose complex (PDB-ID: 3V36) [27]. The resulting docking poses were analyzed using structure-based 3D pharmacophore models [28].

**Figure 7:**
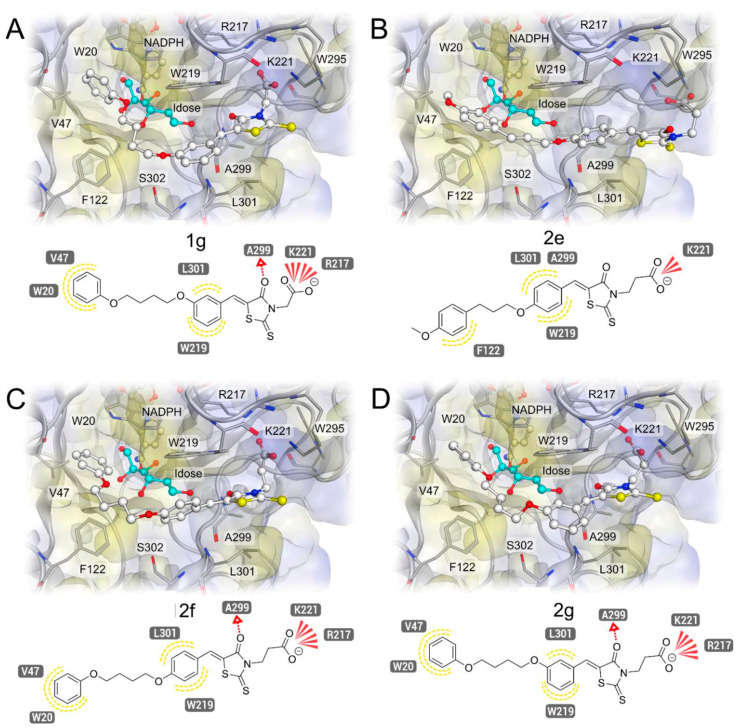
Compounds **1g** and **2e**–**g** docked into the AKR1B1-idose complex (PDB-ID: 3V36) [27]. The 2D and 3D structures of docked compounds (**A**) **1g**, (**B**) **2e**, which is further away from Arg217 due to the methoxy group on the terminal phenyl ring (**C**) **2f**, (**D**) **2g**. Colour codes: grey ribbon—AKR1B1, yellow protein surface—lipophilic surface, blue protein surface—hydrophilic surface.

Molecular docking studies in the AKR1B1-idose complex (Figure 7) revealed ionic interactions with Lys221 for all ligands.

Compounds **1g**, **2f**, and **2g** demonstrated an additional ionic interaction with Arg217. However, **2e** was unable to form this interaction due to the methoxy group on the terminal phenyl ring, which increases the size of the molecule and consequently distances the propionic acid moiety further away from Arg217 compared to the acid moieties of the compounds **1g**, **2f**, and **2g**. Therefore, these three compounds formed a hydrogen bond between the thioxothiazolidinone moiety and the backbone amide of Ala299, whereas **2e** formed a lipophilic contact between the 5-benzylidene ring and the side chain of Ala299. The 5-benzylidene ring in all compounds formed lipophilic contacts with Leu301 and Trp219. The terminal rings of **1g**, **2f**, and **2g** established hydrophobic contacts with Val47 and Trp20, whereas **2e** only showed a hydrophobic contact to Phe122 due to the methoxy group preventing the terminal phenyl ring from fitting deeper into the binding site. Docking experiments with **1g** and **2e**–**g** all resulted in reasonable docking poses, indicating that they fit into the AKR1B1-idose complex, which is in correlation to the kinetic studies (Figure 7, Table 3).

**Figure 8:**
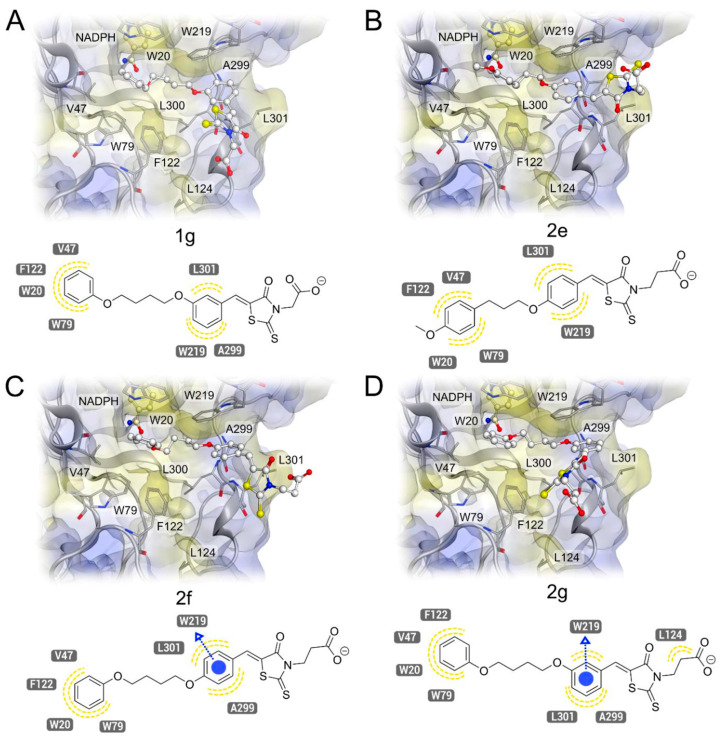
A 2D and 3D depiction of compounds **1g** and **2e**–**g** within the AKR1B1 catalytic binding site (PDB-ID: 3V36) [27]. The 2D and 3D structures of docked compounds (**A**) **1g**, (**B**) **2e**, (**C**) **2f**, and (**D**) **2g**. Colour code: grey ribbon—AKR1B1, yellow protein surface—lipophilic surface, blue protein surface—hydrophilic surface.

Docking to the catalytic binding site of AKR1B1 (Figure 8) showed that the terminal phenyl rings of compounds **1g** and **2e**–**g** all formed hydrophobic contacts in the catalytic binding site with the residues Trp20, Val47, Trp79, and Phe122. The compounds showed hydrophobic contacts between the 5-benzylidene ring and residues Trp219 and Leu301. Compounds **1g**, **2f**, and **2g** additionally interacted with Ala299. Aromatic π-stacking with Trp219 was only observed in mixed-type inhibitors **2f** and **2g**. This interaction was not present in the compounds **1g** and **2e** that act as pure non-competitive and uncompetitive inhibitors, respectively (Figure 8, Table 4).

**Figure 9:**
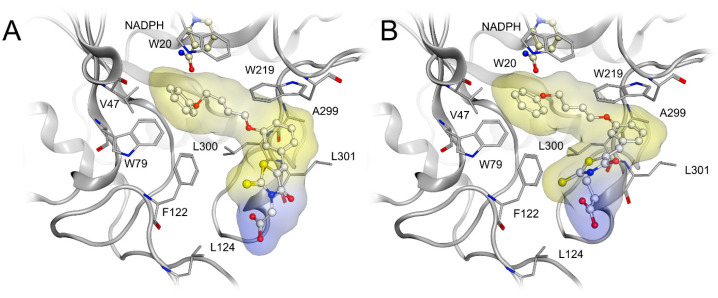
A 3D depiction of the molecular surfaces of compounds **1g** and **2g** within the AKR1B1 catalytic binding site (PDB-ID: 3V36) [27]. The 3D structures of the molecular surfaces of the docked compounds (**A**) **1g** and (**B**) **2g**, which are closer to the protein and allowed, due to the length of their acetic moieties, to perform an additional hydrophobic interaction with Leu124. Colour code: grey ribbon—AKR1B1, yellow molecule surface—lipophilic surface, blue molecule surface—hydrophilic surface.

Compounds **1g** and **2g** differ with respect to their molecular surfaces due to the varying lengths of their acetic acid and propionic acid moieties. The increased length of the propionic acid in **2g** allows this compound to form an additional hydrophobic contact with Leu124 that is not present in the docking pose of compound **1g**. Thus, a longer acid moiety is advantageous for binding in the catalytic site of AKR1B1 (Figure 9). This observation could explain the behaviour of **2g** as a mixed-type inhibitor with a prevalence of the competitive component in comparison to **1g**, which acts as a pure non-competitive inhibitor. Therefore, the molecular docking experiments suggest that both the aromatic interaction with Trp219 and the length of the acid moiety could be important for binding within the catalytic binding site of AKR1B1.

PTP1B kinetic studies revealed that compound **2e** acts as a mixed-type non-competitive inhibitor, while **2f** and **2g** act as pure non-competitive inhibitors, and **1g** acts as a competitive inhibitor. To obtain insights into the binding mode with the target enzyme, molecular docking experiments were performed on both the PTP1B catalytic binding site (PDB ID: 1Q6T) [29] and the previously described allosteric binding site of PTP1B [9,10,11].

**Figure 10:**
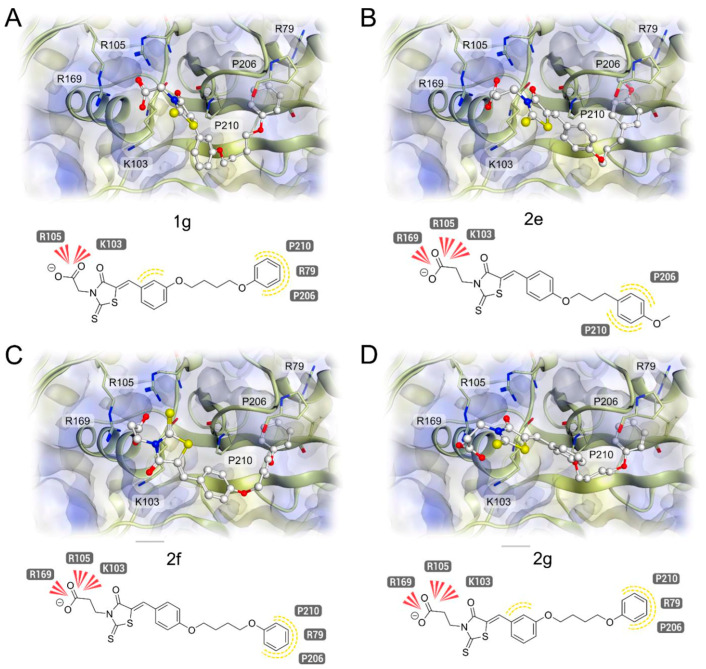
Compounds **1g** and **2e**–**g** docked into the PTP1B allosteric binding site (PDB-ID: 1Q6T) [29]. The 2D and 3D structures of docked compounds (**A**) **1g**, which, due to the length of the acetic acid moiety, is unable to perform an ionic interaction with Arg169. (**B**) **2e**, (**C**) **2f**, and (**D**) **2g**. Colour code: light brown ribbon—PTP1B, yellow protein surface—lipophilic surface, blue protein surface—hydrophilic surface.

The obtained docking poses of the previously described allosteric binding site of PTP1B showed that all the compounds can establish comparable lipophilic contacts between the terminal phenyl ring and the residues Pro206 and Pro210 (Figure 10). Furthermore, the terminal phenyl rings of compounds **1g**, **2f**, and **2g** form additional hydrophobic contact with the side chain of residue Arg79. Additionally, compounds **1g** and **2g** showed a hydrophobic contact between the 5-benzylidene ring and the side chain of Lys103. The short acetic acid moiety does not allow the compound **1g** to fit deep into the binding pocket and thus shows only ionic interactions with Arg105 and Lys103. In contrast, compounds **2e**, **2f**, and **2g** have a propionic acid moiety and can fit deeper into the binding pocket by forming an additional ionic interaction with Arg169. The absence of this interaction provides insight into the mechanism of action of **1g** as a competitive inhibitor and underlines the preference for a propionic acid moiety for binding within the allosteric binding site of PTP1B (Figure 10, Table 5).

**Figure 11:**
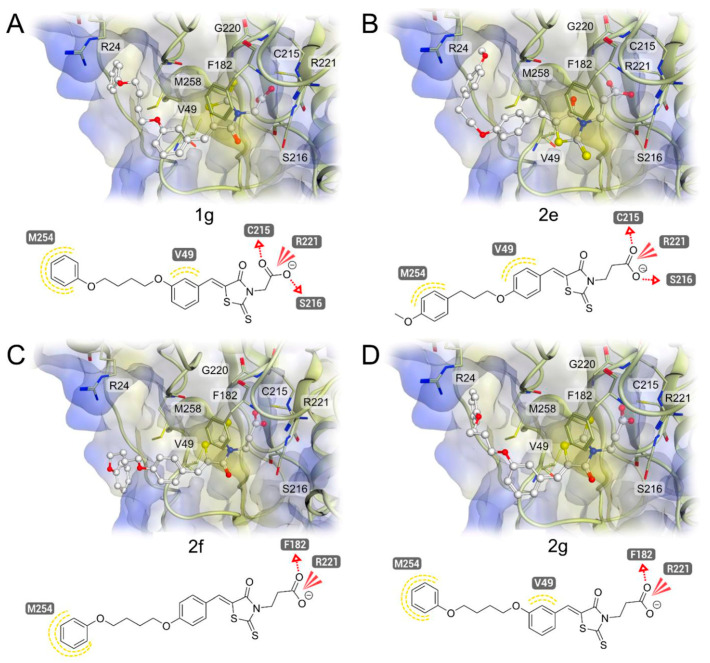
Compounds **1g** and **2e**–**g** docked into the PTP1B catalytic binding site (PDB-ID: 1Q6T) [29]. The 2D and 3D structures of docked compounds (**A**) **1g**, (**B**) **2e**, (**C**) **2f**, which, due to the location of the terminal phenyl ring, lose their hydrophobic contact with Val49. (**D**) **2g**. Colour code: light brown ribbon—PTP1B, yellow protein surface—lipophilic surface, blue protein surface—hydrophilic surface.

The molecular docking studies into the catalytic binding site of PTP1B revealed an ionic interaction of **1g** and **2e**–**g** with the side chain of Arg221 (Figure 11). Additionally, the terminal phenyl ring of all compounds formed a hydrophobic contact with the side chain of Met258. Compounds **2f** and **2g** showed that the propionic acid moiety can form a hydrogen bond with the backbone amide of Phe182.

Furthermore, compounds **1g** and **2e** showed hydrogen bonds from their respective acid moieties to the backbone amides of Cys215 and Ser216, which are not present in either **2f** or **2g**, showing that an acetic acid moiety or a substitution in the *para*-position of the terminal phenyl ring is preferred for the achievement of hydrogen bond interactions with these residues. The tests of compound **2f** showed that its terminal phenyl ring was situated at a greater distance from Arg24 and was located at the bottom of the binding pocket, with the 5-benzylidene ring being located further away from Val49 due to the *para*-substitution of the 5-benzylidene ring pushing the terminal phenyl ring further away from the binding site. This resulted in the loss of the hydrophobic contact between the 5-benzylidene ring and the side chain of Val49. The hydrogen bond interactions formed by the acidic moiety with the backbone amides of both Cys215 and Ser216, which were present in compounds **1g** and **2e**, are crucial factors that affect the activity of the compounds within the catalytic binding site of PTP1B. Consequently, this molecular docking study explains the pure non-competitive inhibitor behaviour of **2f** and **2g** (Figure 11, Table 6).

### 2.5. In Silico Pharmacokinetic and Toxicity Studies

The assessment of its pharmacokinetic (absorption, distribution, metabolism, elimination) and toxicity profiles (ADMET) is crucial to determine the potential of a bioactive molecule to be further developed as a drug candidate. In the initial phases of drug discovery, in silico models can be valuable tools to anticipate the drug-like properties of active compounds by evaluating certain molecular features, which can be predictive of the in vivo behaviour of a new agent.

Therefore, we carried out an in silico ADMET study of the most promising compounds, **1g**, **2e**, **2f**, and **2g**; epalrestat (EPA) was also included as a reference drug, being an orally bioavailable and generally well-tolerated drug, which is structurally related to derivatives **1** and **2**. We applied several computational algorithms, available on the SwissADME, PreADMET, and PhaKinPro platforms (available at www.swissadme.ch/index.php, https://preadmet.webservice.bmdrc.org, and https://phakinpro.mml.unc.edu, respectively; accessed on 21–24 January 2025), which incorporate predictive models for physico-chemical properties, drug-likeness, pharmacokinetics, and toxicity [30,31,32,33,34].

It is well-known that the ADMET profile of a bioactive compound is strictly related to certain physico-chemical properties and molecular descriptors, such as molecular weight (MW), polar surface area (PSA), lipophilicity (logP), water solubility, number of rotatable bonds (RB), and number of hydrogen bond donor (HBD) and hydrogen bond acceptor (HBA) groups. The well-accepted and widely applied “rule-of-5” was formulated by Lipinski as a qualitative predictor of absorption/permeability based on the analysis of some of the above-mentioned key properties of a wide library of compounds [32]. According to Lipinski’s rule, poor absorption or permeation is more likely when two or more of the following conditions are not met: MW ≤ 500, calculated logP (clogP) ≤ 5.0, number of HBD ≤ 5, number of HBA ≤ 10. Additional rules were proposed by Veber, based on further structural properties that were shown to increase oral bioavailability in animals; according to Veber’s rules, good oral bioavailability is likely for compounds with RB ≤ 10, PSA ≤ 140 Ǻ, and total hydrogen bonds (HBA + HBD) ≤ 12 [33]. Moreover, Ghose and coll. reported a qualitative and quantitative characterization of known drug libraries, such as the Comprehensive Medicinal Chemistry (CMC) database [34], from which drug-likeness qualifying ranges (covering more than 80% of compounds) were deduced for several parameters, such as clog P (between −0.4 and 5.6), MW (between 160 and 480), total number of atoms (between 20 and 70) [34].

Based on the above-described rules, implemented in both the SwissADME and PreADMET platforms, compounds **1g**, **2e**, **2f**, and **2g** were qualified as drug-like compounds. As reported in Table 7, calculated parameters of these compounds met the criteria of Lipinski’s rule without any violations; they also matched Veber’s rule, with only one violation for compounds **2f** and **2g** (RB > 10). All tested compounds also met Ghose’s rules, whereas their TPSA value (133.46 Å^2^) was slightly higher than that indicated by Egan (131.6 Å^2^) as the highest value related to a good passive intestinal absorption [35]. However, the TPSA (topological polar surface area) parameter is calculated by using a fragmental method [36], without considering stereochemistry and molecular flexibility; therefore, the exposed polar surface and its effect on bioavailability might deviate from the in silico prediction.

Based on the set of values of the calculated descriptors, compounds **1g**, **2e**, **2f**, and **2g** showed a promising drug-likeness and, therefore, it can be expected that they are suitable for oral administration.

A bioavailability score of 0.56 was estimated by SwissADME for compounds **1g**, **2e**, **2f**, and **2g**; according to a study performed by Martin [37], this score was calculated by combining total charge, TPSA, and Lipinski’s filters. A score of 0.56 indicates a probability of 56% that the tested compounds achieve at least 10% oral bioavailability in rats or display measurable Caco-2 permeability.

In vitro human colorectal adenocarcinoma Caco-2 cell permeability is considered a reliable model for the prediction of oral drug absorption. The analysis of data from the above-mentioned programmes (Table 8) suggested appreciable Caco-2 permeability for compounds **1g**, **2e**, **2f**, and **2g**, which correlates with the reference EPA. Accordingly, the predicted oral bioavailability (F, which expresses the fraction of an orally administered agent that reaches systemic circulation; F = 1 indicates 100% bioavailability following an intravenous administration) was in the range from 0.5 F to 0.8 F (predicted oral bioavailability from 50% to 80%) for compounds **2e** and **2f** and higher than 0.8 F (>80%) for compounds **2g** and **1g**, whereas the value calculated for EPA was lower than 0.5 F (<50%) (Table 8) [30]. Therefore, the compounds under study might be endowed with better oral bioavailability compared to the analogue EPA; this prediction might be related to the higher lipophilicity due to the 5-arylidene moiety of compounds **1**, **2**, compared with the reference drug.

Moreover, it is worth noting that out of the compounds **1g**, **2e**, **2f**, and **2g**, none was predicted to act as a substrate for glycoprotein P (P-gp); since P-gp functions as an efflux pump, this feature could contribute to a good bioavailability prediction.

Compounds **1g**, **2e**, **2f**, and **2g** displayed moderate water solubility, with Log S values (calculated by using an implementation of the ESOL model) [31] ranging between −5.33 and −5.28. Their capability to cross the blood–brain barrier as well as their skin permeability were predicted to be scarce.

The prediction of interaction of potential drugs with cytochromes P450 (CYP) could also be interesting, since these isoenzymes play key roles in the metabolism of xenobiotics. Compounds **1g**, **2e**, **2f**, and **2g**, as well as EPA, appeared not to be metabolized by the isoform CYP2D6, whereas **1g**, **2f**, and **2g** might be weak substrates of CYP3A4 (Table 8); accordingly, the calculated hepatic stability at 60 min was >50% for all compounds under consideration. Moreover, the inhibition of major CYP isoforms, such as CYP2C19, CYP2C9, CYP2D6, and CYP3A4, could cause pharmacokinetic drug–drug interactions. Interestingly, compounds **1g**, **2e**, **2f**, and **2g** were generally predicted not to be good CYP inhibitors; however, isoform CYP3A4 might be inhibited by **2e** and **2f**, whereas analogue **1g** may inhibit CYP2C9 (Table 8).

The in silico models implemented in the PreADMET software predicted no carcinogenicity risk at 2 years in rat and mouse models, as well as very low acute toxicity in both animal and plant organisms for all tested compounds, analogous to the reference EPA (Table 9). A medium risk of hERG inhibition was calculated, which might be related to potential cardiotoxicity.

Overall, the predicted drug-likeness and ADMET profiles of the selected compounds **1g**, **2e**, **2f**, and **2g** were promising, encouraging the continuation of the research on these enzyme inhibitors.

### 2.6. Ex Vivo Assays

To evaluate the ability of selected compounds to increase cellular sensitivity to insulin, tests were carried out using C2C12 cells. First, we evaluated the cytotoxicity of compounds **1g**, **2e**, **2f**, and **2g** by incubating C2C12 myoblasts with increasing concentrations of these compounds for 24 h. After this time, cell viability was determined by means of an MTT assay. We observed that all compounds were well tolerated by C2C12 cells, as a significant reduction in cell viability was observed only at the highest doses (Figure S47).

**Figure 12:**
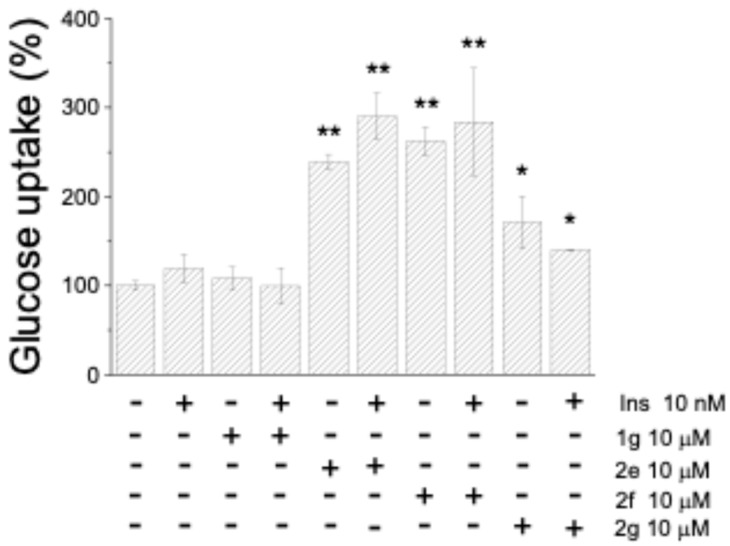
Glucose uptake assay. C2C12 cells grown on a p35 well were starved for 24 h and then treated for 30 min with starvation medium containing 10 nM insulin, and/or 10 μM of compounds **1g**, **2e**–**g**. After this period, cells were washed and incubated for 3 h with fresh medium containing 40 μM of fluorescent glucose. The cells were then washed, detached, and analyzed using a flow cytometer. For each test, at least 10,000 events were acquired. Data were normalized to the control test. Data reported in the figure represent the mean value ± SD (*n* = 3). The *t*-test was used to assess the statistical significance between the control and other groups. * *p* < 0.05; ** *p* < 0.01.

Then, we evaluated the capability of compounds **1g**, **2e**, **2f**, and **2g** to stimulate glucose uptake. C2C12 cells were treated with 10 nM insulin or 10 μM of each compound for 30 min and then incubated with 2-NBDG for 3 h. After this time, cells were detached and analyzed using a flow cytometry apparatus (Figure 12).

We found that compounds **2e** and **2f** strongly stimulated the glucose uptake when administered alone, while compound **2g** showed a weaker glucose uptake-stimulating activity. No differences in the intracellular glucose levels were found when cells were treated with a combination of compounds **2e**–**f** and insulin, thus suggesting that these compounds could act as insulin-mimetic agents. Finally, compound **1g** was not able to improve the cellular glucose uptake (Figure 12).

**Figure 13:**
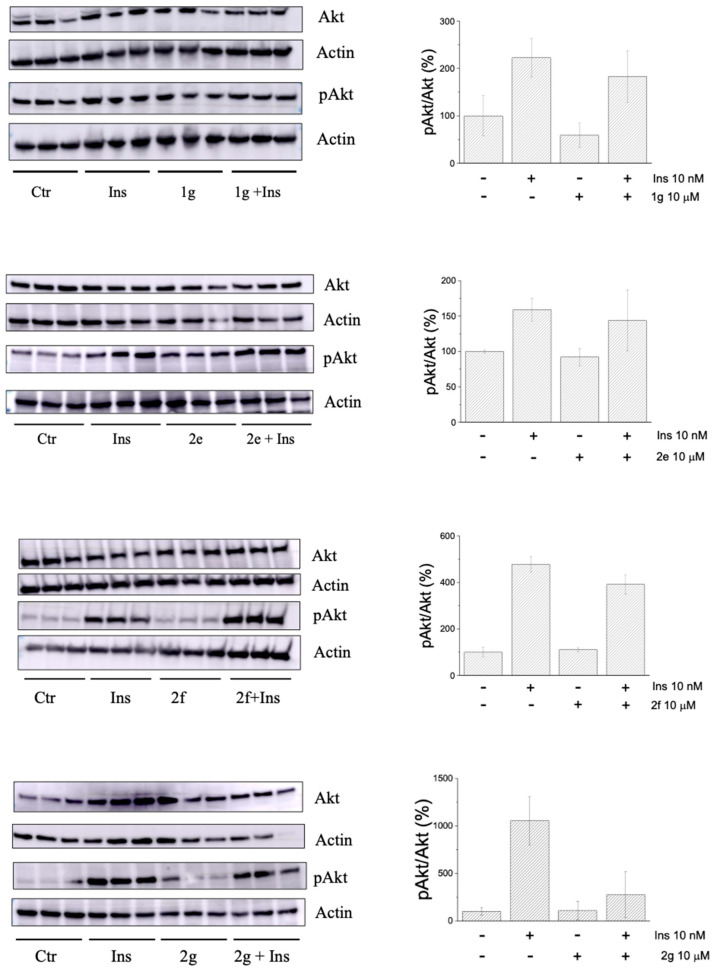
Effect of compounds **1g**, **2e**–**g** on the insulin signalling pathway. C2C12 cells were starved for 24 h and then stimulated for 30 min with 10 nM insulin, either with 10 μM of each compound **1g**, **2e**–**g**, or with a combination of insulin + compound. After 30 min, cells were lysed and protein extracts analyzed to evaluate the phosphorylation levels of kinase Akt by Western blot (figures on the left). Each test was carried out in triplicate. Data reported in the graphs on the right represent the mean value ± SD. All data were normalized with respect to the control sample.

Moreover, the ability of the selected compounds to improve the insulin signalling pathway was evaluated by monitoring the activation of the kinase Akt in the C2C12 cell line. (Figure 13). We found that none of the analyzed compounds could increase the phosphorylation status of the Akt kinase, either when administered alone (at 10 mM concentration) or in combination with insulin (Figure 13).

**Figure 14:**
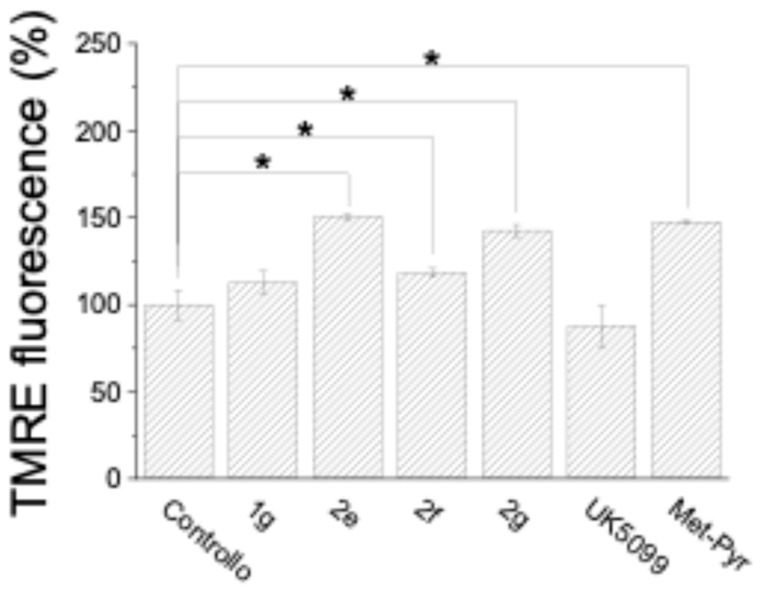
Evaluation of mitochondrial membrane potential with the TMRE probe. C2C12 cells were starved for 24 h and then incubated for 30 min with 10 μM compounds **1g**, **2e**–**g**, with 20 μM UK5099 or with 10 mM methylpyruvate. Then, cells were washed with PBS and incubated for a further 30 min with fresh starvation medium containing 0.2 μM TMRE. After, cells were analyzed using a flow cytometer. All tests were carried out in triplicate, and for each experiment, 10,000 events were analyzed. Data were analyzed using FlowJo v.10 (FlowJo) and normalized with respect to control cells. Data reported in the figure represent the mean value ± SD. The differences between the experimental and control groups were compared using the Student’s *t*-test. * *p* < 0.05.

Therefore, it can be inferred that the tested compounds can stimulate glucose uptake, as observed in C2C12 cells, by an insulin-independent mechanism. Recent studies reported that some 2,4-thiazolidinediones acutely inhibit mitochondrial pyruvate carrier, impairing cellular respiration and mitochondrial ATP production [38]. Based on this evidence, we wondered if 4-thiazolidinone derivatives **2e**–**g** could act in a similar manner. To address this question, we evaluated the mitochondrial potential in C2C12 cells acutely treated with compounds **2e**–**g** (Figure 14).

Data reported in Figure 14 show that the fluorescence of the TMRE probe incorporated into the mitochondria of C2C12 cells treated with compounds **2e**–**g** was significantly higher than that of the control cells and very similar to that observed after incubation of muscle cells with methyl pyruvate, a membrane-permeable ester of pyruvate. Instead, fluorescence levels like those of the control cells were observed following treatment with compound **1g**, whereas treatment with UK5099—a well-known inhibitor of the mitochondrial pyruvate carrier—resulted in a decrease in the mitochondrial membrane potential. This latter result was expected, as a previous study demonstrated that UK5099 impairs oxygen consumption when administered to C2C12 myoblasts [38]. The results of the TMRE fluorescence test suggest that the increased glucose uptake triggered by compounds **2e**–**g** could be related to the hyperpolarization of cellular mitochondria, by mimicking the effects observed following methyl pyruvate administration. A possible hypothesis is that compounds **2e**–**g** may target the mitochondrial pyruvate transporter [39], by stabilizing it in a conformation that facilitates the translocation of pyruvate into the mitochondria, thereby also enhancing cellular glucose uptake through a sort of “carryover effect.” Based on this hypothesis, we might expect that the mitochondria of myoblasts treated with compounds **2e**–**g** can produce greater amounts of reduced coenzymes (i.e., NADH and FADH_2_, produced by the Krebs cycle), whose subsequent oxidation via mitochondrial complexes I and II promotes an increase in mitochondrial membrane potential. However, if this assumption is correct, we would also expect that, as a consequence of the increased pyruvate flux into the mitochondria, acetyl-CoA synthesis would also increase, leading to the activation of pyruvate carboxylase, an enzyme responsible for oxaloacetate synthesis. Under these conditions, acetyl-CoA molecules derived from other metabolic pathways, such as β-oxidation, could be metabolized, resulting in a higher rate of fatty acid oxidation.

**Figure 15:**
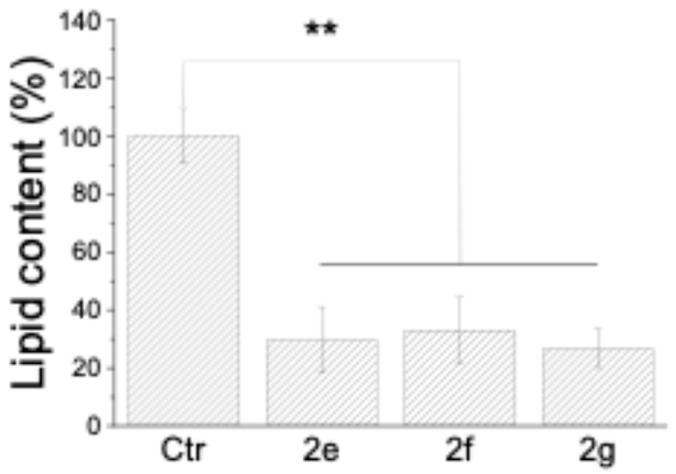
Lipid accumulation assay. HepG2 liver cells were treated with 0.4 mM oleic acid (OA) for 24 h, and after were treated with 10 μM compounds **2e**–**g** for a further 72 h. Then, the intracellular lipid content was determined by using the Oil Red O dye. All tests were carried out in quadruplicate. Data obtained were normalized with respect to protein content and reported as a percentage of the control test. The data shown in the figure represent the mean values ± SD. ** *p* < 0.01.

To confirm this hypothesis, we evaluated the ability of liver HepG2 cells, overloaded with fatty acids and then treated with compounds **2e**–**g**, to reduce the content of lipid droplets. We found that liver cells treated with compounds **2e**–**g** (10 μM) showed a significant reduction in lipid content with respect to untreated cells, therefore confirming that these compounds can boost fatty acid degradation, thus preventing lipid accumulation in hepatocytes (Figure 15 and Figure S48).

Taken together, these results suggest that 4-thiazolidinone derivatives **2e**–**g** might interact with the mitochondrial pyruvate transporter differently from analogous 2,4-thiazolidinediones by promoting pyruvate transport rather than inhibiting it.

Furthermore, human lens epithelial B3 line (HLE) cells and human retinal glial line (MIO-M1) cells were used as cellular models for the onset of high-glucose-induced cataract and retinopathy, respectively. Dual AKR1B1/PTP1B inhibitors **2e** and **2f** showed interesting effects in C2C12 myoblasts by significantly improving glucose uptake; therefore, it was also worth assessing their effect on high glucose-induced sorbitol accumulation. Compound **1g** was also included in these assays as the most potent AKR1B1 inhibitor among compounds **1a**–**g**, **2a**–**g** (Table 1).

**Figure 16:**
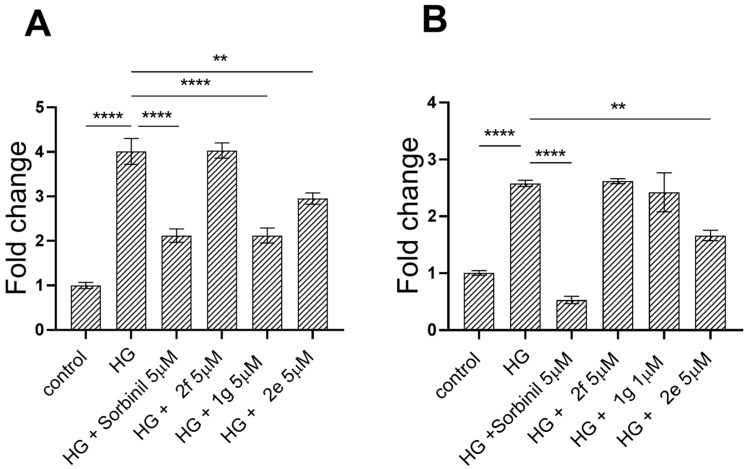
Effect of selected compounds on high-glucose-induced sorbitol accumulation. HLE (Panel **A**) and MIO-M1 (Panel **B**) cells were cultured for 48 h in the proper medium (see Section 3), (control), in the presence of 0.05% DMSO. HG refers to cells incubated as above in the presence of 75 mM D-glucose alone or in the presence of the indicated inhibitor concentrations. Sorbitol content for control cells accounted for 28.5 ± 3.2 and 33 ± 6.8 nmol/mg protein for HLE and MIO-M1 cells, respectively. Values are reported as the mean ± SEM of five and three independent measurements for HLE and MIO-M1 cells, respectively. Statistical analysis was performed using one-way Anova with the Dunnet post hoc test. Significance was evaluated with respect to HG (**: *p* < 0.01; ****: *p* < 0.0001).

The effect of compounds **1g**, **2e**, and **2f** on cell viability was tested. No toxicity was observed upon 48 h incubation of HLE cells in the presence of inhibitor concentrations of up to 5 μM (Figure S49). In the case of MIO-M1 cells, similar results were obtained, except for **1g**, which caused significant toxicity at a concentration of 5 μM (Figure S49). Thus, in further experiments, **1g** was used at a lower concentration (i.e., 1 μM), which did not affect the MIO-M1 cells’ viability (Figure S49). The ability of selected compounds to impair the sorbitol accumulation observed in HLE and MIO-M1 cells exposed to high glucose was evaluated, using Sorbinil as a reference inhibitor (Figure 16). Both cell lines were sensitive to compound **2e**, which was significantly effective in impairing sorbitol accumulation in both cell lines in a comparable manner, but exhibited complete insensitivity to compound **2f**. Compound **1g** significantly impaired sorbitol accumulation in HLE cells, but the lower concentration tested, due to its observed toxicity in MIO-M1 cells, did not allow any effect to be highlighted in this latter cell line. Finally, differences between the sensitivity of two cell lines to the reference drug were observed, since sorbinil was more effective in recovering the normal sorbitol content in MIO-M1 with respect to HLE cells (Figure 16).

## 3. Materials and Methods

### 3.1. Chemistry

Melting points were determined using a Kofler hot-stage apparatus and are reported without correction. Thin-layer chromatography (TLC) was performed on silica gel plates (Merck F254, Darmstadt, Germany), and Rf values were obtained employing appropriate mixtures of diethyl ether and *n*-hexane as eluents. Elemental analyses (C, H, N) were carried out on a Carlo Erba model 1106 elemental analyzer (Milan, Italy), with results within ±0.4% of the theoretical values. ^1^H and ^13^C NMR spectra were recorded on a Varian spectrometer (Palo Alto, CA, USA) operating at 500 MHz (499.74 MHz for ^1^H and 125.73 MHz for ^13^C). In addition to standard one-dimensional ^1^H and ^13^C NMR experiments, ^1^H–^1^H COSY and ^1^H–^13^C gHSQCAD correlation experiments were performed. Chemical shifts (δ) are reported in ppm and coupling constants (J) in Hz. Spectra were phased and baseline corrected as required. CDCl_3_ or DMSO-*d*_6_ served as internal references for both ^1^H and ^13^C spectra. Exchangeable protons were confirmed by D_2_O addition. Unless otherwise specified, all reagents were purchased from commercial suppliers and used without further purification. The purity of the synthesized compounds was confirmed to be ≥95% by elemental analysis.

#### 3.1.1. General Procedure for the Synthesis of Arylalkoxy Benzaldehydes **3a**–**g**

Aryl/aryloxyalkyl bromide (26.6 mmol) was added, in small portions at regular intervals of a few minutes, to a mixture of 3-hydroxybenzaldehyde or 4-hydroxybenzaldehyde (2 g, 16.4 mmol), and potassium carbonate (9.06 g, 65.6 mmol) in anhydrous DMF (75 mL); the mixture was stirred at 50–70 °C for 2–4 h. Then water (20 mL) was added, and the mixture was acidified to pH 5–6 and extracted with ethyl acetate. The organic layer was washed with H_2_O (10 × 100 mL), dried with anhydrous Na_2_SO_4,_ and the solvent was removed under reduced pressure. The crude mixture was separated by silica gel column chromatography, using a mixture of petroleum ether:diethyl ether = 9:1 or cyclohexane:diethyl ether = 8:2 as eluant, to provide pure aldehydes **3a**–**g**.

##### 4-[2-(4-Methoxyphenyl)ethoxy]benzaldehyde (**3a**)

Yield 61%; yellow oil; ^1^H NMR (CDCl_3_): δ 3.09 (t *J* = 7.05 Hz, 2H, CH_2_); 3.82 (s, 3H, OCH_3_); 4.23 (t *J* = 7.05 Hz, 2H, OCH_2_); 6.89–6.91 (m, 4H, arom); 7.02 (m, 2H, arom); 7.84 (m, 2H, arom); 9.88 (s, 1H, CHO). ^13^C NMR (CDCl_3_): δ 34.8, 67.0 (CH_2_); 55.4 (OCH_3_); 114.0, 114.6, 128.1, 129.0, 130.1, 131.5, 157.5, 164.9 (CH arom, Cq arom); 190.9 (C=O).

##### 3-[2-(4-Methoxyphenyl)ethoxy]benzaldehyde (**3b**)

Yield 36%; yellow oil; ^1^H NMR (CDCl_3_): δ 3.08 (t *J* = 7.0 Hz, 2H, CH_2_); 3.81 (s, 3H, OCH_3_); 4.21 (t *J* = 7.0 Hz, 2H, OCH_2_); 6.87–6.90 (m, 3H, arom); 7.22–7.24 (m, 2H, arom); 7.40–7.45 (m, 3H, arom); 9.97 (s, 1H, CHO). ^13^C NMR (CDCl_3_): δ 35.2, 67.0 (CH_2_); 55.7 (OCH_3_); 114.0, 116.0, 119.9, 121.3, 129.8, 129.9, 130.2, 137.4, 157.6, 165.1 (CH arom, Cq arom); 191.6 (C=O).

##### 4-[2-(3-Methoxyphenyl)ethoxy]benzaldehyde (**3c**)

Yield 23%; yellow oil; ^1^H NMR (CDCl_3_): δ 3.13 (t *J* = 7.0 Hz, 2H, CH_2_); 3.83 (s, 3H, OCH_3_); 4.27 (t *J* = 7.0 Hz, 2H, OCH_2_); 7.03–7.05 (m, 3H, arom); 7.26–7.29 (m, 2H, arom); 7.81–7.86 (m, 3H, arom); 9.86 (s, 1H, CHO). ^13^C NMR (CDCl_3_): δ 35.3, 67.0 (CH_2_); 55.5 (OCH_3_); 111.2, 114.0, 114.6, 120.2, 128.3, 129.6, 131.5, 138.1, 159.8, 165.0 (CH arom, Cq arom); 190.8 (C=O).

##### 4-[2-(3,4-Dimethoxyphenyl)ethoxy]benzaldehyde (**3d**)

Yield 45%; pale yellow oil; ^1^H NMR (CDCl_3_): δ 3.03 (t *J* = 6.95 Hz, 2H, CH_2_); 3.83 and 3.84 (2s, 6H, 2 OCH_3_); 4.19 (t *J* = 6.95 Hz, 2H, OCH_2_); 6.77–6.80 (m, 3H, arom); 6.94–6.97 (m, 2H, arom); 7.76–7.78 (m, 2H, arom); 9.81 (s, 1H, CHO). ^13^C NMR (CDCl_3_): δ 35.0, 66.8 (CH_2_); 55.9 (OCH_3_); 112.2, 112.8, 114.5, 121.8, 128.3, 130.6, 131.7, 146.5, 149.2, 165.0 (CH arom, Cq arom); 190.7 (C=O).

##### 4-[3-(4-Methoxyphenyl)propoxy]benzaldehyde (**3e**)

Yield 92%; pale yellow solid; m.p. 65 °C; ^1^H NMR (CDCl_3_): δ 2.10–2.14 (m, 2H, CH_2_); 2.78 (t *J* = 7.5 Hz, 2H, CH_2_); 3.80 (s, 3H, OCH_3_); 4.04 (t *J* = 6.3 Hz, 2H, OCH_2_); 6.85 (m, 2H, arom); 7.00 (m, 2H, arom); 7.13 (m, 2H, arom); 7.83 (m, 2H, arom); 9.89 (s, 1H, CHO). ^13^C NMR (CDCl_3_): δ 30.8, 31.0, 67.2 (CH_2_); 55.3 (OCH_3_); 113.9, 114.8, 129.4, 129.8, 132.0, 133.1, 157.9, 164.1 (CH arom, Cq arom); 190.8 (C=O).

##### 4-(4-Phenoxybutoxy)benzaldehyde (**3f**)

Yield 87%; white solid; m.p. 109–110 °C; ^1^H NMR (CDCl_3_): δ 1.96–2.09 (m, 4H, 2 CH_2_); 4.06 (t *J* = 5.8 Hz, 2H, OCH_2_); 4.14 (t *J* = 5.95 Hz, 2H, OCH_2_); 6.91–6.96 (m, 3H, arom); 7.01 (m, 2H, arom); 7.28–7.31 (m, 2H, arom); 7.84 (m, 2H, arom); 9.90 (s, 1H, CHO). ^13^C NMR (CDCl_3_): δ 25.9, 27.9, 67.2, 67.9 (CH_2_); 114.4, 114.7, 120.7, 129.4, 129.5, 132.0, 158.8, 159.6 (CH arom, Cq arom); 190.8 (C=O).

##### 3-(4-Phenoxybutoxy)benzaldehyde (**3g**)

Yield 63%; white solid; m.p. 98–99 °C; ^1^H NMR (CDCl_3_): δ 2.00–2.07 (m, 4H, 2 CH_2_); 4.06 (t *J* = 5.95 Hz, 2H, OCH_2_); 4.12 (t *J* = 6.0 Hz, 2H, OCH_2_); 6.91–6.95 (m, 3H, arom); 7.28–7.31 (m, 3H, arom); 7.40–7.47 (m, 3H, arom); 9.98 (s, 1H, CHO). ^13^C NMR (CDCl_3_): δ 25.9, 26.0, 67.2, 67.8 (CH_2_); 112.8, 114.5, 120.7, 121.9, 123.4, 129.5, 130.0, 137.8, 158.9, 159.6 (CH arom, Cq arom); 192.2 (C=O).

#### 3.1.2. General Procedure for the Synthesis of (5-Arylidene-4-oxo-2-thioxothiazolidin-3-yl)alkanoic Acids **1a**–**g** and **2a**–**g**

A mixture of (4-oxo-2-thioxothiazolidin-3-yl)acetic acid (**4**) (0.40 g, 2.09 mmol) or 3-(4-oxo-2-thioxothiazolidin-3-yl)propanoic acid (**5**) (0.43 g, 2.09 mmol) with the appropriate aldehyde **3a**–**g** (2.09 mmol) and sodium acetate (2.13 g, 15.7 mmol), in glacial acetic acid (10 mL), was heated to 100 °C for 4–5 h and subsequently maintained at room temperature overnight. The mixture was poured into H_2_O, and the obtained crude precipitate was filtered off, then washed with H_2_O and recrystallized from methanol to give pure acids **1a**–**g** and **2a**–**g**.

##### [(5Z)-5-({4-[2-(4-Methoxyphenyl)ethoxy]phenyl}methylidene)-4-oxo-2-thioxothiazolidin-3-yl]acetic Acid (**1a**)

Yield 46%; yellow solid; mp 281–284 °C; ^1^H NMR (DMSO-*d*_6_): δ 2.99 (t *J* = 6.9 Hz, 2H, CH_2_); 3.72 (s, 3H, OCH_3_); 4.24 (t *J* = 6.9 Hz, 2H, OCH_2_); 4.52 (s, 2H, NCH_2_); 6.87 (m, 2H, arom); 7.12 (m, 2H, arom); 7.24 (m, 2H, arom); 7.61 (m, 2H, arom), 7.78 (s, 1H, CH methylidene). ^13^C NMR (DMSO-*d*_6_): δ 34.4, 47.2, 69.4 (CH_2_); 55.6 (OCH_3_); 114.4, 116.2, 126.1, 130.4, 130.5, 133.5, 158.5, 161.3 (CH arom, Cq arom); 119.7 (C-5); 133.7 (CH methylidene); 167.3, 167.4 (C=O); 193.6 (C=S). Anal. (C_21_H_19_NO_5_S_2_) calcd: C 58.73; H 4.46; N 3.26; found: C 58.54; H 4.61; N 3.36.

##### [(5Z)-5-({3-[2-(4-Methoxyphenyl)ethoxy]phenyl}methylidene)-4-oxo-2-thioxothiazolidin-3-yl]acetic Acid (**1b**)

Yield 35%; yellow solid; mp 213–215 °C; ^1^H NMR (DMSO-*d*_6_): δ 2.99 (t *J* = 6.9 Hz, 2H, CH_2_); 3.72 (s, 3H, OCH_3_); 4.21 (t *J* = 6.9 Hz, 2H, OCH_2_); 4.54 (s, 2H, NCH_2_); 6.87 (m, 2H, arom); 7.09 (dd *J* = 8.25 and 2.15 Hz, 1H, arom); 7.19–7.26 (m, 4H, arom); 7.46 (dd *J* = 7.95 Hz, 1H, arom), 7.81 (s, 1H, CH methylidene). ^13^C NMR (DMSO-*d*_6_): δ 34.5, 47.1, 69.2 (CH_2_); 55.6 (OCH_3_); 114.3, 116.9, 123.0, 123.2, 130.5, 131.2, 134.9, 158.4, 159.5 (CH arom, Cq arom); 118.2 (C-5); 133.7 (CH methylidene); 167.1, 167.7 (C=O); 193.7 (C=S). Anal. (C_21_H_19_NO_5_S_2_) calcd: C 58.73; H 4.46; N 3.26; found: C 58.80; H 4.40; N 3.17.

##### [(5Z)-5-({4-[2-(3-Methoxyphenyl)ethoxy]phenyl}methylidene)-4-oxo-2-thioxothiazolidin-3-yl]acetic Acid (**1c**)

Yield 42%; yellow solid; m.p. 207–210 °C; ^1^H NMR (DMSO-*d*_6_): δ 3.01 (t *J* = 6.7 Hz, 2H, CH_2_); 3.79 (s, 3H, OCH_3_); 4.27 (t *J* = 6.7 Hz, 2H, OCH_2_); 4.71 (s, 2H, NCH_2_); 6.77 (dd *J* = 8.2 and 2.45 Hz, 1H, arom); 6.86–6.88 (m, 2H, arom); 7.09 (m, 2H, arom); 7.20 (dd *J* = 8 Hz, 1H, arom); 7.58 (m, 2H, arom), 7.78 (s, 1H, CH methylidene). ^13^C NMR (DMSO-*d*_6_): δ 35.6, 45.9, 69.4 (CH_2_); 55.8 (OCH_3_); 112.7, 115.5, 116.6, 122.1, 126.2, 130.3, 135.0, 140.6, 160.2, 161.9 (CH arom, Cq arom); 119.4 (C-5); 134.0 (CH methylidene); 167.4, 168.3 (C=O); 194.0 (C=S). Anal. (C_21_H_19_NO_5_S_2_) calcd: C 58.73; H 4.46; N 3.26; found: C 58.91; H 4.35; N 3.42.

##### [(5Z)-5-({4-[2-(3,4-Dimethoxyphenyl)ethoxy]phenyl}methylidene)-4-oxo-2-thioxothiazolidin-3-yl]acetic Acid (**1d**)

Yield 37%; yellow solid; mp 230–233 °C; ^1^H NMR (DMSO-*d*_6_): δ 2.98 (t *J* = 6.85 Hz, 2H, CH_2_); 3.70, 3.73 (2s, 6H, 2 OCH_3_); 4.25 (t *J* = 6.85 Hz, 2H, OCH_2_); 4.72 (s, 2H, NCH_2_); 6.81 (d *J* = 8.2 Hz, 1H, arom); 6.86 (d *J* = 8.2 Hz, 1H, arom); 6.93 (s, 1H, arom); 7.12 (m, 2H, arom); 7.61 (m, 2H, arom); 7.82 (s, 1H, CH methylidene). ^13^C NMR (DMSO-*d*_6_): δ 35.0, 45.7, 69.5 (CH_2_); 56.2, 56.3 (OCH_3_); 112.6, 113.6, 116.4, 121.6, 126.0, 131.1, 134.9, 148.1, 149.3, 161.7 (CH arom, Cq arom); 119.2 (C-5); 133.9 (CH methylidene); 167.2, 168.1 (C=O); 193.8 (C=S). Anal. (C_22_H_21_NO_6_S_2_) calcd: C 57.50; H 4.61; N 3.05; found: C 57.67; H 4.72; N 2.98.

##### [(5Z)-5-({4-[3-(4-Methoxyphenyl)propoxy]phenyl}methylidene)-4-oxo-2-thioxothiazolidin-3-yl]acetic Acid (**1e**)

Yield 37%; yellow solid; mp 238–242 °C; ^1^H NMR (DMSO-*d*_6_): δ 2.00 (m, 2H, CH_2_); 2.68 (t *J* = 7.5 Hz, 2H, CH_2_C_6_H_5_); 3.71 (s, 3H, OCH_3_); 4.04 (t *J* = 6.2 Hz, 2H, OCH_2_); 4.52 (s, 2H, NCH_2_); 6.84 (m, 2H, arom); 7.13–7.15 (m, 4H, arom); 7.61 (m, 2H, arom), 7.79 (s, 1H, CH methylidene). ^13^C NMR (DMSO-*d*_6_): δ 30.9, 31.0, 47.3, 67.7 (CH_2_); 55.5 (OCH_3_); 114.3, 116.2, 126.1, 129.8, 133.5, 133.6, 158.0, 161.5 (CH arom, Cq arom); 119.6 (C-5); 133.7 (CH methylidene); 167.3, 167.4, (C=O); 193.6 (C=S). Anal. (C_22_H_21_NO_5_S_2_) calcd: C 59.58; H 4.77; N 3.16; found: C 59.70; H 4.81; N 3.09.

##### 2-[(5Z)-5-{[4-(4-Phenoxybutoxy)phenyl]methylidene}-4-oxo-2-thioxothiazolidin-3-yl]acetic acid (**1f**)

Yield 62%; yellow solid; mp 217–220 °C; ^1^H NMR (DMSO-*d*_6_): δ 1.88 (m, 4H, 2 CH_2_); 4.03 (t *J* = 5.5 Hz, 2H, OCH_2_); 4.13 (t *J* = 5.6 Hz, 2H, OCH_2_); 4.52 (s, 2H, NCH_2_); 6.91–6.94 (m, 3H, arom); 7.13 (m, 2H, arom); 7.26–7.29 (m, 2H, arom); 7.62 (m, 2H, arom), 7.79 (s, 1H, CH methylidene). ^13^C NMR (DMSO-*d*_6_): δ 25.8, 25.9, 42.5, 67.5, 68.2 (CH_2_); 115.0, 116.3, 121.0, 126.1, 129.9, 130.1, 159.1, 161.5 (CH arom, Cq arom); 119.6 (C-5); 133.6 (CH methylidene); 167.3, 167.4 (C=O); 193.6 (C=S). Anal. (C_22_H_21_NO_5_S_2_) calcd: C 59.58; H 4.77; N 3.16; found: C 59.40; H 4.62; N 3.27.

##### 2-[(5Z)-5-{[3-(4-Phenoxybutoxy)phenyl]methylidene}-4-oxo-2-thioxothiazolidin-3-yl]acetic Acid (**1g**)

Yield 62%; yellow solid; mp 215–218 °C; ^1^H NMR (DMSO-*d*_6_): δ 1.89–1.90 (m, 4H, 2 CH_2_); 4.03 (t *J* = 5.9 Hz, 2H, OCH_2_); 4.11 (t *J* = 5.85 Hz, 2H, OCH_2_); 4.74 (s, 2H, NCH_2_); 6.90–6.94 (m, 3H, arom); 7.12 (dd *J* = 8.45 and 2.3 Hz, 1H, arom); 7.21–7.22 (m, 2H, arom); 7.26–7.29 (m, 2H, arom), 7.47 (dd *J* = 8.25 Hz, 1H, arom), 7.86 (s, 1H, CH methylidene). ^13^C NMR (DMSO-*d*_6_): δ 25.9, 26.0, 45.6, 67.5, 68.1 (CH_2_); 115.0, 116.9, 121.0, 122.7, 123.1, 130.0, 131.2, 134.7, 159.2, 159.7 (CH arom, Cq arom); 118.3 (C-5); 134.5 (CH methylidene); 166.8, 167.8 (C=O); 193.8 (C=S). Anal. (C_22_H_21_NO_5_S_2_) calcd: C 59.58; H 4.77; N 3.16; found: C 59.61; H 4.85; N 3.08.

##### 3-[(5Z)-5-({4-[2-(4-Methoxyphenyl)ethoxy]phenyl}methylidene)-4-oxo-2-thioxothiazolidin-3-yl]propanoic Acid (**2a**)

Yield 38%; yellow solid; mp 202–205 °C; ^1^H NMR (DMSO-*d*_6_): δ 2.62 (t *J* = 7.85 Hz, 2H, CH_2_); 2.99 (t *J* = 6.8 Hz, 2H, CH_2_); 3.72 (s, 3H, OCH_3_); 4.20–4.23 (m, 4H, 2 CH_2_); 6.87 (m, 2H, arom); 7.11 (m, 2H, arom); 7.23 (m, 2H, arom); 7.59 (m, 2H, arom), 7.77 (s, 1H, CH methylidene), 12.49 (br s, 1H, COOH). ^13^C NMR (DMSO-*d*_6_): δ 31.6, 34.6, 40.7, 69.5 (CH_2_); 55.7 (OCH_3_); 114.5, 116.4, 126.2, 130.6, 130.7, 134.0, 158.6, 161.5 (CH arom, Cq arom); 119.7 (C-5); 133.7 (CH methylidene); 167.5, 172.5 (C=O); 193.8 (C=S). Anal. (C_22_H_21_NO_5_S_2_) calcd: C 59.58; H 4.77; N 3.16; found: C 59.74; H 4.70; N 3.00.

##### 3-[(5Z)-5-({3-[2-(4-Methoxyphenyl)ethoxy]phenyl}methylidene)-4-oxo-2-thioxothiazolidin-3-yl]propanoic Acid (**2b**)

Yield 47%; orange solid; mp 132–135 °C; ^1^H NMR (DMSO-*d*_6_): δ 2.63 (t *J* = 7.8 Hz, 2H, CH_2_); 2.99 (t *J* = 6.8 Hz, 2H, CH_2_); 3.69 (s, 3H, OCH_3_); 4.14–4.21 (m, 4H, 2 CH_2_); 6.84 (m, 2H, arom); 7.03 (dd *J* = 8.3 and 2.45 Hz, 1H, arom); 7.08–7.12 (m, 2H, arom); 7.21 (m, 2H, arom); 7.41 (dd *J* = 8 Hz, 1H, arom); 7.69 (s, 1H, CH methylidene); 12.53 (br s, 1H, COOH). ^13^C NMR (DMSO-*d*_6_): δ 31.7, 34.8, 40.9, 69.6 (CH_2_); 55.9 (OCH_3_); 114.7, 117.0, 123.5, 123.6, 130.9, 131.0, 131.7, 135.2, 158.8, 159.8 (CH arom, Cq arom); 118.5 (C-5); 133.9 (CH methylidene); 167.6, 172.8 (C=O); 194.1 (C=S). Anal. (C_22_H_21_NO_5_S_2_) calcd: C 59.58; H 4.77; N 3.16; found: C 59.41; H 4.90; N 3.34.

##### 3-[(5Z)-5-({4-[2-(3-Methoxyphenyl)ethoxy]phenyl}methylidene)-4-oxo-2-thioxothiazolidin-3-yl]propanoic Acid (**2c**)

Yield 56%; yellow solid; m.p. 169–172 °C; ^1^H NMR (DMSO-*d*_6_): δ 2.61 (t *J* = 7.85 Hz, 2H, CH_2_); 3.00 (t *J* = 6.7 Hz, 2H, CH_2_COOH); 3.71 (s, 3H, OCH_3_); 4.21 (t *J* = 7.85 Hz, 2H, OCH_2_); 4.26 (t *J* = 6.7 Hz, 2H, NCH_2_); 6.77 (m, 1H, arom); 6.86–6.88 (m, 2H, arom); 7.09 (m, 2H, arom); 7.20 (dd *J* = 8 Hz, 1H, arom); 7.55 (m, 2H, arom); 7.71 (s, 1H, CH methylidene); 12.51 (br s, 1H, COOH). ^13^C NMR (DMSO-*d*_6_): δ 31.7, 35.6, 40.8, 69.3 (CH_2_); 55.8 (OCH_3_); 112.7, 115.5, 116.5, 122.1, 126.3, 130.3, 133.8, 140.6, 160.1, 161.6 (CH arom, Cq arom); 119.8 (C-5); 134.1 (CH methylidene); 167.7, 172.7 (C=O); 194.0 (C=S). Anal. (C_22_H_21_NO_5_S_2_) calcd: C 59.58; H 4.77; N 3.16; found: C 59.44; H 4.89; N 3.14.

##### 3-[(5Z)-5-({4-[2-(3,4-Dimethoxyphenyl)ethoxy]phenyl}methylidene)-4-oxo-2-thioxothiazolidin-3-yl]propanoic Acid (**2d**)

Yield 30%; yellow solid; mp 204–207 °C; ^1^H NMR (DMSO-*d*_6_): δ 2.61 (t *J* = 7.5 Hz, 2H, CH_2_COOH); 2.97 (t *J* = 6.8 Hz, 2H, CH_2_); 3.70, 3.73 (2s, 6H, 2 OCH_3_); 4.20–4.26 (m, 4H, NCH_2_ and OCH_2_); 6.81 (dd *J* = 8.15 and 1.7 Hz, 1H, arom); 6.86 (d *J* = 8.15 Hz, 1H, arom); 6.92 (d *J* = 1.7 Hz, 1H, arom); 7.10 (m, 2H, arom); 7.57 (m, 2H, arom); 7.74 (s, 1H, CH methylidene). ^13^C NMR (DMSO-*d*_6_): δ 31.6, 35.1, 40.6, 69.6 (CH_2_); 56.2, 56.3 (OCH_3_); 112.6, 113.6, 116.4, 121.6, 126.2, 131.2, 133.8, 148.2, 149.4, 161.6 (CH arom, Cq arom); 119.7 (C-5); 134.0 (CH methylidene); 167.5, 172.6 (C=O); 193.9 (C=S). Anal. (C_23_H_23_NO_6_S_2_) calcd: C 58.33; H 4.90; N 2.96; found: C 59.45; H 5.02; N 3.04.

##### 3-[(5Z)-5-({4-[3-(4-Methoxyphenyl)propoxy]phenyl}methylidene)-4-oxo-2-thioxothiazolidin-3-yl]propanoic Acid (**2e**)

Yield 34%; yellow solid; mp 178–181 °C; ^1^H NMR (DMSO-*d*_6_): δ 1.99 (m, 2H, CH_2_); 2.62 (t *J* = 7.85 Hz 2H, CH_2_COOH); 2.66 (t *J* = 7.95 Hz, 2H, CH_2_C_6_H_5_); 3.70 (s, 3H, OCH_3_); 4.02 (t *J* = 6.35 Hz, 2H, OCH_2_); 4.22 (t *J* = 7.85 Hz, 2H, NCH_2_); 6.83 (m, 2H, arom); 7.09 (m, 2H, arom); 7.12 (m, 2H, arom); 7.57 (m, 2H, arom); 7.74 (s, 1H, CH methylidene). ^13^C NMR (DMSO-*d*_6_): δ 31.1, 31.2, 31.5, 40.6, 67.9 (CH_2_); 55.7 (OCH_3_); 114.5, 116.3, 126.1, 130.0, 133.6, 133.7, 158.2, 161.7 (CH arom, Cq arom); 119.6 (C-5); 134.0 (CH methylidene); 167.5, 172.5 (C=O); 193.8 (C=S). Anal. (C_23_H_23_NO_5_S_2_) calcd: C 60.38; H 5.07; N 3.06; found: C 60.55; H 5.18; N 2.95.

##### 3-[(5Z)-5-{[4-(4-Phenoxybutoxy)phenyl]methylidene}-4-oxo-2-thioxothiazolidin-3-yl]propanoic Acid (**2f**)

Yield 35%; yellow solid; mp 167–170 °C; ^1^H NMR (DMSO-*d*_6_): δ 1.89–1.92 (m, 4H, 2 CH_2_); 2.64 (t *J* = 7.4 Hz, 2H, CH_2_COOH); 4.03 (t *J* = 5.95 Hz, 2H, OCH_2_); 4.13 (t *J* = 5.8 Hz, 2H, OCH_2_); 4.23 (t *J* = 7.4 Hz, 2H, NCH_2_); 6.92–6.94 (m, 3H, arom); 7.11 (m, 2H, arom); 7.27–7.30 (m, 2H, arom); 7.62 (m, 2H, arom), 7.79 (s, 1H, CH methylidene), 12.42 (br s, 1H, COOH). ^13^C NMR (DMSO-*d*_6_): δ 21.6, 25.8, 25.9, 31.4, 67.5, 68.2 (CH_2_); 115.0, 116.2, 121.0, 126.0, 130.0, 133.5, 159.1, 161.5 (CH arom, Cq arom); 119.5 (C-5); 133.8 (CH methylidene); 167.3, 172.3 (C=O); 193.6 (C=S). Anal. (C_23_H_23_NO_5_S_2_) calcd: C 60.38; H 5.07; N 3.06; found: C 60.34; H 4.96; N 3.14.

##### 3-[(5Z)-5-{[3-(4-Phenoxybutoxy)phenyl]methylidene}-4-oxo-2-thioxothiazolidin-3-yl]propanoic Acid (**2g**)

Yield 63%; yellow solid; mp 163–165 °C; ^1^H NMR (DMSO-*d*_6_): δ 1.89 (m, 4H, 2 CH_2_); 2.64 (t *J* = 7.4 Hz, 2H, CH_2_COOH); 4.03 (t *J* = 5.4 Hz, 2H, OCH_2_); 4.10 (t *J* = 5.5 Hz, 2H, OCH_2_); 4.23 (t *J* = 7.4 Hz, 2H, NCH_2_); 6.90–6.94 (m, 3H, arom); 7.09 (m, 1H, arom); 7.18 (m, 2H, arom); 7.25–7.27 (m, 2H, arom); 7.46 (m, 1H, arom); 7.77 (s, 1H, CH methylidene), 12.53 (br s, 1H, COOH). ^13^C NMR (DMSO-*d*_6_): δ 25.9, 26.0, 31.4, 40.7, 67.5, 68.0 (CH_2_); 115.0, 116.7, 120.9, 122.9, 123.2, 129.9, 130.1, 131.2, 134.8, 159.3 (CH arom, Cq arom); 118.1 (C-5); 133.4 (CH methylidene); 167.2, 172.3 (C=O); 193.8 (C=S). Anal. (C_23_H_23_NO_5_S_2_) calcd: C 60.38; H 5.07; N 3.06; found: C 60.49; H 5.11; N 3.09.

### 3.2. Molecular Docking

X-ray crystal structure selection and preparation of the AKR1B1-idose complex (PDB-ID: 3V36) [27] and PTP1B (PDB-ID: 1Q6T) [29] were performed as described in our previous studies [9,10,11]. Molecular docking studies with GOLD (version 2020) [40] were carried out in the catalytic sites of PTP1B and AKR1B1, in the AKR1B1-idose complex, and in the allosteric binding pocket of PTP1B, as we previously reported [9,10,11]. A total of 25 docking poses were generated with default settings using the CHEMPLP scoring function [41]. The docking poses were minimized in LigandScout 4.4.3 using the MMFF94 force field [42]. The 3D pharmacophores were generated using LigandScout 4.4.3, and 3D depictions of protein–ligand complexes were created in MOE version 2022.2 (Chemical Computing Group, Montreal, QC, Canada).

### 3.3. In Silico ADME and Toxicity Predictions

The in silico ADMET evaluation for selected compounds **1g**, **2e**, **2f**, and **2g** was performed by using the SwissADME, PreADMET, and PhaKinPro web tools (available at www.swissadme.ch/index.php, https://preadmet.webservice.bmdrc.org, and https://phakinpro.mml.unc.edu, respectively; accessed on 21–24 January 2025), which incorporate predictive models for physico-chemical properties, drug-likeness, pharmacokinetics, and toxicity [30,31,32,33,34,37].

### 3.4. Assays, Expression, and Purification of AKR1B1

The activity of AKR1B1 was determined by following the decrease in absorbance at 340 nm due to the NADPH oxidation. The standard assay mixture contained 0.25 M sodium phosphate buffer, pH 6.8, 0.5 mM EDTA, 0.18 mM NADPH, 2.4 M ammonium sulphate, and 4.7 mM D,L-glyceraldehyde. One unit of enzyme activity refers to the amount of enzyme that catalyzes the conversion of 1 µmol of substrate under standard conditions. The expression and purification of human recombinant AKR1B1 was performed as previously described [43]; the purified enzyme has a specific activity of 5 U/mg.

#### 3.4.1. Inhibition Studies on AKR1B1

IC_50_ (concentration of compound necessary to determine 50% inhibition of enzyme activity) values were determined in the assay conditions described above, in the presence of 2 mM L-idose as substrate. In each assay, 10 mU of purified AKR1B1 (corresponding to 83 nM in the assay mixture, calculated based on AKR1B1 molecular weight of 34 Kda) were used. All compounds were dissolved in DMSO. Since this solvent exerts an inhibitory effect on AKR1B1 [43], its concentration in all assays was kept constant at 0.5% (*v*/*v*). For each compound, at least five different concentrations of inhibitor, each tested at least in triplicate, were analyzed. IC_50_ values were determined by nonlinear regression analysis using Prism GraphPad 9.5.

#### 3.4.2. Evaluation of AKR1B1 Inhibition Mechanisms

Reaction rates were measured using at least five different L-idose concentrations in the absence and presence of at least four different inhibitor concentrations. ^app^*K_M_* and ^app^*V_max_* were determined by nonlinear regression analysis applying the model “enzyme kinetic velocity vs. [substrate]/Michaelis–Menten equation” using Prism GraphPad 9.5. The inhibition constants *K_i_* (apparent dissociation constant of the EI complex) and *K_i_*′ (apparent dissociation constant of the ESI complex) were determined from secondary plots of 1/^app^*V_max_* and ^app^*K_M_*/^app^*V_max_* as a function of inhibitor concentration, respectively. In the case of tight-binding inhibitors (i.e., IC_50_ values of the same order of magnitude as [E]), the residual activity measurements obtained at each substrate concentration were fitted by nonlinear regression analysis to the Morrison equation [44] for *^app^K_i_* evaluation, applying the model enzyme kinetics-inhibition/tight inhibition Morrison equation using Prism GraphPad 9.5. *K_i_* and *K_i_*′ values were obtained from the *^app^K_i_* measured at different substrate concentrations according to the following equation:

(1) Kiapp = S+KMKMKi+SKi′

relative to a general case of a tight-binding non-competitive inhibition model. The K_M_ value for L-idose used in Equation (1) was 4.0 mM [43]. The reversibility of the inhibitory action was evaluated by measuring the AKR1B1 activity after extensive dialysis (Amicon Ultrafiltration membrane (Sigma-Aldrich, St. Louis, MO, USA), 10 KDa cut off) of an enzyme sample (10 mU) previously treated with an inhibitor concentration able to reduce the enzyme activity to at least 75% of the control value. The recovered activity was compared to that measured for an AKR1B1 sample subjected to the same treatment in the absence of an inhibitor.

### 3.5. Assays, Expression, and Purification of PTP1B

The recombinant human PTP1B was obtained as previously described [11]. Briefly, the sequence codifying PTP1B (1-302 aa) was cloned in bacterial expression vector pNic28 in frame with a poly-His (6xHis) sequence, which was then used to transform the *E. coli* BL21 bacterial strain. The bacteria were grown and then incubated with IPTG (50 µg/L) to induce the expression of a fusion protein. The recombinant PTP1B was purified from bacterial lysate by affinity chromatography using a column packed with a Ni-NTA Agarose resin (Termo Fischer Scientific, Waltham, MA, USA). The fractions containing PTP1B were collected, concentrated by using centrifuge concentrators (Millipore, Burlington, MA, USA). After, the enzyme preparation was loaded on a Superdex G-75 column (Cytiva, Marlborough, MA, USA) and eluted in 50 mM Tris-HCl buffer, containing 150 mM NaCl and 0.5 mM mercaptohetanol. The purity of protein preparations was analyzed by SDS–PAGE according to Laemmli [45]. The solutions containing the proteins were aliquoted in 500 µL fractions and stored at −80 °C.

#### 3.5.1. Inhibition Studies on PTP1B

To determine the activity of PTP1B, enzymatic assays were carried out using the synthetic substrate pNPP, which was dissolved in 0.075 M β,β-dimethylglutarate buffer (pH 7.0). Each assay was initiated by adding an aliquot of the enzyme to the assay solution and then stopped after an appropriate time using a NaOH solution. The amount of pNP released was determined spectrophotometrically by reading each sample at 400 nm [46].

#### 3.5.2. Evaluation of PTP1B Inhibition Mechanisms

Detailed kinetic analyses were carried out to evaluate the mechanism of compounds **1g**, **2e**–**g** on PTP1B. To evaluate if compounds acted as reversible or irreversible inhibitors, a dilution assay was carried out. Briefly, an aliquot of PTP1B was incubated for 60 min at 37 °C in the presence of a saturating amount of each PTP1B inhibitor. Then, the residual activity of the enzyme was determined by diluting an aliquot of the enzyme–inhibitor solution in 2 mL of the assay buffer containing 2.5 mM of substrate and measuring the amount of pNP released using a spectrophotometer.

The mechanism of action of compound **2e** was determined by measuring the dependence of K_M_ and V_max_ from the inhibitor concentration. Data obtained were fitted using both the Michaelis–Menten and Lineweaver–Burk equations. The K_i_ value was calculated by fitting experimental data with the appropriate equations.

#### 3.5.3. Continuous PTP1B Inhibition Assays

Continuous assay tests were carried out with a final volume of 1.5 mL. Each assay sample contained a buffer of pH 7.0 and substrate (5 mM pNPP). The reactions were started by mixing the enzyme into a solution. In situ pNP release was monitored by reading the absorbance of samples at 405 nm for 40 min at 25 °C. The absorbance values were converted into the pNP concentration using the ε_mM_ value of pNPP (18 mM^−1^cm^−1^). Data were then fitted to Equation (S1) (see Supplementary Materials) to determine the apparent first-order rate constant for establishment of the enzyme–inhibitor equilibrium (k_a_). Further continuous tests were carried out using a fixed inhibitor concentration and increasing substrate concentrations to determine the competitive or non-competitive nature of the inhibitors. The K_i_ and K′_i_ values were determined using an appropriate equation derived from kinetic models.

### 3.6. Cell Cultures and Assays

Human lens epithelial B3 line (HLE) cells were purchased from American Type Culture Collection (Rockville, MD, USA) and cultured at 37 °C in a humidified atmosphere, in the presence of 5% CO_2_, in an Eagle’s modified minimum essential medium (MEM) containing 5.5 mM D-glucose, supplemented with 20% (*v*/*v*) FBS, 50 mU/mL penicillin/streptomycin, 2 mM glutamine and 100 μg/mL hygromycin. Cells were grown in the indicated medium until they were 70% confluent. Before experiments, cells were incubated for 24 h in MEM containing 0.5% FBS, 50 μg/mL gentamicin, 2 mM glutamine, and 100 μg/mL hygromycin. Cells at passages 15–20 (30–40 residual doublings), plated at a density of 20,000 cells/cm^2^, were used for experiments.

The human Muller glial line (MIO-M1) cells were a gift from Prof. Massimo Dal Monte (University of Pisa). MIO-M1 cells were cultured at 37 °C in the presence of 5% CO_2_, in a humidified atmosphere, in a Dulbecco’s Modified Eagle Medium (DMEM) containing 5.5 mM D-glucose supplemented with 20% (*v*/*v*) FBS, 50 mU/mL penicillin/streptomycin, 2 mM glutamine, and 1% non-essential amino acid solution. Cells were grown in the indicated medium until they were 80% confluent. Before experiments, cells were incubated for 24 h in DMEM containing 0.5% FBS, 50 mU/mL penicillin/streptomycin, 2 mM glutamine, and 1% non-essential amino acid. Cells at passages 14–19, plated at a density of 18,000 cells/cm^2^, were used for experiments.

C2C12 and HepG2 cells were purchased by ATCC and grown at 37 °C in 5% CO_2_ and a humidified atmosphere in the presence of high glucose DMEM (Euroclone, Pero, Italy) supplemented with 10% FBS (Euroclone), 50 mU/mL penicillin/streptomycin, and 2 mM glutamine (Merck, Darmstadt, Germany).

#### 3.6.1. Measurements of Cell Viability

HLE, MIO-M1, C2C12, and HepG2 cells’ viability was assessed after 48 h incubation at 37 °C in the proper culture medium (see above), supplemented with 0.05% DMSO alone or in the presence of the selected compounds or the reference drug. Then, cells were washed with phosphate-buffered solution (PBS, Merck Millipore, Darmstadt, Germany) and incubated for 1 h with 0.5 mg/mL of thiazolyl blue tetrazolium bromide (MTT, Merck Millipore) dissolved in a phosphate-buffered solution pH 7.4. The formazane crystals were then dissolved by the addition of 0.04 N HCl in isopropanol, and the absorbance of the solution at 563 nm was measured using a microplate reader [47].

#### 3.6.2. Assessment of Insulin Signalling via SDS-PAGE and Western Blot Analysis

To evaluate the impact of tested compounds on the insulin signalling pathway, we analyzed the phosphorylation status of the kinase Akt using the Western blot method. Briefly, C2C12 cells were seeded in 35 mm dishes and grown until they reached 80% confluence. The cells were then washed with PBS and incubated in starvation medium for 24 h. After starvation, the cells were stimulated with 10 nM insulin or with 10 μM compound for 30 min and subsequently lysed in 150 μL of 1× Laemmli sample buffer. Before lysis, the cells were washed with cold (4 °C) PBS. Protein lysates were collected, transferred to 1.5 mL conical Eppendorf tubes, and boiled for 5 min to ensure complete protein denaturation. The boiled samples were placed on ice for 15 min and then stored at −20 °C. The following day, protein samples were analyzed by SDS-PAGE, loading 10 μL of each sample onto 4–20% Mini-PROTEAN^®^ TGX™ Precast Protein Gels (Bio-Rad, Hercules, CA, USA). After approximately 1 h of electrophoresis, the run was stopped, the gels were removed, and the separated proteins were transferred onto PVDF membranes (Immun-Blot PVDF membrane, Bio-Rad) using a Trans-Blot Turbo system (Bio-Rad). Following transfer, the membranes were incubated for 30 min with a blocking solution consisting of 5% BSA in PBS containing 0.1% Tween 20.

To evaluate both the phosphorylation status and total levels of Akt, the membranes were incubated overnight with primary antibodies recognizing phosphorylated Akt (9271S, Phospho-Akt (Ser473) Antibody, Cell Signalling Technology, Danvers, MA, USA) and total Akt (9272S, Akt Antibody, Cell Signalling Technology). The next day, the membranes were extensively washed with PBS containing 0.05% Tween 20 to remove unbound antibodies and then incubated for 1 h at 4 °C with HRP-conjugated secondary antibodies.

After incubation with secondary antibodies, the membranes were washed again with PBS containing 0.05% Tween 20 and analyzed using a luminometer (Imager 600 Luminescence Image Analyzer, GE Amersham, Little Chalfont, UK). Before detection, the PVDF membranes were incubated with 2 mL of ECL START Western blotting Detection Reagent. The intensity of each band was determined using the Amersham Imager 600 Analysis Software. Each test was carried out in triplicate. The data obtained were normalized with respect to the control samples.

#### 3.6.3. Determination of Sorbitol Content

HLE and MIO-M1 cells were washed with PBS, supplemented with 1 mM PMSF, harvested, and lysed by means of three freezing/thawing cycles. The supernatant obtained after centrifugation at 10,000× *g* at 4 °C per 30 min (crude cell extract) was supplemented with 4 M ice-cold perchloric acid. Samples were then neutralized using 5 M KOH, and the sorbitol content was measured as described [48]. Protein content was measured in the crude extracts according to Bradford [49].

#### 3.6.4. Glucose Uptake Assay

C2C12 cells seeded in 24-well plates were serum-starved for 24 h and then stimulated with 10 nM insulin, and/or with 10 μM of compounds **1g**, **2e**–**g** for 30 min. After this time, the starvation medium was removed, the cells were washed with PBS, and they were then incubated with 1 mL starvation medium containing 40 μM of 2-NBDG. After 3 h, the medium was removed, and the cells were washed with fresh PBS and trypsinized. Detached cells were collected by centrifugation at 1000 rpm, washed with PBS, and analyzed using a FACS Canto II flow cytometer (BD Biosciences, Franklin Lakes, NJ, USA). All tests were carried out in quadruplicate.

#### 3.6.5. Evaluation of the Mitochondrial Membrane Potential

C2C12 cells were seeded on 6-well plates (50,000 cells for each well) and incubated at 37 °C. Then, cells were treated for 30 min with a growth medium containing 1 μM FCCP or 25 μM of compounds **1g**, **2e**–**g**. After incubation, cells were tripsinized, collected, and analyzed using a flow cytometer (BD FACS Canto II Analyzer, BD Bioscience) and the FlowJo software (v.10). For each test, 10,000 events were acquired.

#### 3.6.6. Measurement of Lipid Accumulation

Oil Red O stock solution was prepared by dissolving 0.2 g of dye in 40 mL of 2-propanol (0.5% *w*/*v*). The working solution was obtained by diluting the stock solution in distilled water (2:3 Oil Red/water). Before use, the working solution was filtered through 0.22 μm syringe filters and stored at room temperature. HepG2 cells (70,000 cells per well) were seeded in 24-well plates and incubated at 37 °C in a 5% CO_2_ atmosphere. The following day, the cells were washed with PBS and incubated with 2 mL of fresh starvation medium, containing or not containing 0.4 mM oleic acid, then stored again at 37 °C. After 24 h, the medium containing oleic acid was removed, and the cells were washed with PBS. Then, 2 mL of starvation medium containing 25 μM of compound **1g** or **2e**–**g** was added to the plate, which was subsequently incubated at 37 °C for an additional 72 h. After this period, the cells were washed with PBS and then analyzed to evaluate the lipid content using the Oil Red O staining method. For this purpose, the medium was removed and replaced with 250 μL of 60% 2-propanol solution. After a 5 min incubation, the isopropanol was withdrawn and replaced with 500 μL of Oil Red O working solution. After 30 min, the Oil Red O solution was removed, and the plates were washed five times with distilled water. Then, 500 μL of 100% 2-propanol was added to each well to aid in the solubilization of the dye bound to intracellular lipids. After a 10 min incubation, the absorbance of the solutions was determined using the iMark™ microplate reader (Bio-Rad), measuring the absorbance at 510 nm. Each experiment was carried out in quadruplicate. After reading, each plate was washed with distilled water to completely remove the Oil Red O dye. Next, 300 μL of Bradford reagent (Sigma Aldrich) was added to each well. The plates were incubated at room temperature for 10 min, then washed with water. The plates were scanned, and the wells were analyzed with ImageJ 1.54g to quantify the protein content in each well. Finally, the data from the Oil Red O test were normalized to the protein content. All data were normalized relative to the control experiment.

#### 3.6.7. Statistical Analysis of Data

When required, statistical analysis was performed by using the Unpaired Student’s *t*-test or one-way Anova with the Dunnet post hoc test, as specifically detailed in each figure legend.

## 4. Conclusions

Continuing an ongoing search for potential multitarget antidiabetic agents, a new series of (5-arylidene-4-oxothiazolidin-3-yl)alkanoic acids (**1a**–**g**, **2a**–**g**) were synthesized and evaluated, with the main aim of investigating the relationships between their activity and a more extended 5-arylidene moiety, which was modified by means of the introduction of methoxy substituents on the distal phenyl ring and/or elongation of the linker chain between the two aromatic rings. Regarding the AKR1B1/PTP1B inhibitory activity, the newly synthesized compounds **1a**–**g** and **2a**–**g** showed excellent inhibitory activity toward AKR1B1, along with less potent effects against PTP1B. Compounds **1g** and **2e**–**g** displayed an appreciable capability to inhibit both target enzymes; however, compared with the previously reported analogues [9,10,11], they did not lead to any substantial improvement in inhibitory potency. Surprisingly, despite their scarce capability to improve insulin signalling in murine myocytes C2C12 by inhibiting PTP1B, compounds **2e**, **2f**, and, to a lesser extent, **2g** markedly stimulated cellular glucose uptake in the same cells; no appreciable differences were observed when the cells were treated with each compound alone or in combination with insulin, clearly indicating an insulin-mimetic activity. It could be inferred that, at the concentration (10 µM) used in the ex vivo assays, which is very close to or lower than the observed IC_50_ values, the effect of PTP1B inhibition on improving cellular sensitivity to insulin may be insignificant or not detectable. Therefore, compounds **2e**–**g** must be capable of stimulating cellular glucose uptake in an insulin-independent manner. Assays performed in C2C12 myocytes and HepG2 liver cells indicated that 3-(5-arylidene-4-oxo-2-thioxothiazolidin-3-yl)propanoic acid derivatives **2e**–**g** can produce an appreciable increase in mitochondrial potential and alter pyruvate metabolism, suggesting that the observed significant increase in both glucose uptake and fatty acid degradation might be related to these mitochondrial activities. Compound **2e** was also able to significantly impair sorbitol accumulation in both human lens epithelial and retinal glial cells, by strongly inhibiting AKR1B1; in the same assay, another potent AKR1B1 inhibitor, acetic acid derivative **1g**, showed excellent activity in lens epithelial cells, reaching activity levels like that of the reference drug sorbinil.

The finding that compounds **2e**–**g** could act as insulin-mimetic agents through insulin-independent mechanisms is significant and, therefore, could motivate further studies. Moreover, it could be worth further investigating the relationships of these mechanisms with certain structural features of 4-thiazolidinone derivatives, since analogous 2,4-thiazolidinediones were shown to act differently on pyruvate metabolism [38]. The substitution pattern of the 5-arylidene moiety might play a central role by affecting these concurrent mechanisms and modulating the multifaceted antidiabetic potential of this class of compounds. Moreover, the impact of combined effects elicited by alterations of pyruvate mitochondrial pathways on such a complex disease as DM has not yet been sufficiently studied, and thus the compounds here reported might provide tools for further investigations.

Finally, it is worth noting that thiazolidine derivatives have often been the object of debate on their suitability as starting hits in the process of drug discovery, especially due to concerns regarding their metabolic and toxicity profiles. However, it was demonstrated that the in vivo behaviour of these derivatives can be effectively modulated by a substitution pattern of the heterocyclic core, since it depends on the entire molecular entity, and, therefore, thiazolidine and other five-membered heterocycle derivatives should not be subject to inappropriate negative bias [50,51]. Interestingly, the insertion of the pharmacophoric polar carboxylic chain on N-3 of the thiazolidinone scaffold in compounds **1a**–**g** and **2a**–**g**, as well as in the analogues that we previously reported [9,10,11,25,26], can be responsible for decreased lipophilicity and increased metabolic stability; therefore, these thiazolidinone derivatives could be less prone to producing potentially toxic metabolites [50]. Overall, our structure–activity relationship studies of (5-arylidene-4-oxothiazolidin-3-yl)alkanoic acid derivatives as inhibitors of AKR1B1 and PTP1B demonstrated that the substitution pattern in both positions 3 and 5 of the thiazolidinone scaffold can deeply influence the inhibitory potency as well as the mechanism of action of these compounds. Compelling evidence indicates that substituted 4-thiazolidinones, obtained by means of appropriately designed derivatization [9,10,11,25,26,50,51], can be attractive compounds in drug design and development, thus encouraging us to further investigate this class of enzyme inhibitors and their multifarious activity profiles.

## Figures and Tables

**Table 1:** In vitro inhibition of human AKR1B1 and PTP1B by compounds **1a**–**g** and **2a**–**g**.

Compd.	AKR1B1 IC_50_ (μM) ^a^	PTP1B IC_50_ (μM) ^a^
**1a**	0.03 ± 0.01	80.5 ± 1.8
**1b**	0.04 ± 0.01	91.4 ± 1.4
**1c**	0.08 ± 0.01	50.8 ± 2.8
**1d**	0.12 ± 0.02	100.0 ± 0.3
**1e**	0.46 ± 0.09	>100
**1f**	0.09 ± 0.01	43.8 ± 1.0
**1g**	0.03 ± 0.01	27.7 ± 1.3
**2a**	0.48 ± 0.13	58.0 ± 6.0
**2b**	3.11 ± 0.73	48.2 ± 4.0
**2c**	3.73 ± 0.63	59.7 ± 4.4
**2d**	6.30 ± 1.65	>100
**2e**	0.69 ± 0.13	12.1 ± 0.2
**2f**	1.18 ± 0.21	23.9 ± 0.7
**2g**	1.89 ± 0.24	24.6 ± 0.7
Sorbinil	0.99 ± 0.13	---
Vanadate	---	0.4 ± 0.01

^a^ Values reported in the table represent IC_50_ ± SE.

**Table 2:** Values of inhibition constants of selected inhibitors.

Compd.	AKR1B1		PTP1B	
	K_i_	K′_i_	K_i_	K′_i_
**1g**	0.03 ± 0.01	0.03 ± 0.01	3.0	n.d
**2e**	n.d.	1.18 ± 0.01	10.8 ± 0.3	29.6 ± 3.4
**2f**	7.75 ± 0.26	2.01 ± 0.01	0.16 ± 0.01	6.9 ± 1.6
**2g**	1.14 ± 0.06	4.43 ± 0.30	0.03 ± 0.01	2.2 ± 1.0

K_i_ and K′_i_ values, expressed in μM, are the mean ± SE; n.d.: not detectable.

**Table 3:** AKR1B1-idose complex protein–ligand interactions with **1g**, **2e**–**g**.

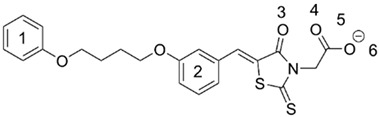		
**1g**—Ligand Moiety	Interaction Type	Protein Residues
Phenyl 1	Hydrophobic	Trp20, Val47
Phenyl 2	Hydrophobic	Trp219, Leu301
Oxygen 3	Hydrogen bond acceptor	Ala299
Carboxyl 5	Ionic	Arg217, Lys221
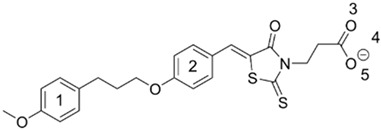		
**2e**—Ligand Moiety	Interaction Type	Protein Residues
Phenyl 1	Hydrophobic	Phe122
Phenyl 2	Hydrophobic	Trp219, Ala299, Leu301
Carboxyl 4	Ionic	Lys221
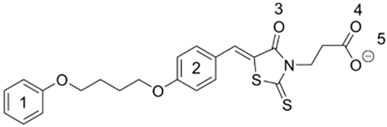		
**2f**—Ligand Moiety	Interaction Type	Protein Residues
Phenyl 1	Hydrophobic	Trp20, Val47
Phenyl 2	Hydrophobic	Trp219, Leu301
Oxygen 3	Hydrogen bond acceptor	Ala299
Carboxyl 6	Ionic	Arg217, Lys221
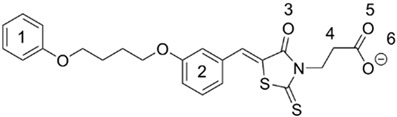		
**2g**—Ligand Moiety	Interaction Type	Protein Residues
Phenyl 1	Hydrophobic	Trp20, Val47
Phenyl 2	Hydrophobic	Trp219, Leu301
Oxygen 3	Hydrogen bond acceptor	Ala299
Carboxyl 6	Ionic	Arg217, Lys221

**Table 4:** AKR1B1 catalytic binding site protein–ligand interactions with **1g**, **2e**–**g**.

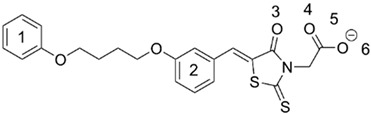		
**1g**—Ligand Moiety	Interaction Type	Protein Residues
Phenyl 1	Hydrophobic	Trp20, Val47, Trp79, Phe122
Phenyl 2	Hydrophobic	Trp219, Ala299, Leu301
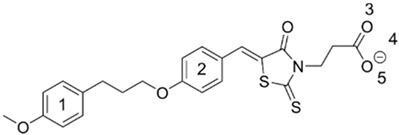		
**2e**—Ligand Moiety	Interaction Type	Protein Residues
Phenyl 1	Hydrophobic	Trp20, Val47, Trp79, Phe122
Phenyl 2	Hydrophobic	Trp219, Leu301
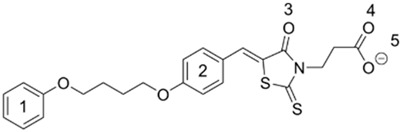		
**2f**—Ligand Moiety	Interaction Type	Protein Residues
Phenyl 1	Hydrophobic	Trp20, Val47, Trp79, Phe122
Phenyl 2	Hydrophobic	Trp219, Ala299, Leu301
Phenyl 2	Aromatic	Trp219
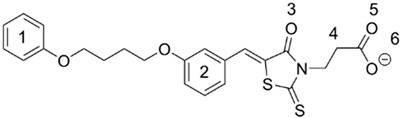		
**2g**—Ligand Moiety	Interaction Type	Protein Residues
Phenyl 1	Hydrophobic	Trp20, Val47, Trp79, Phe122
Phenyl 2	Hydrophobic	Trp219, Ala299, Leu301
Phenyl 2	Aromatic	Trp219
Ethyl 4	Hydrophobic	Leu124

**Table 5:** PTP1B allosteric binding site protein–ligand interactions with **1g**, **2e**–**g**.

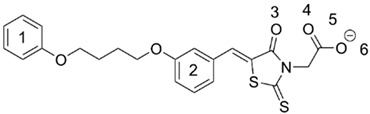		
**1g**—Ligand Moiety	Interaction Type	Protein Residues
Phenyl 1	Hydrophobic	Arg79, Pro206, Pro210
Phenyl 2	Hydrophobic	Lys103
Carboxyl 5	Ionic	Lys103, Arg105
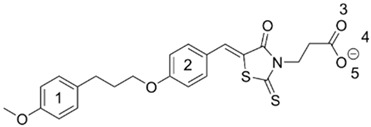		
**2e**—Ligand Moiety	Interaction Type	Protein Residues
Phenyl 1	Hydrophobic	Pro206, Pro210
Phenyl 2	Hydrophobic	Lys103
Carboxyl 4	Ionic	Lys103, Arg105, Arg169
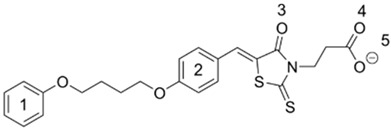		
**2f**—Ligand Moiety	Interaction Type	Protein Residues
Phenyl 1	Hydrophobic	Arg79, Pro206, Pro210
Carboxyl 5	Ionic	Lys103, Arg105, Arg169
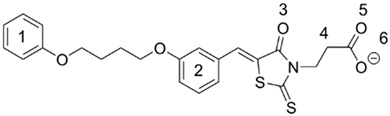		
**2g**—Ligand Moiety	Interaction Type	Protein Residues
Phenyl 1	Hydrophobic	Arg79, Pro206, Pro210
Carboxyl 6	Ionic	Lys103, Arg105, Arg169

**Table 6:** PTP1B catalytic binding site protein–ligand interactions with **1g**, **2e**–**g**.

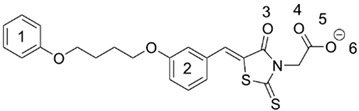		
**1g**—Ligand Moiety	Interaction Type	Protein Residues
Phenyl 1	Hydrophobic	Met254
Phenyl 2	Hydrophobic	Val49
Oxygen 4	Hydrogen bond acceptor	Cys215
Carboxyl 5	Ionic	Arg221
Oxygen 6	Hydrogen bond acceptor	Ser216
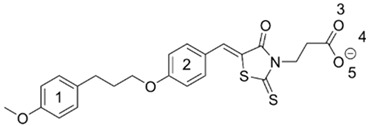		
**2e**—Ligand Moiety	Interaction Type	Protein Residues
Phenyl 1	Hydrophobic	Met254
Phenyl 2	Hydrophobic	Val49
Oxygen 3	Hydrogen bond acceptor	Cys215
Carboxyl 4	Ionic	Arg221
Oxygen 5	Hydrogen bond acceptor	Ser216
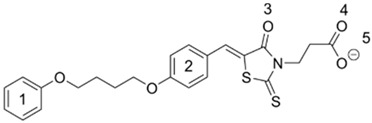		
**2f**—Ligand Moiety	Interaction Type	Protein Residues
Phenyl 1	Hydrophobic	Met254
Oxygen 3	Hydrogen bond acceptor	Phe182
Carboxyl 5	Ionic	Arg221
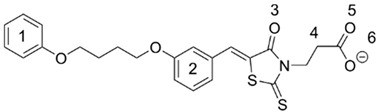		
**2g**—Ligand Moiety	Interaction Type	Protein Residues
Phenyl 1	Hydrophobic	Met254
Phenyl 2	Hydrophobic	Val49
Oxygen 5	Hydrogen bond acceptor	Phe182
Carboxyl 6	Ionic	Arg221

**Table 7:** Values of some calculated molecular descriptors of compounds **1g**, **2e**, **2f**, and **2g** ^a^.

	1g	2e	2f	2g	EPA
MW	443.54	457.56	457.56	457.56	319.40
logP	3.84	4.00	4.07	4.10	2.43
HBD	1	1	1	1	1
HBA	5	5	5	5	3
RB	10	10	11	11	4
TPSA (Å^2^)	133.46	133.46	133.46	133.46	115.00
Lipinski’s rule violation	0	0	0	0	0
Veber’s rule violation	0	0	1	1	0

^a^ SwissADME http://www.swissadme.ch/index.php.

**Table 8:** Predictive ADME profile of compounds **1g**, **2e**, **2f**, and **2g** ^a,b^.

	1g	2e	2f	2g	EPA
Caco2 cell permeable ^a,b^	Yes	Yes	Yes	Yes	Yes
Oral bioavailability ^b^	>0.8 F	0.5–0.8 F	0.5–0.8 F	>0.8 F	<0.5 F
Plasma protein binder ^a,b^	Yes	Weak	Yes	Yes	Yes
Hepatic stability at 60 min ^b^	>50%	>50%	>50%	>50%	>50%
CYP2D6 substrate ^a^	No	No	No	No	No
CYP3A4 substrate ^a^	Weakly	No	Weakly	Weakly	Yes
CYP2C19 inhibition ^a^	No	No	No	No	No
CYP2C9 inhibition ^a^	Yes	No	No	No	No
CYP2D6 inhibition ^a^	No	No	No	No	No
CYP3A4 inhibition ^a^	No	Yes	Yes	No	No

^a^ PreADMET (https://preadmet.webservice.bmdrc.org). ^b^ PhaKinPro (https://phakinpro.mml.unc.edu).

**Table 9:** Predictive toxicity profile of compounds **1g**, **2e**, **2f**, and **2g** ^a^.

	1g	2e	2f	2g	EPA
2-year carcinogenicity in mice	NC	NC	NC	NC	NC
2-year carcinogenicity in rats	NC	NC	NC	NC	NC
Acute algae toxicity	0.0069	0.0060	0.0060	0.0053	0.0280
Acute daphnia toxicity	0.0058	0.0039	0.0041	0.0044	0.0329
Acute fish toxicity	0.00023	0.00015	0.00016	0.00016	0.0027
hERG inhibition risk	Medium	Medium	Medium	Medium	Medium

^a^ PreADMET (https://preadmet.webservice.bmdrc.org); NC = non-carcinogen.

## Data Availability

The original contributions presented in this study are included in the article and Supplementary Materials. Further inquiries can be directed to the corresponding author.

## Supplementary Materials

The following supporting information can be downloaded at: https://www.mdpi.com/article/10.3390/ph18121863/s1, Figures S1–S28: ^1^H NMR and ^13^C NMR spectra of compounds **1a**–**g** and **2a**–**g**; Figures S29–S32: Rate measurements of the AKR1B1 dependent reduction in L-idose in the presence of compounds **2e**, **2f**, **2g** and **1g**; Figure S33: Dilution assay of PTP1B with selected compounds **1g**, **2e**–**g**; Figures S34–S36: Continuous inhibition of PTP1B by compounds **1g**, **2f** and **2g** at pH 7.0 and 25 °C; Figures S37–S39: Continuous inhibition of PTP1B by compound **1g**, **2f** and **2g** in the presence of increasing concentrations of substrate; Figure S40: Dependence of Km and Vmax from concentration of compound **2e**; Figure S41: Dependence of Km from the concentration of compound **2e**; Figure S42: Dependence of Vmax from the concentration of compound **2e**; Figure S43: Lineweaver-Burk plot of compound **2e**; Figure S44: k_a_ secondary plot relative to compound **1g**; Figure S45: v_0_ and k_a_ secondary plots relative to compound **2f**; Figure S46: v_0_ and k_a_ secondary plots relative to compound **2g**; Figure S47: Effect of compounds **1g**, **2e**–**g** on cell viability (C2C12 cells); Figure S48: Lipid accumulation assay; Figure S49: Effect of compounds **1g**, **2e**–**g** on cell viability (MIO-M1 and HLE cells); Scheme S1: Mechanism of action of compound **1g** (competitive slow-binding model); Scheme S2: Mechanism of action of compound **2f**. References in Supplementary Materials can be found in [52,53].
