# Supplementary material for: An Ongoing Search for Multitarget Ligands as Potential Agents for Diabetes Mellitus and Its Long-Term Complications: New Insights into (5-Arylidene-4-oxothiazolidin-3-yl)alkanoic Acid Derivatives
Source: Pharmaceuticals (Basel). 2025 Dec 5;18(12):1863. doi: 10.3390/ph18121863 (PMC12735461; doi:10.3390/ph18121863)
Supplement: Supplementary file 1 [file pharmaceuticals-18-01863-s001.zip › pharmaceuticals-3974972-supplementary.pdf]

SUPPLEMENTARY MATERIAL

An Ongoing Search for Multitarget Ligands as Potential Agents for Diabetes Mellitus and Its Long-Term Complications: New Insights into (5-Arylidene-4-oxothiazolidin-3-yl)alkanoic Acid Derivatives

Rosanna Maccari [1,*], Rosaria Ottanà [1], Valerij Talagayev [2], Roberta Moschini [3], Francesco Balestri [3], Francesca Felice [3], Francesca Iannuccilli [4], Gemma Sardelli [3], Rebecca Sodano [4], Gerhard Wolber [2], Paolo Paoli [4,†] and Antonella Del Corso [3,†]

[1] Department of Chemical, Biological, Pharmaceutical and Environmental Sciences, University of Messina, Viale F. Stagno d'Alcontres 31, 98166 Messina, Italy; rottana@unime.it
[2] Molecular Design Group, Institute of Pharmacy, Freie Universität Berlin, Königin-Luisestr. 2 + 4, 14195 Berlin, Germany; v.talagayev@fu-berlin.de (V.T.); gerhard.wolber@fu-berlin.de (G.W.)
[3] Biochemistry Unit, Department of Biology, University of Pisa, via S. Zeno, 51, 56123 Pisa, Italy; roberta.moschini@unipi.it (R.M.); francesco.balestri@unipi.it (F.B.); francesca.felice@unipi.it (F.F.); gemma.sardelli@phd.unipi.it (G.S.); antonella.delcorso@unipi.it (A.D.C.)
[4] Department of Scienze Biomediche Sperimentali e Cliniche, Sezione di Scienze Biochimiche, University of Firenze, Viale Morgagni 50, 50134 Firenze, Italy; f.iannuccilli@student.unisi.it (F.I.); rebecca.sodano@unifi.it (R.S.); paolo.paoli@unifi.it (P.P.)

* Correspondence: rmaccari@unime.it

† These authors contributed equally to this work.

Figures S1-S28. ^{1}H NMR and ^{13}C NMR spectra of compounds **1a-g** and **2a-g**.

Figures S29-S32. Rate measurements of the AKR1B1 dependent reduction of L-idose in the presence of compounds **2e, 2f, 2g** and **1g**.

Figure S33. Dilution assay of PTP1B with selected compounds **1g**, **2e-g**.

Figures S34-S36. Continuous inhibition of PTP1B by compounds **1g, 2f** and **2g** at pH 7.0 and 25°C.

Figures S37-S39. Continuous inhibition of PTP1B by compound **1g**, **2f** and **2g** in the presence of increasing concentrations of substrate.

Figure S40. Dependence of Km and Vmax from concentration of compound **2e**.

Figure S41. Dependence of Km from the concentration of compound **2e**.

Figure S42. Dependence of Vmax from the concentration of compound **2e**.

Figure S43. Lineweaver-Burk plot of compound **2e**.

Figure S44. k_a secondary plot relative to compound **1g**.

Figure S45. v_0 and k_a secondary plots relative to compound **2f**.

Figure S46. v_0 and k_a secondary plots relative to compound **2g**.

Figure S47. Effect of compounds **1g**, **2e-g** on cell viability (C2C12 cells).

Figure S48. Lipid accumulation assay.

Figure S49. Effect of compounds **1g**, **2e-g** on cell viability (MIO-M1 and HLE cells).

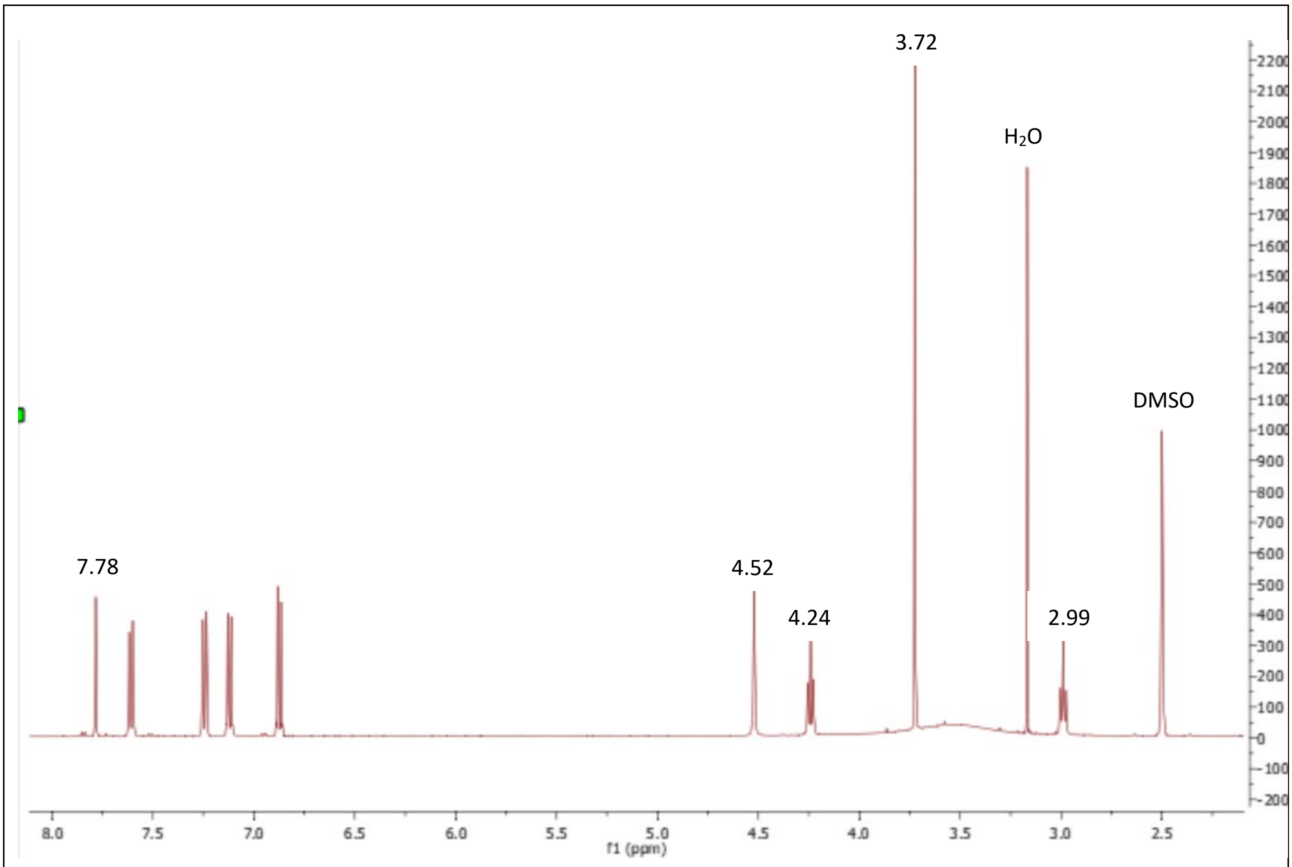

Figure S1. ^{1}H-NMR spectrum of compound **1a** (500 MHz, DMSO-d_6)

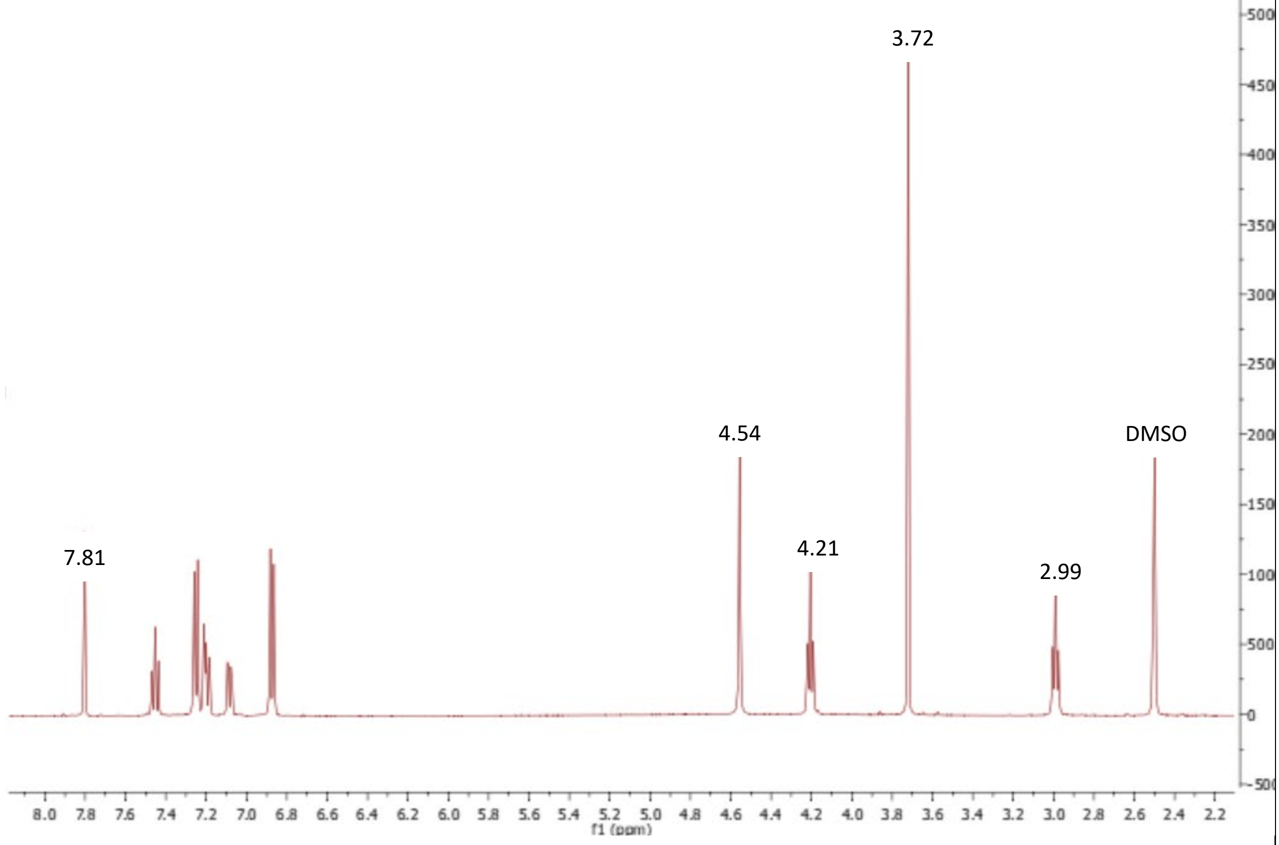

Figure S2. ^{1}H-NMR spectrum of compound **1b** (500 MHz, DMSO-d_6)

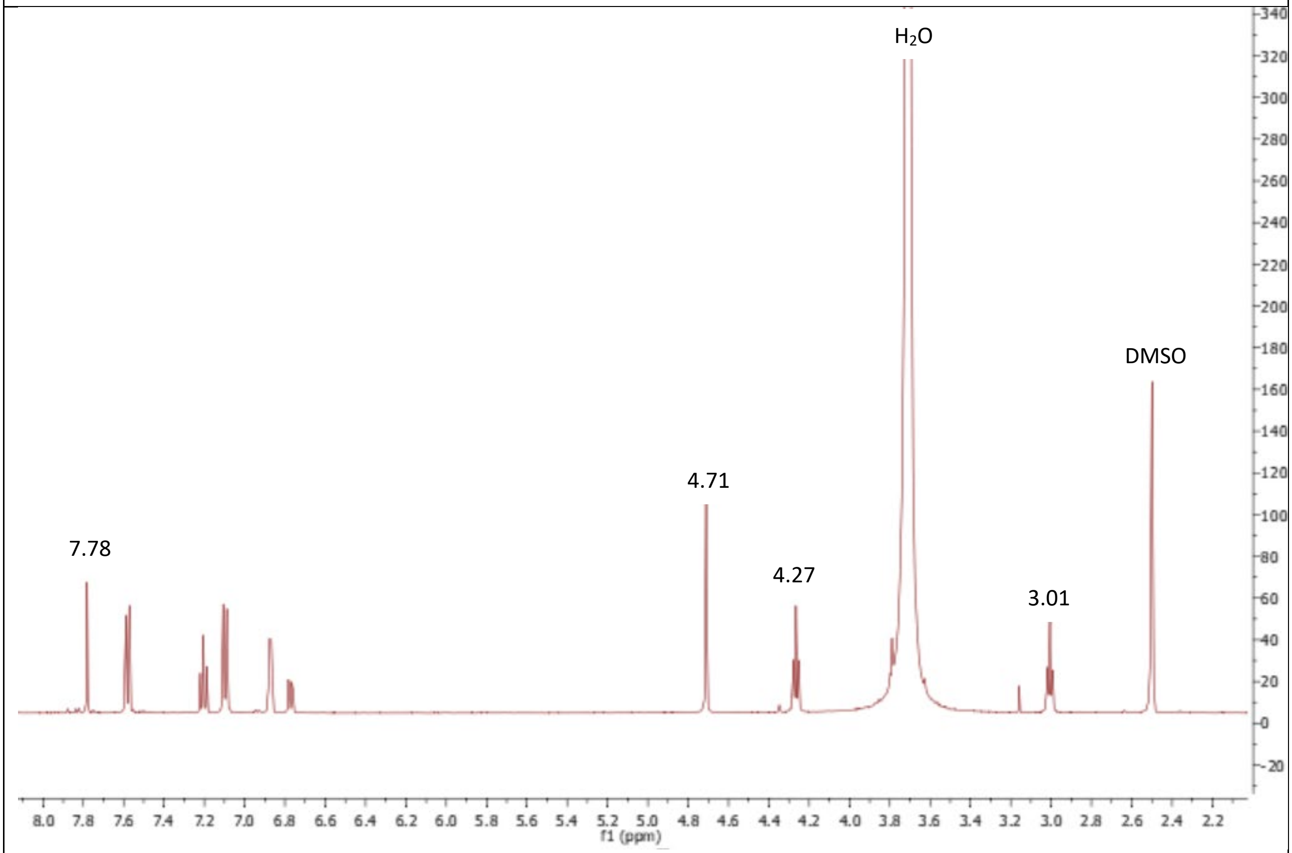

Figure S3. ^{1}H-NMR spectrum of compound **1c** (500 MHz, DMSO-d_6)

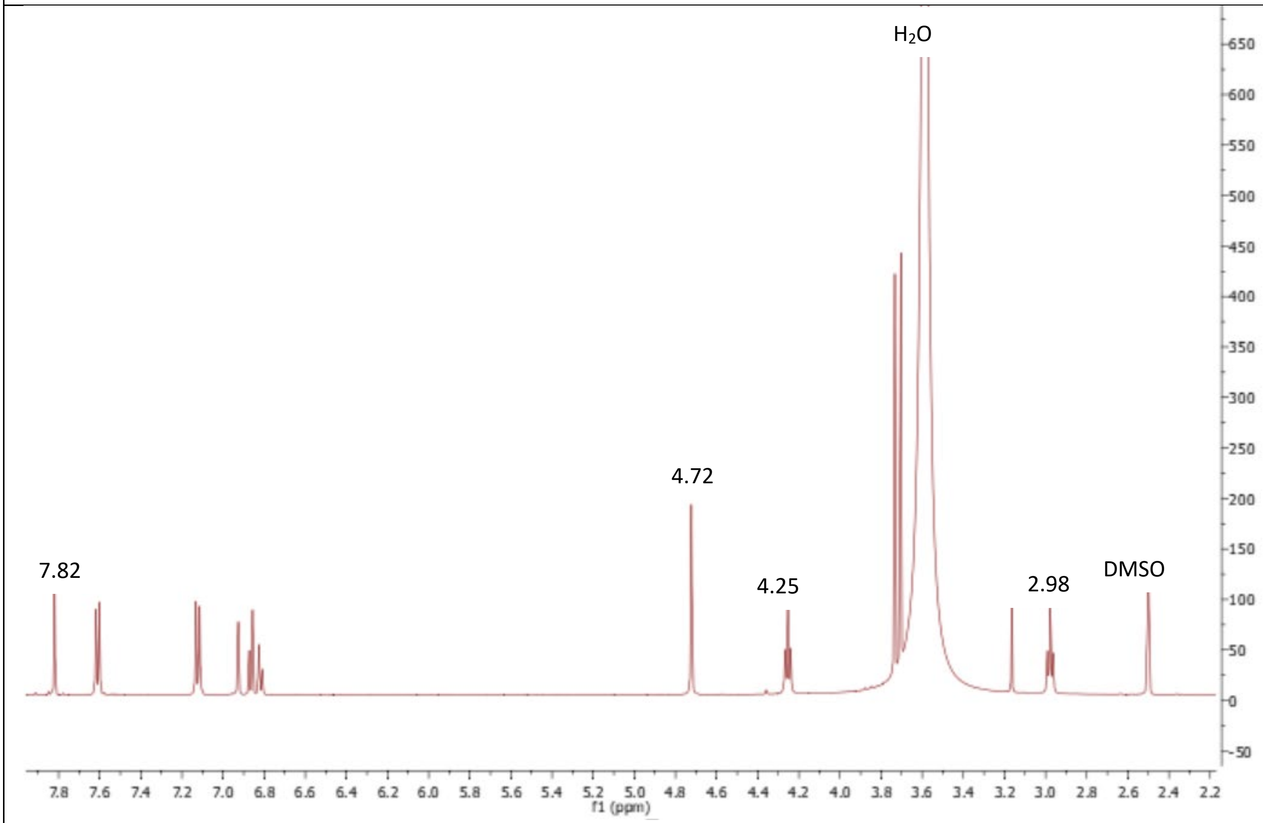

Figure S4. ^{1}H-NMR spectrum of compound **1d** (500 MHz, DMSO-d_6)

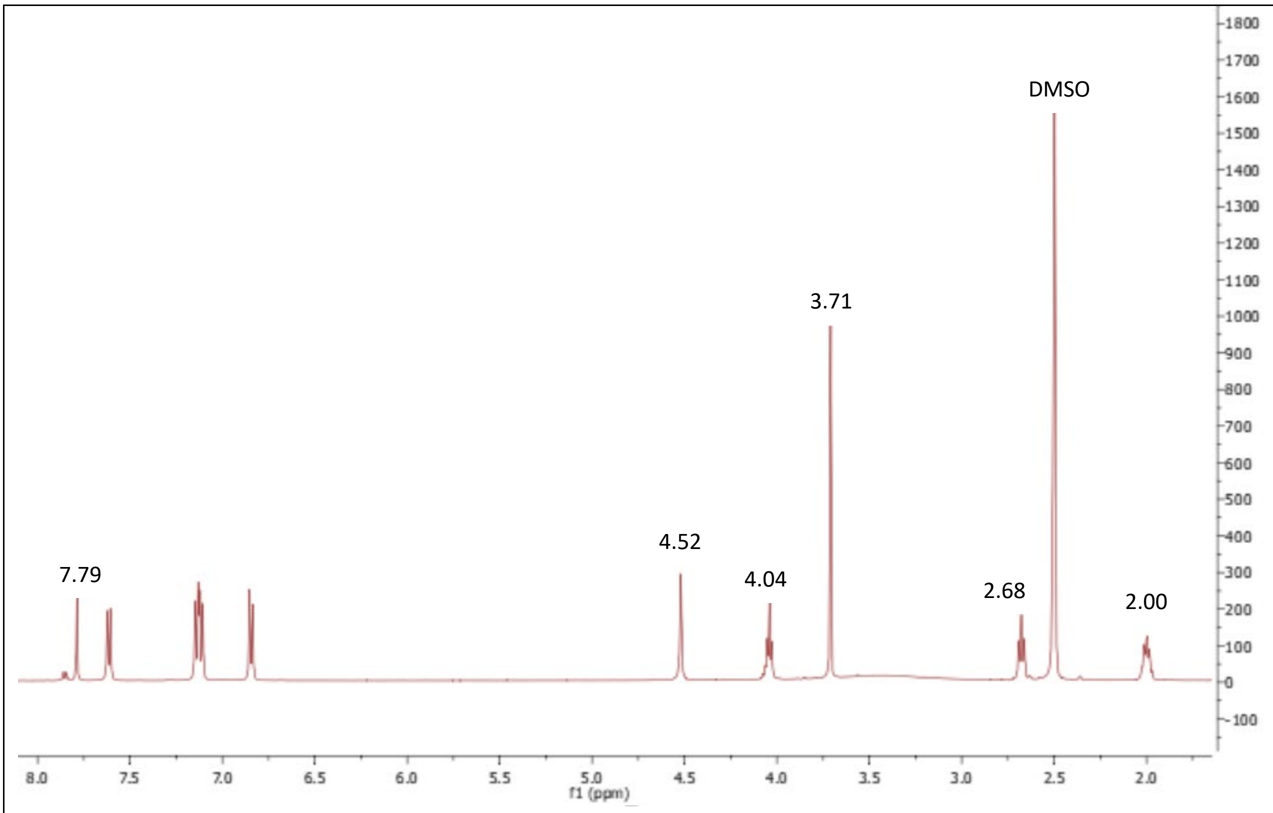

Figure S5. ^{1}H-NMR spectrum of compound **1e** (500 MHz, DMSO-d_6)

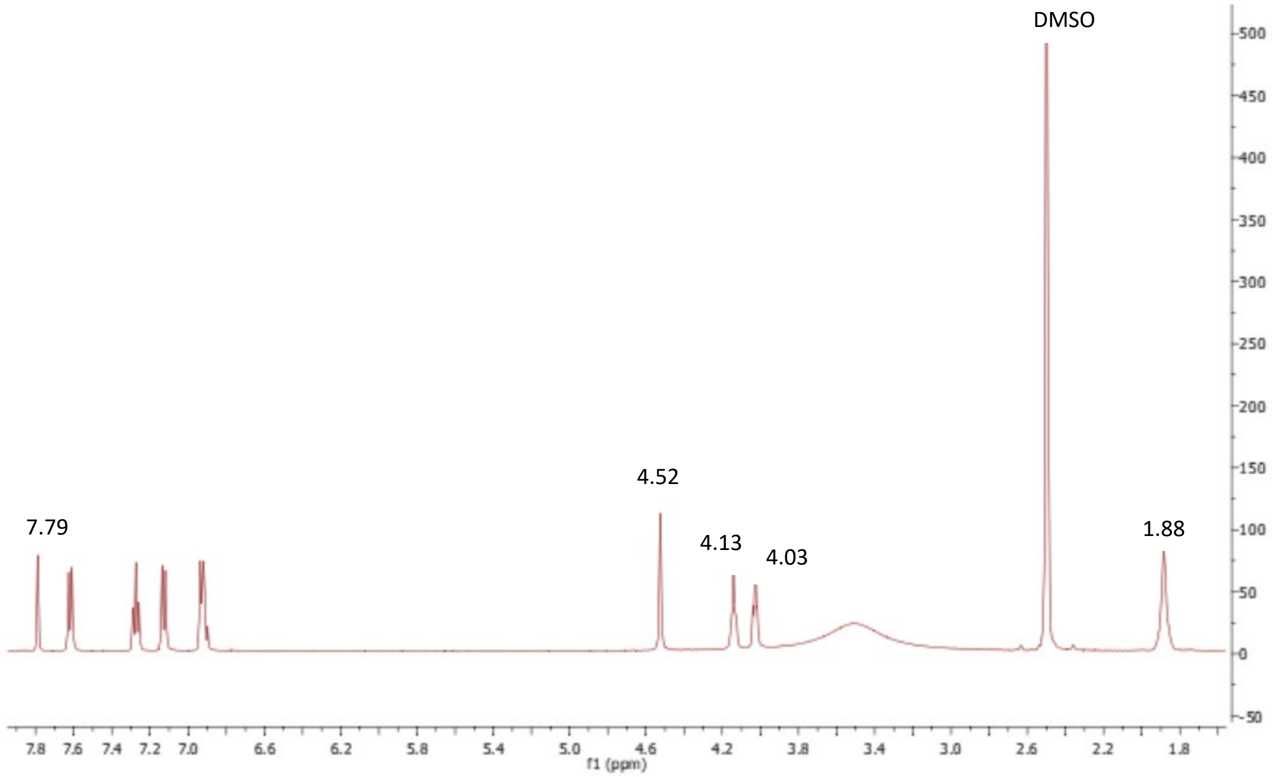

Figure S6. ^{1}H-NMR spectrum of compound **1f** (500 MHz, DMSO-d_6)

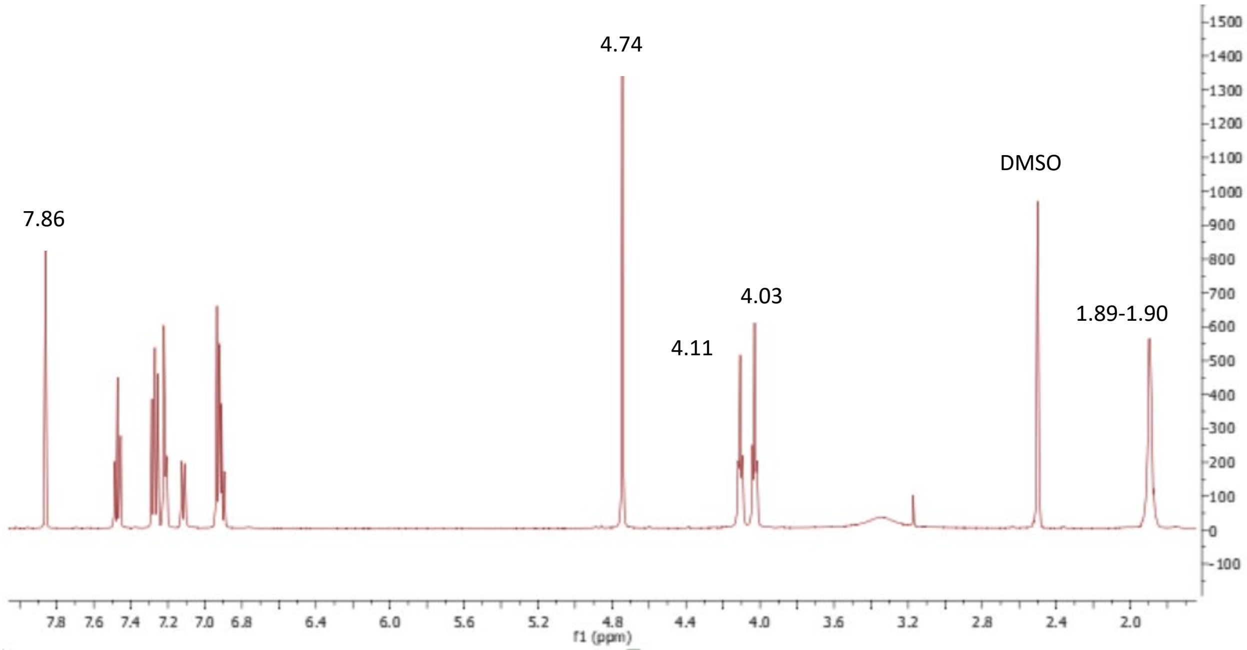

Figure S7. ^{1}H-NMR spectrum of compound **1g** (500 MHz, DMSO-d_6)

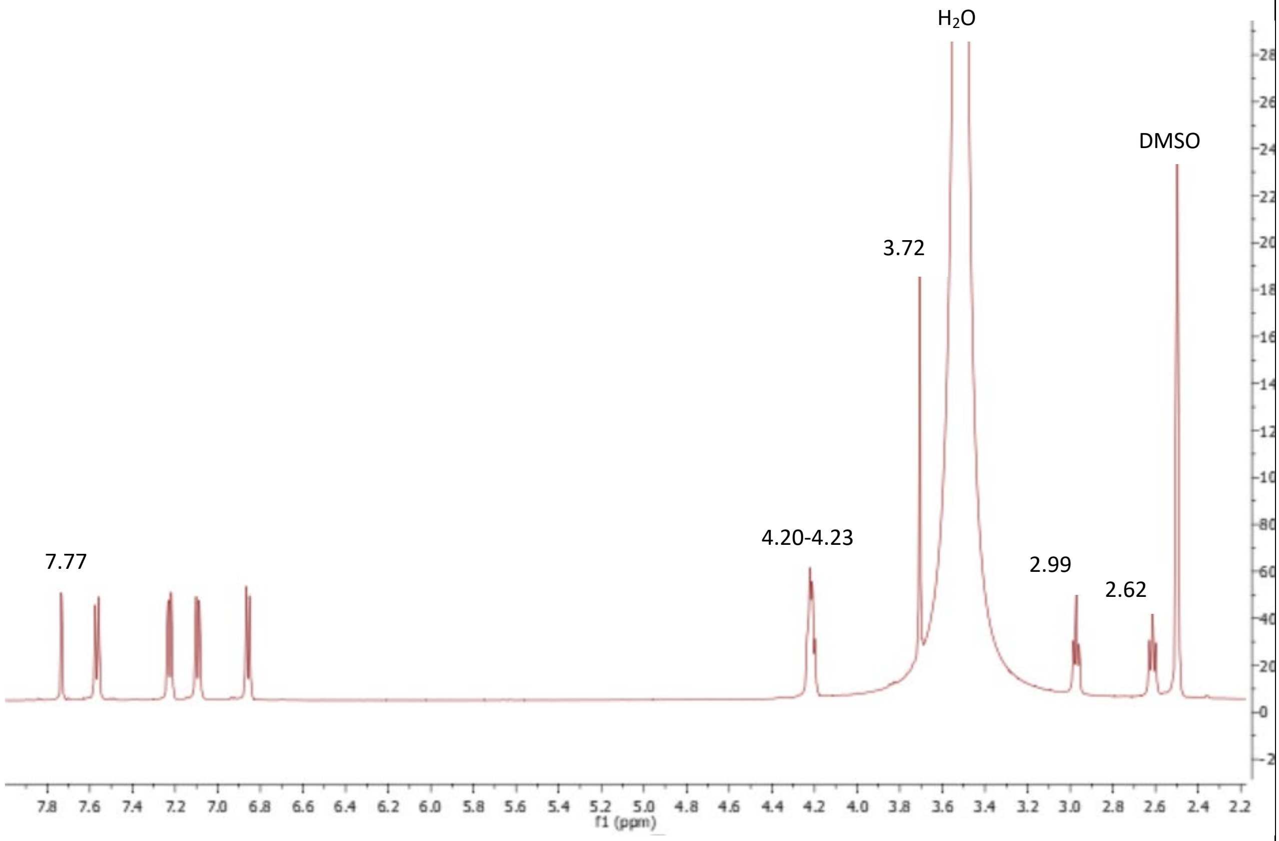

Figure S8. ^{1}H-NMR spectrum of compound **2a** (500 MHz, DMSO-d_6)

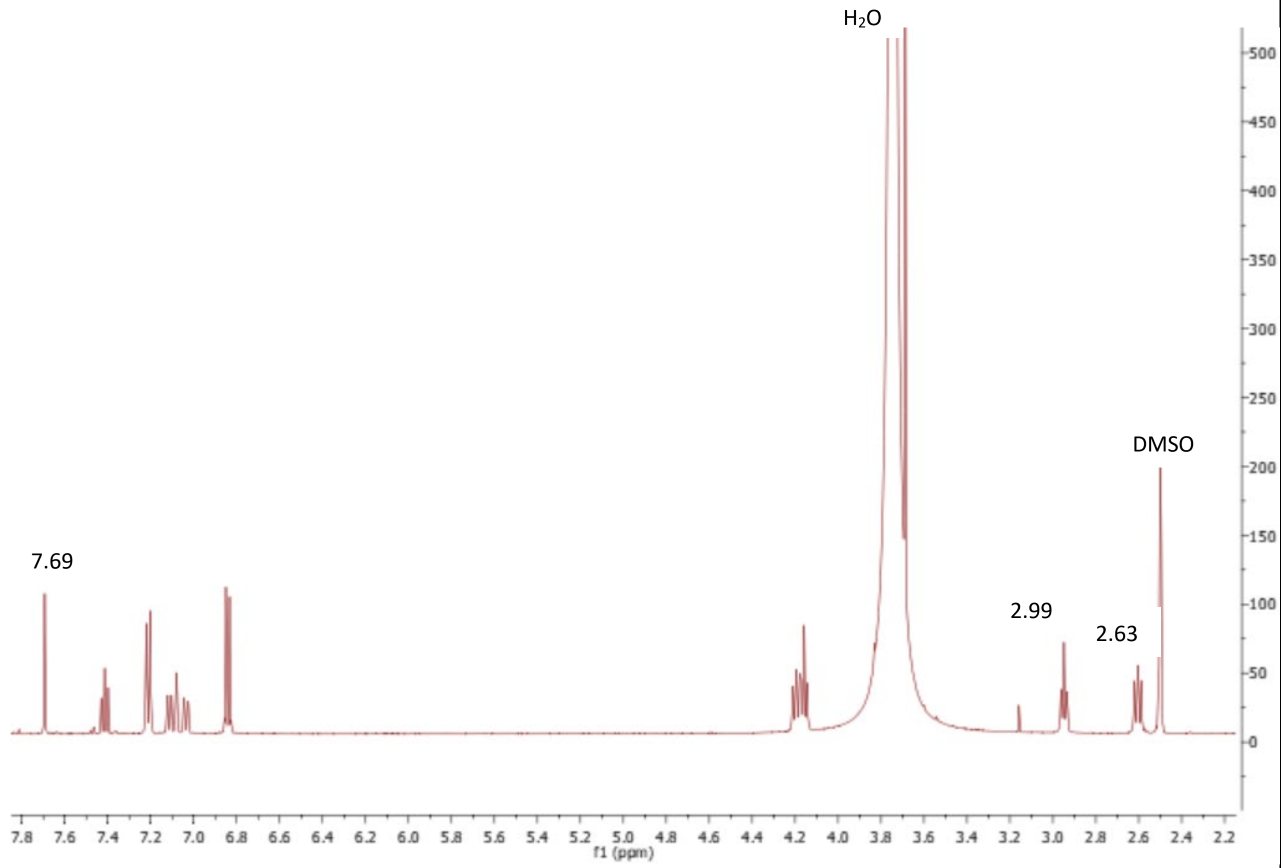

Figure S9. ^{1}H-NMR spectrum of compound **2b** (500 MHz, DMSO-d_6)

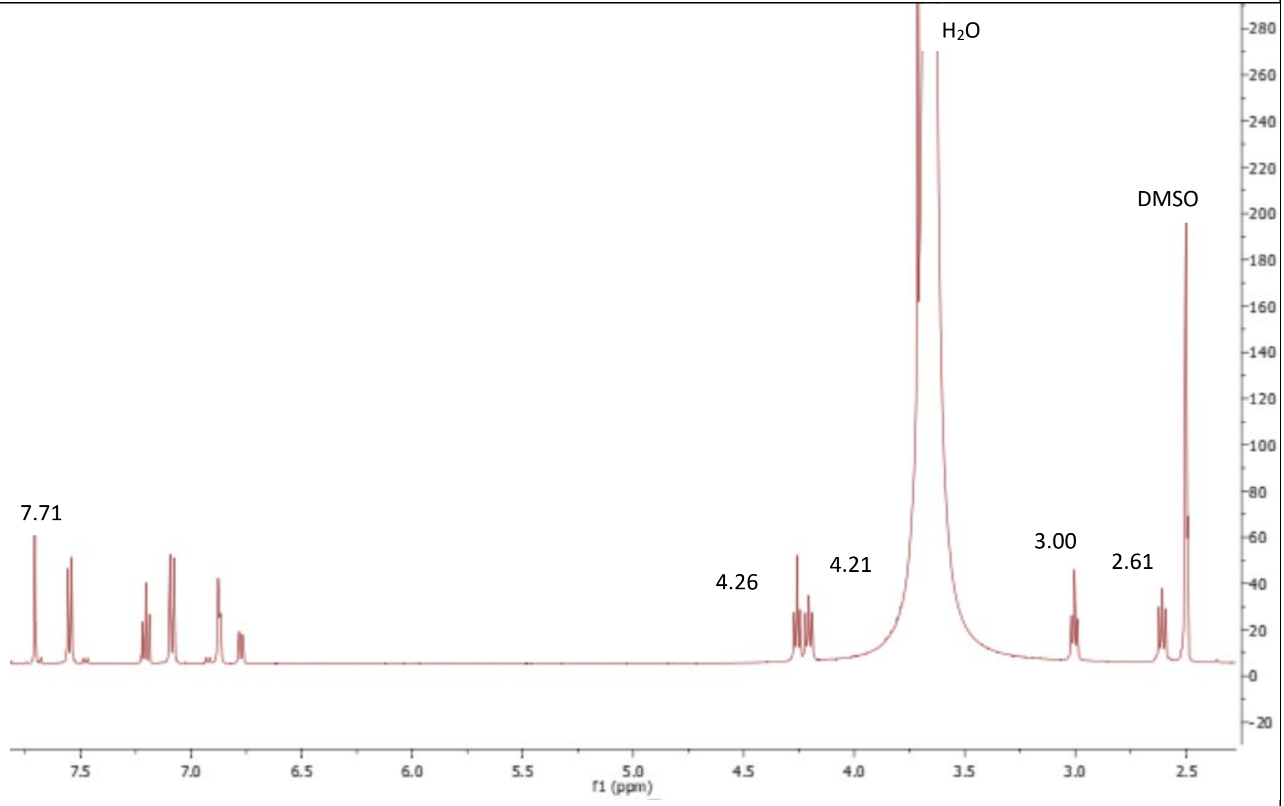

Figure S10. ^{1}H-NMR spectrum of compound **2c** (500 MHz, DMSO-d_6)

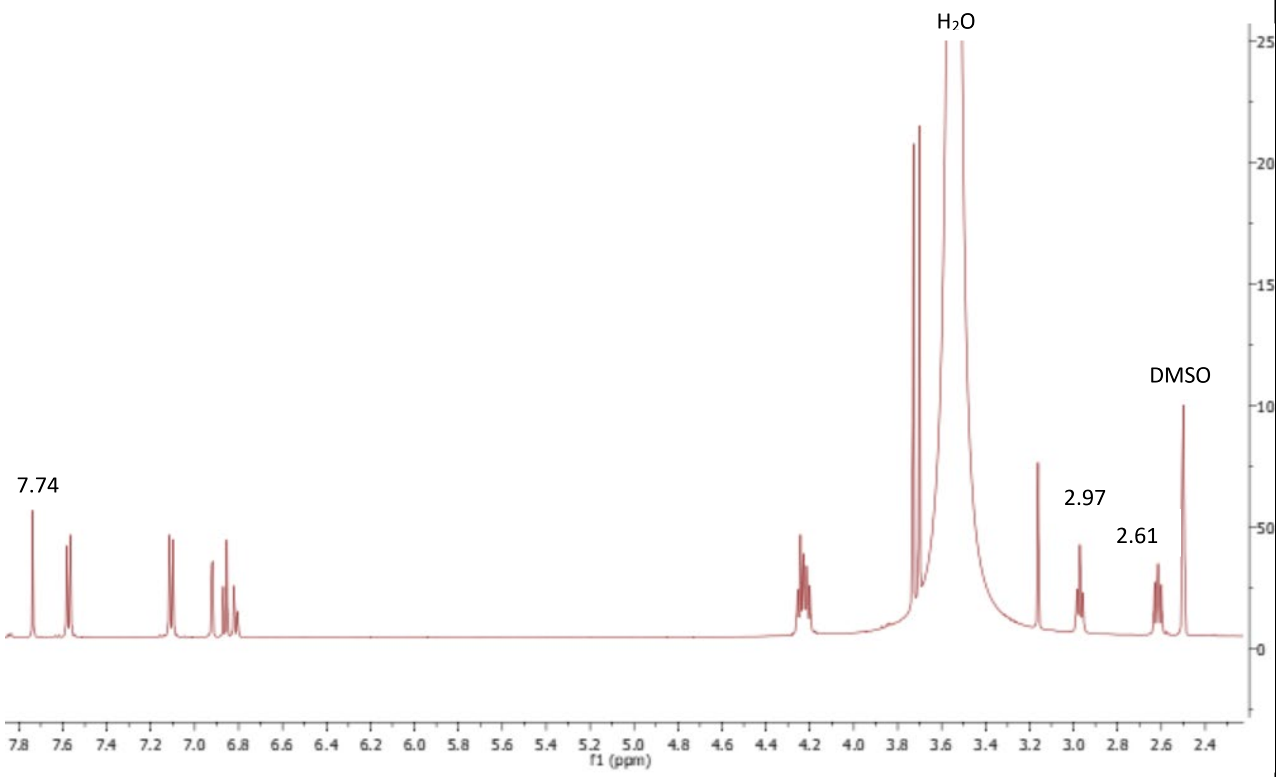

Figure S11. ^{1}H-NMR spectrum of compound **2d** (500 MHz, DMSO-d_6)

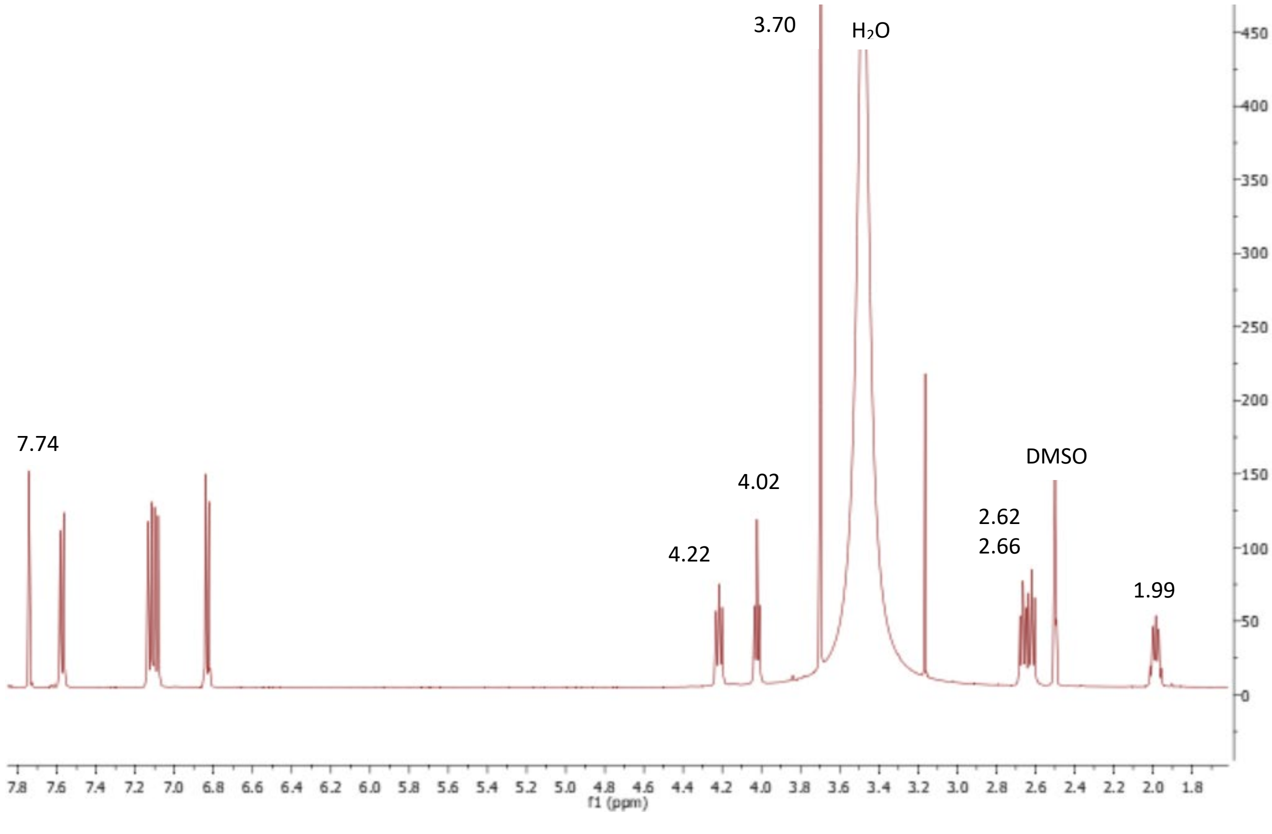

Figure S12. ^{1}H-NMR spectrum of compound **2e** (500 MHz, DMSO-d_6)

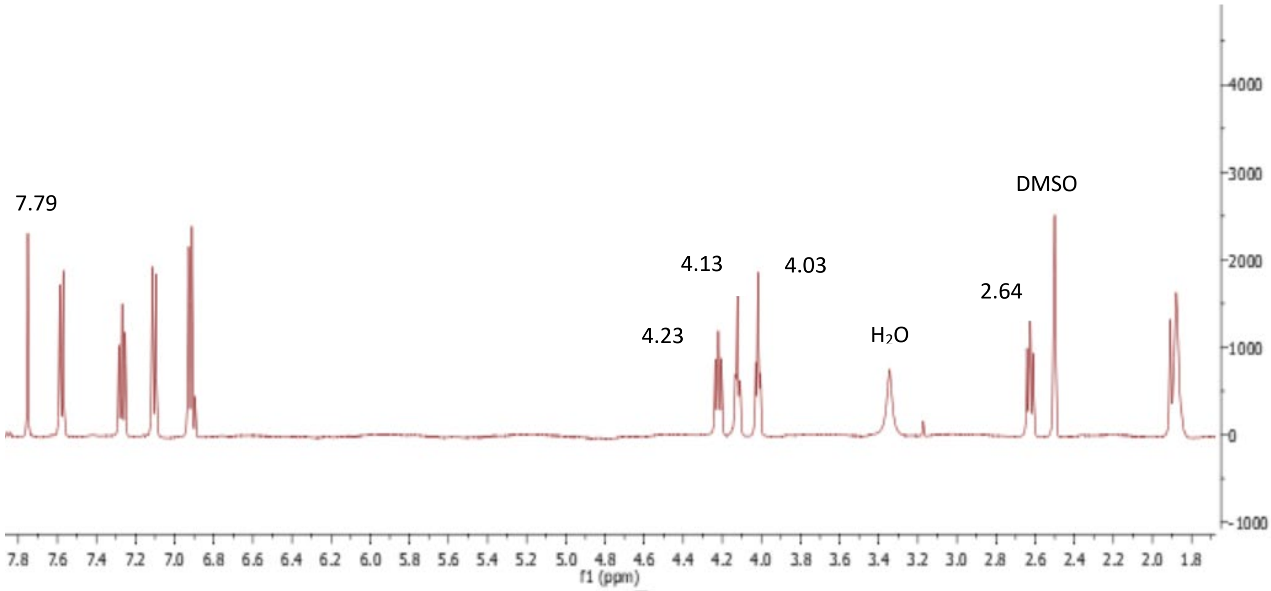

Figure S13. ^{1}H-NMR spectrum of compound **2f** (500 MHz, DMSO-d_6)

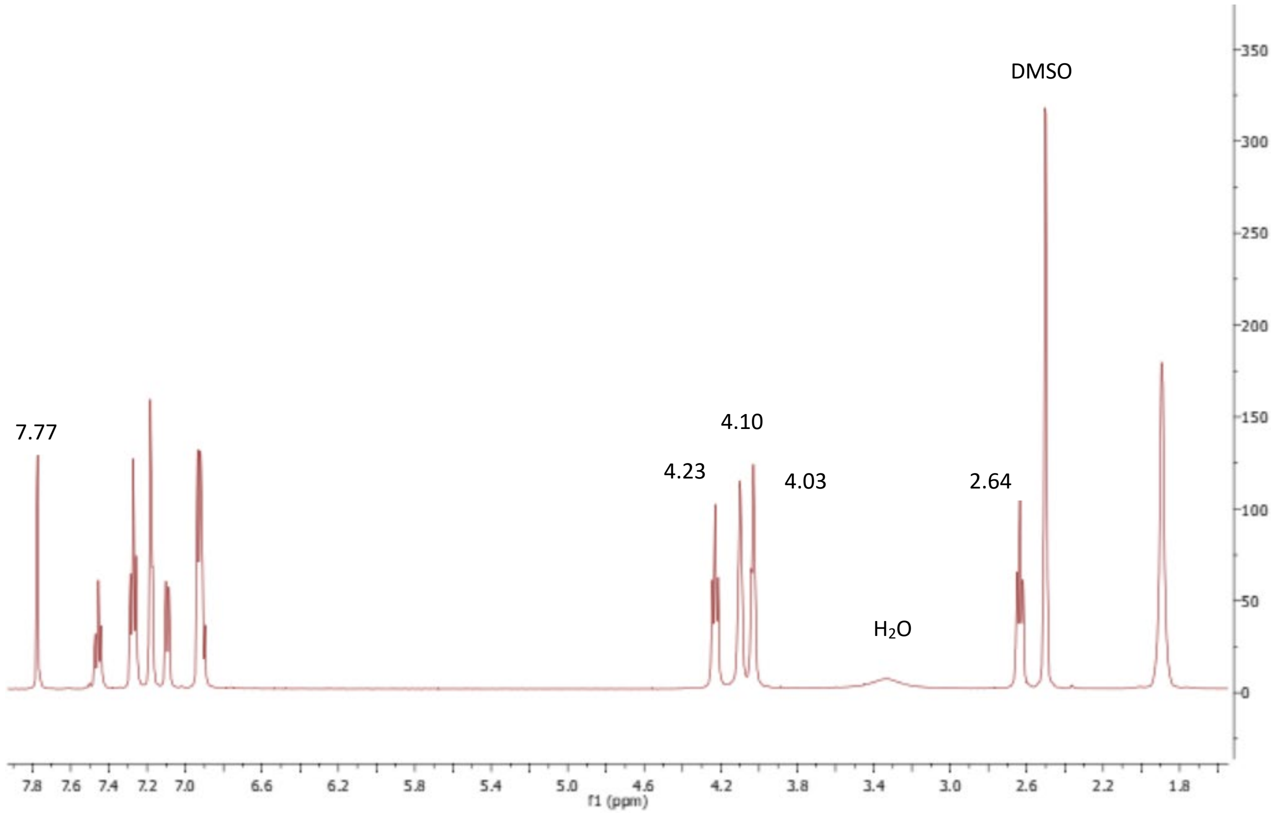

Figure S14. ^{1}H-NMR spectrum of compound **2g** (500 MHz, DMSO-d_6)

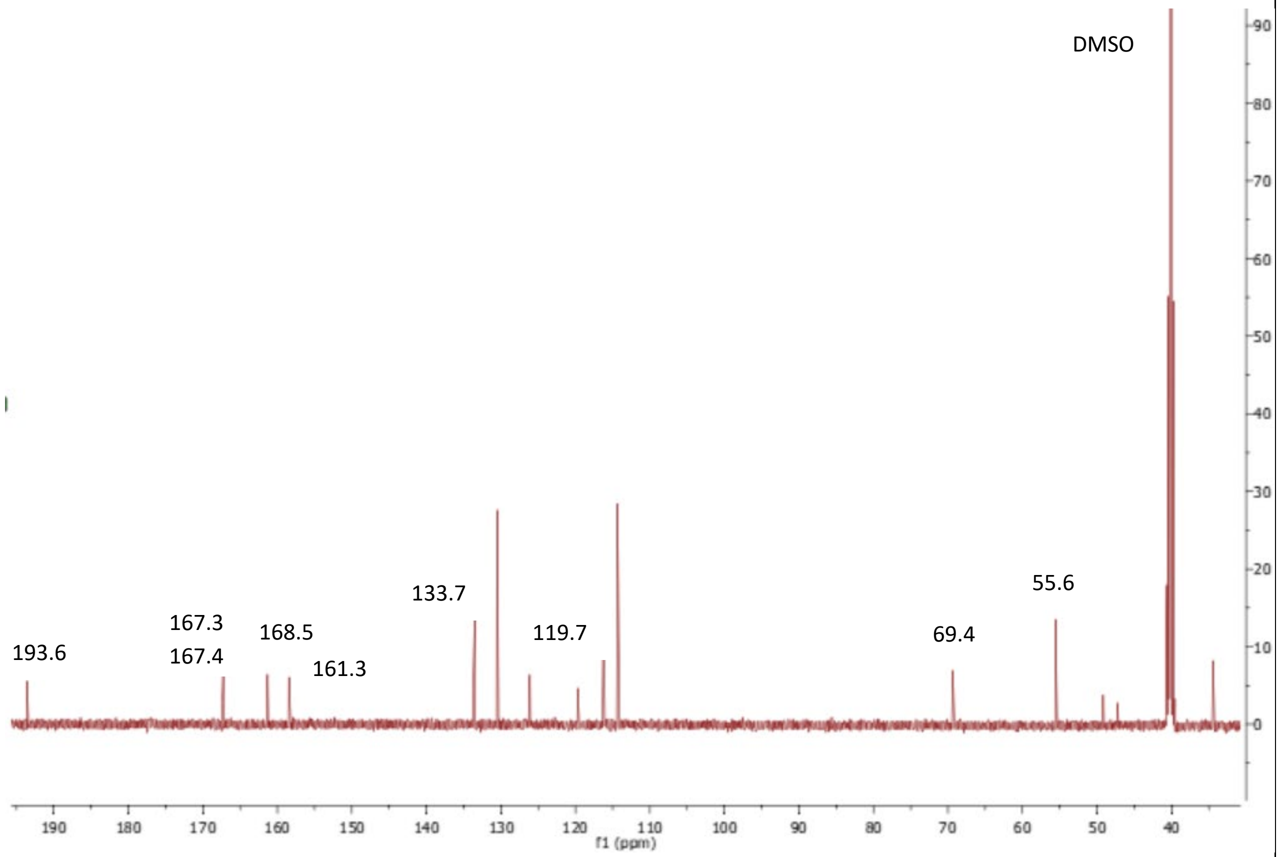

Figure S15. ^{13}C-NMR spectrum of compound **1a** (125.73 MHz, DMSO-d_6)

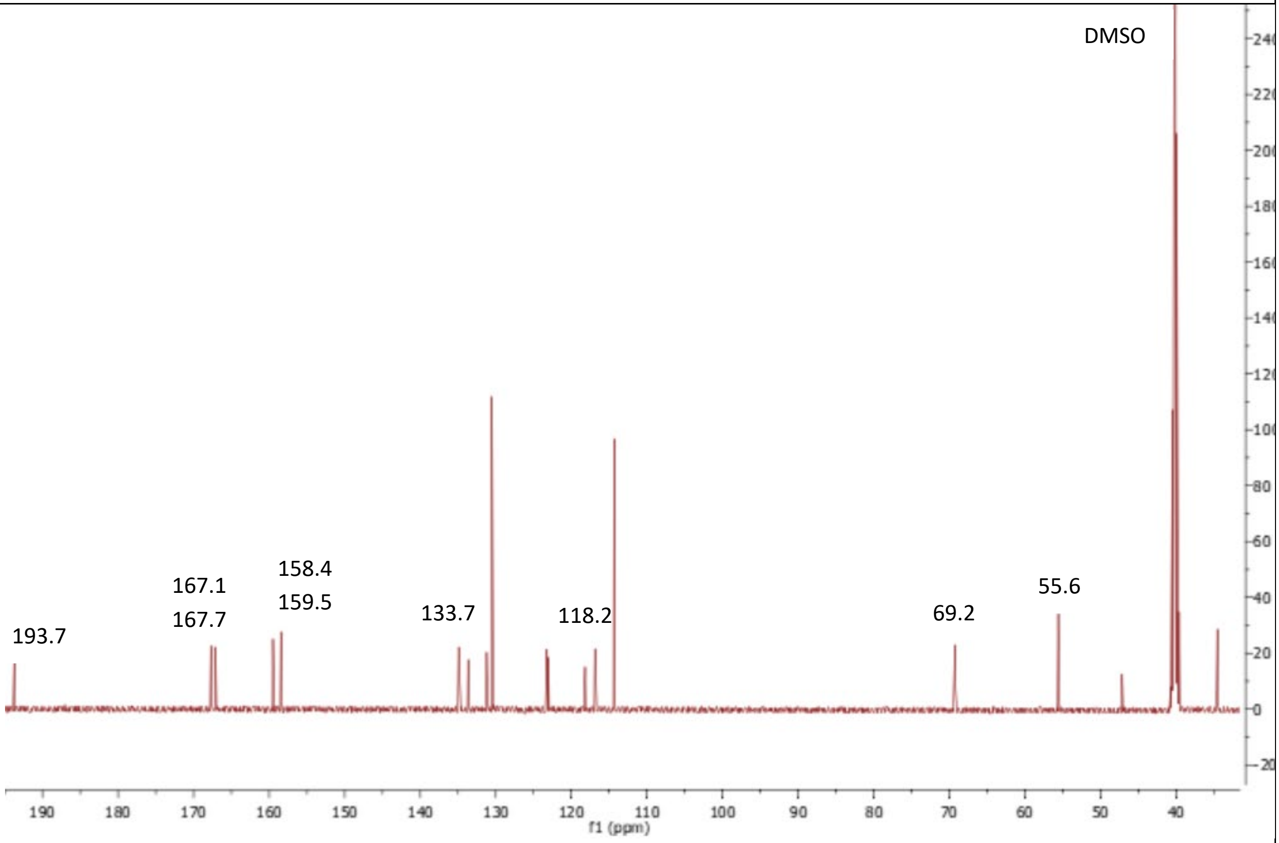

Figure S16. ^{13}C-NMR spectrum of compound **1b** (125.73 MHz, DMSO-d_6)

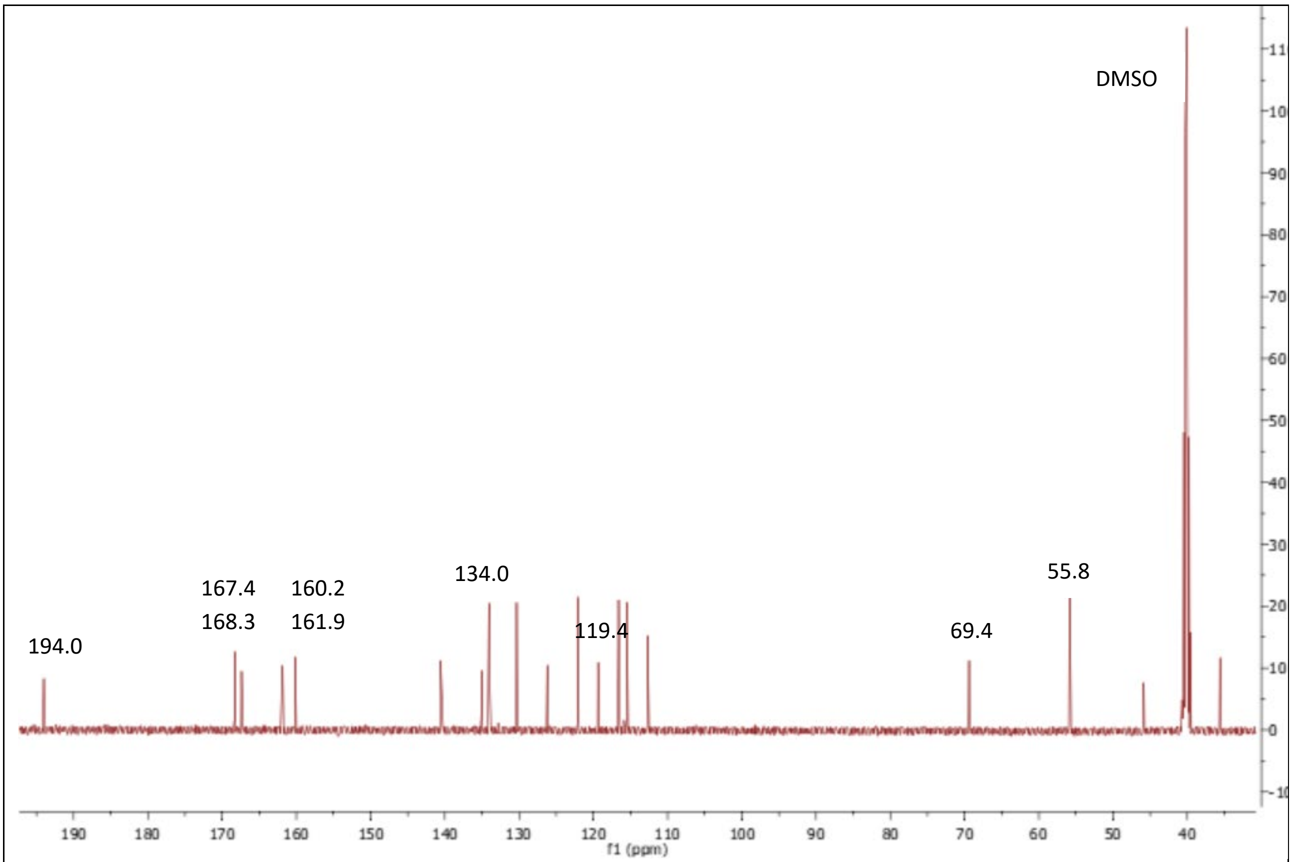

Figure S17. ^{13}C-NMR spectrum of compound **1c** (125.73 MHz, DMSO-d_6)

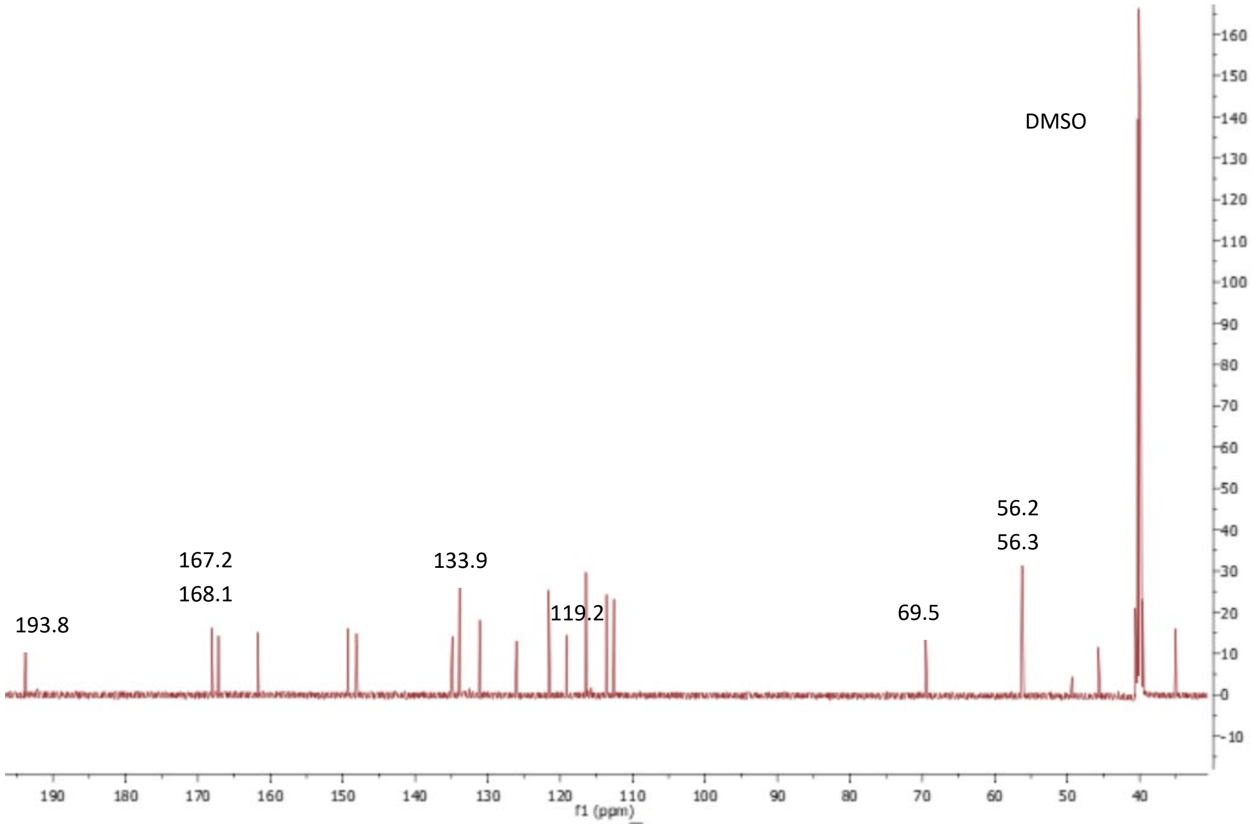

Figure S18. ^{13}C-NMR spectrum of compound **1d** (125.73 MHz, DMSO-d_6)

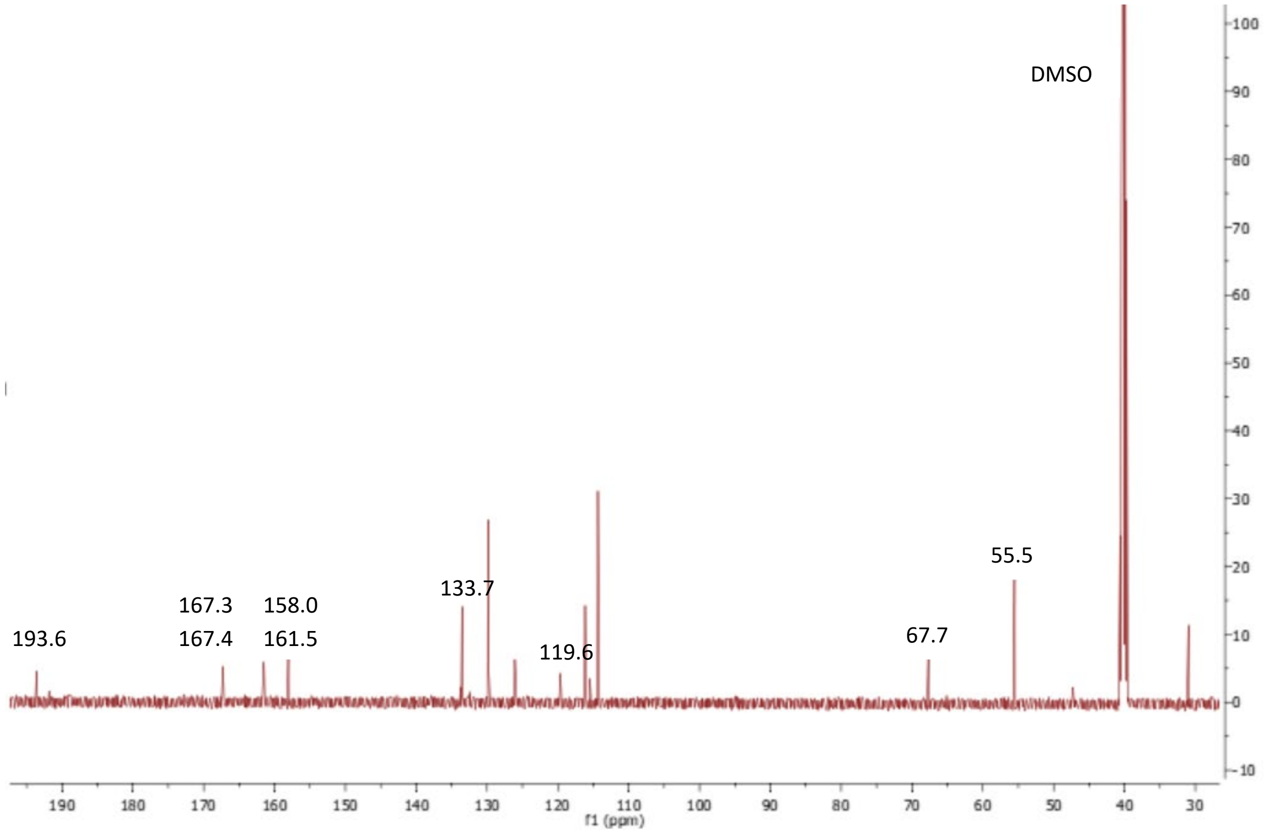

Figure S19. ^{13}C-NMR spectrum of compound **1e** (125.73 MHz, DMSO-d_6)

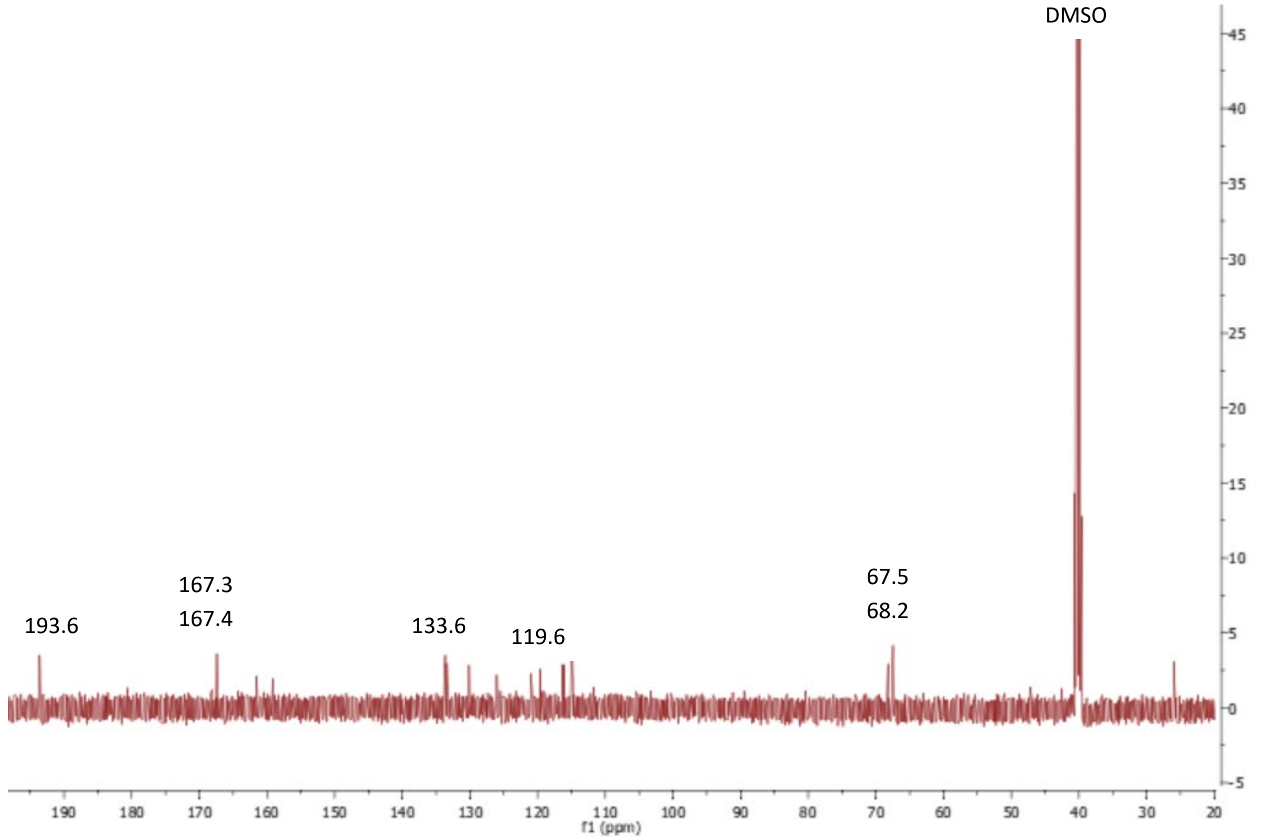

Figure S20. ^{13}C-NMR spectrum of compound **1f** (125.73 MHz, DMSO-d_6)

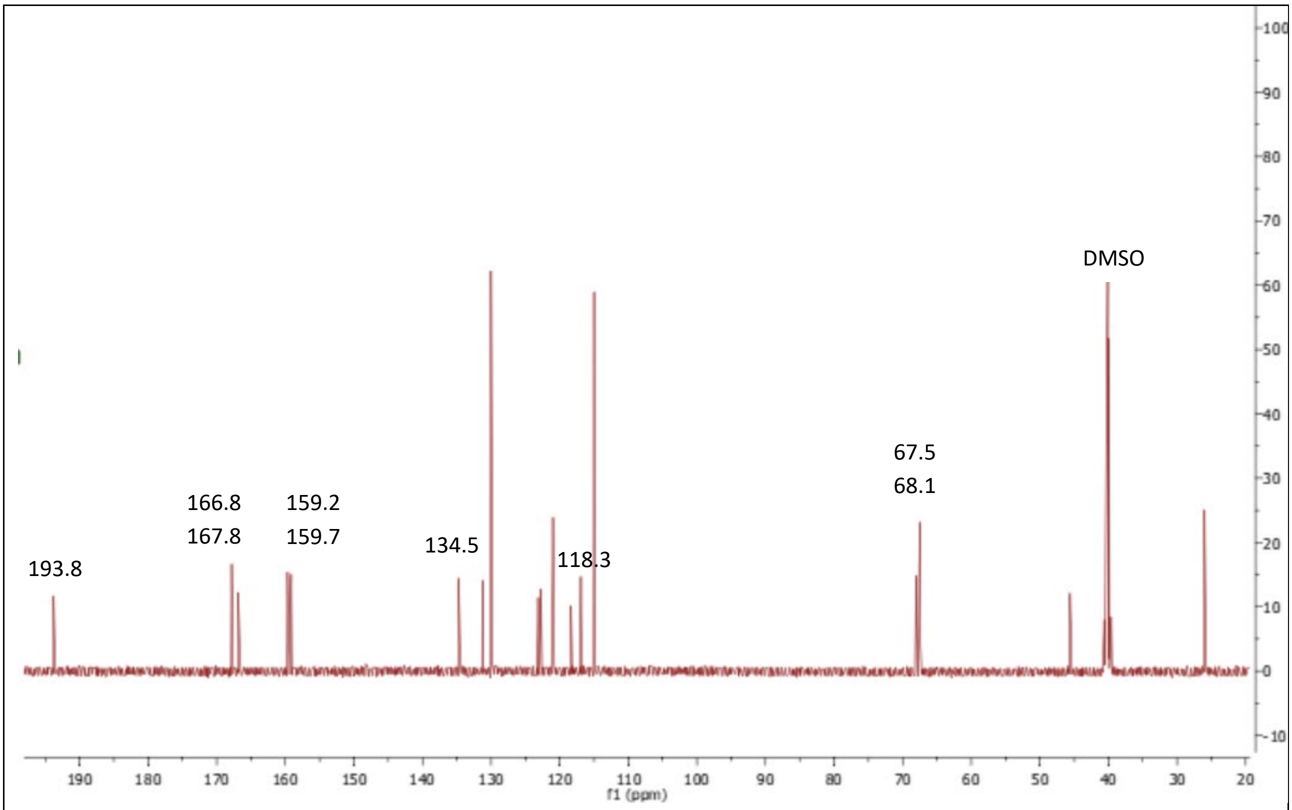

Figure S21. ^{13}C-NMR spectrum of compound **1g** (125.73 MHz, DMSO-d_6)

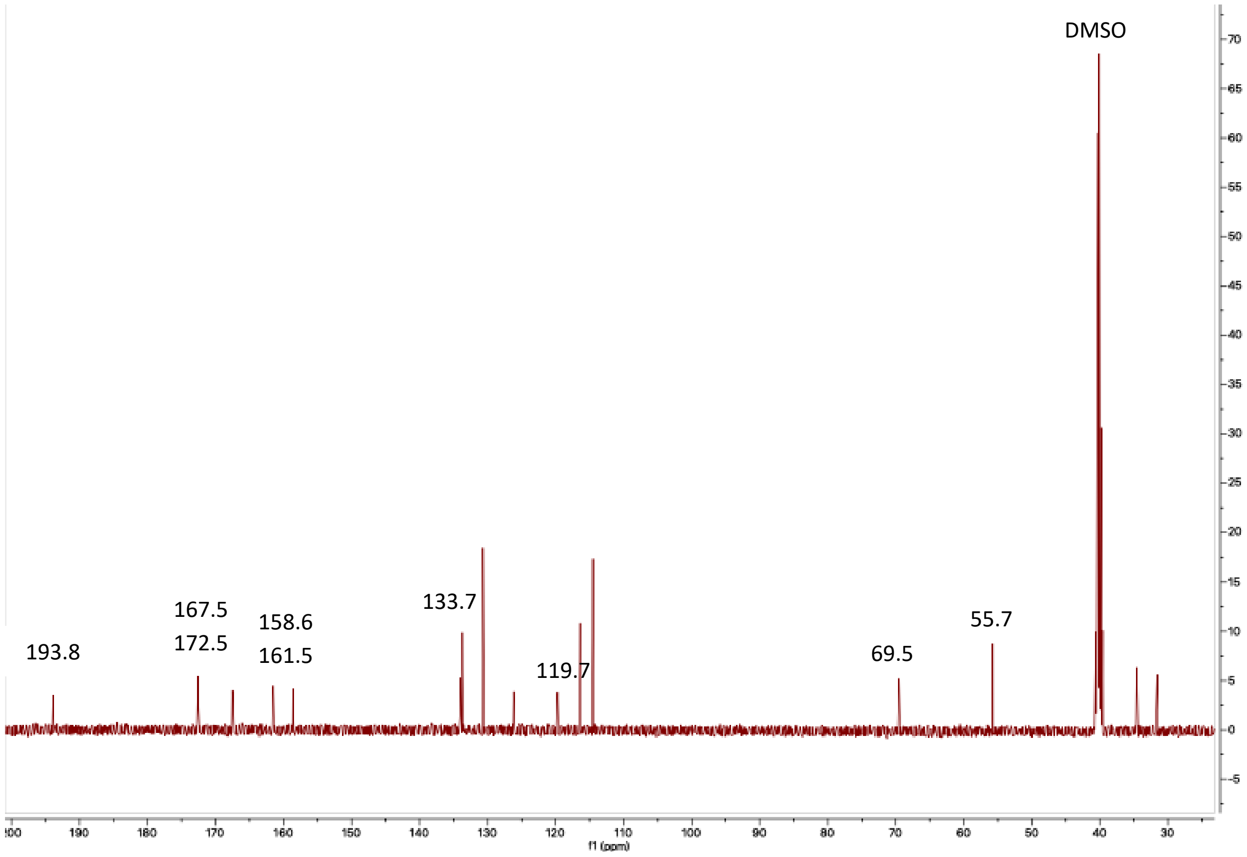

Figure S22. ^{13}C-NMR spectrum of compound **2a** (125.73 MHz, DMSO-d_6)

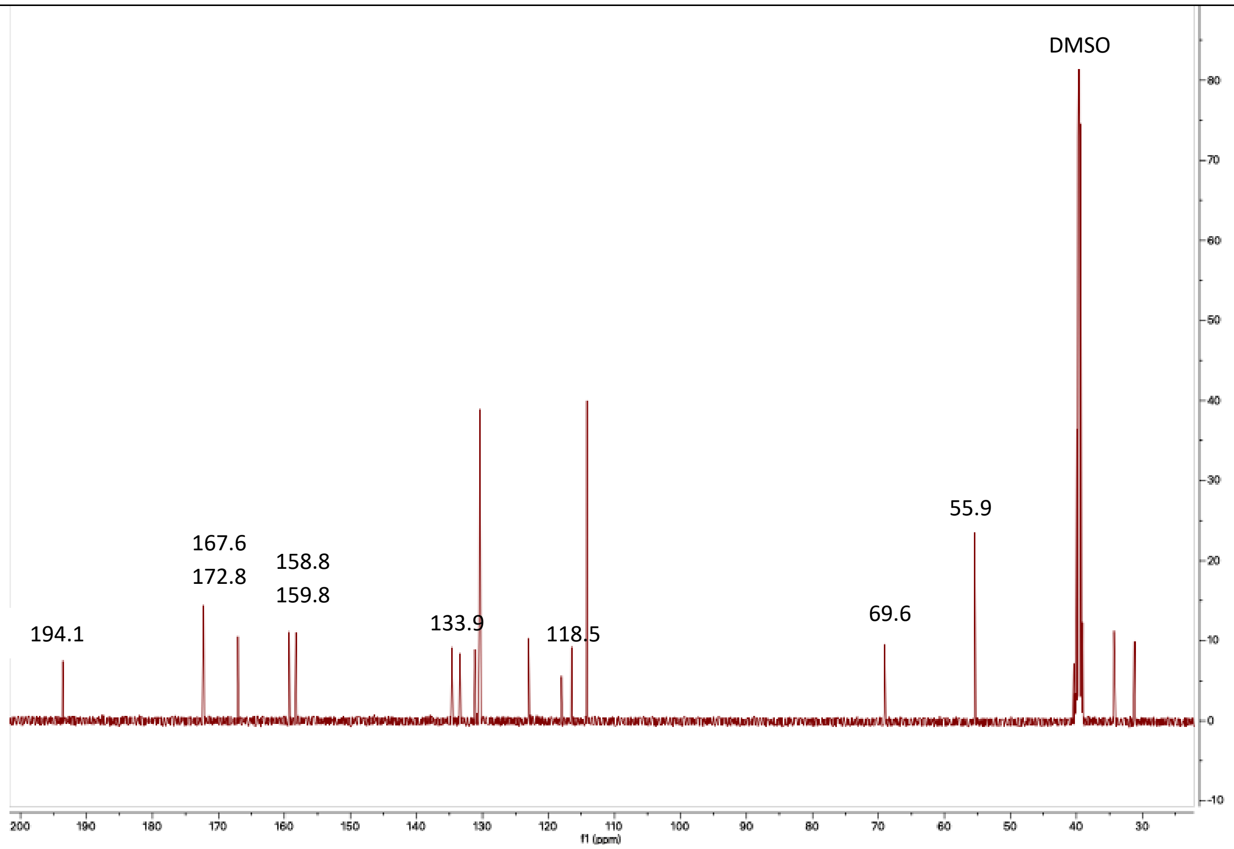

Figure S23. ^{13}C-NMR spectrum of compound **2b** (125.73 MHz, DMSO-d_6)

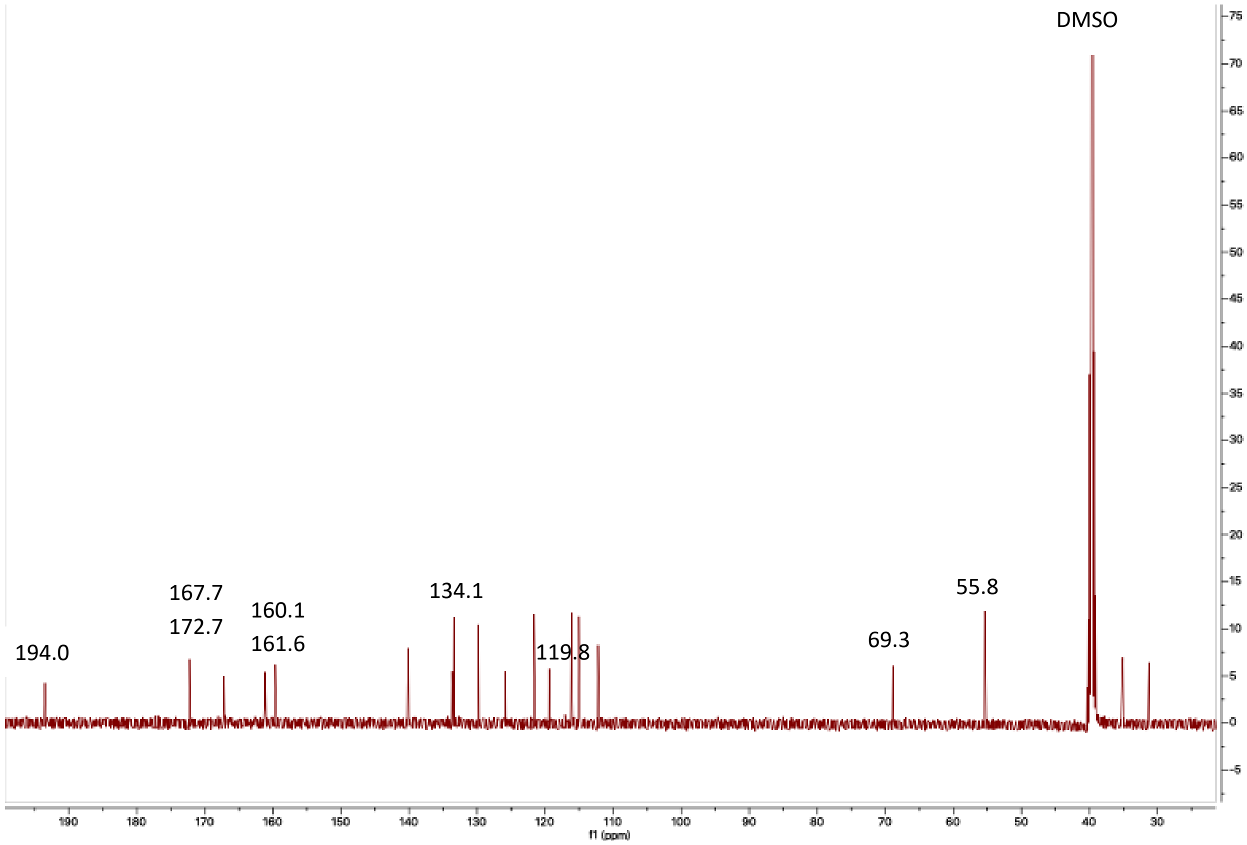

Figure S24. ^{13}C-NMR spectrum of compound **2c** (125.73 MHz, DMSO-d_6)

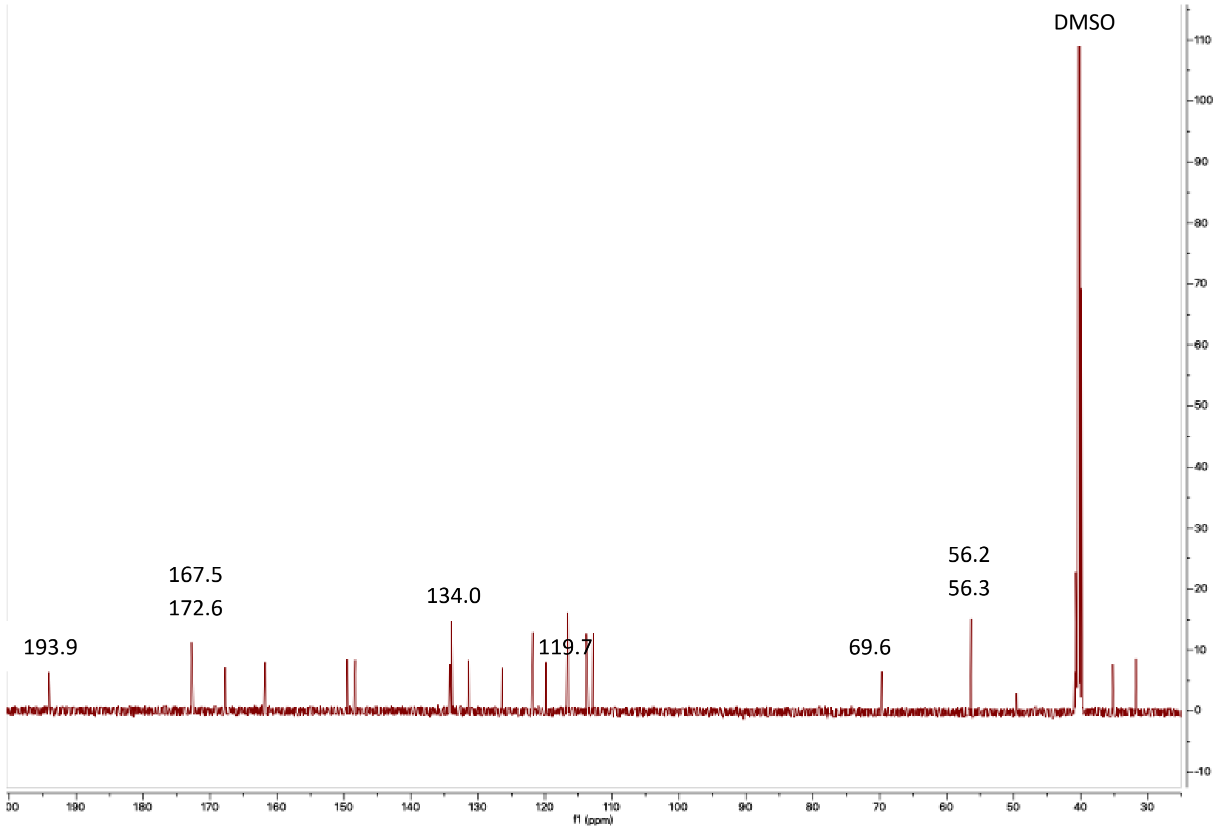

Figure S25. ^{13}C-NMR spectrum of compound **2d** (125.73 MHz, DMSO-d_6)

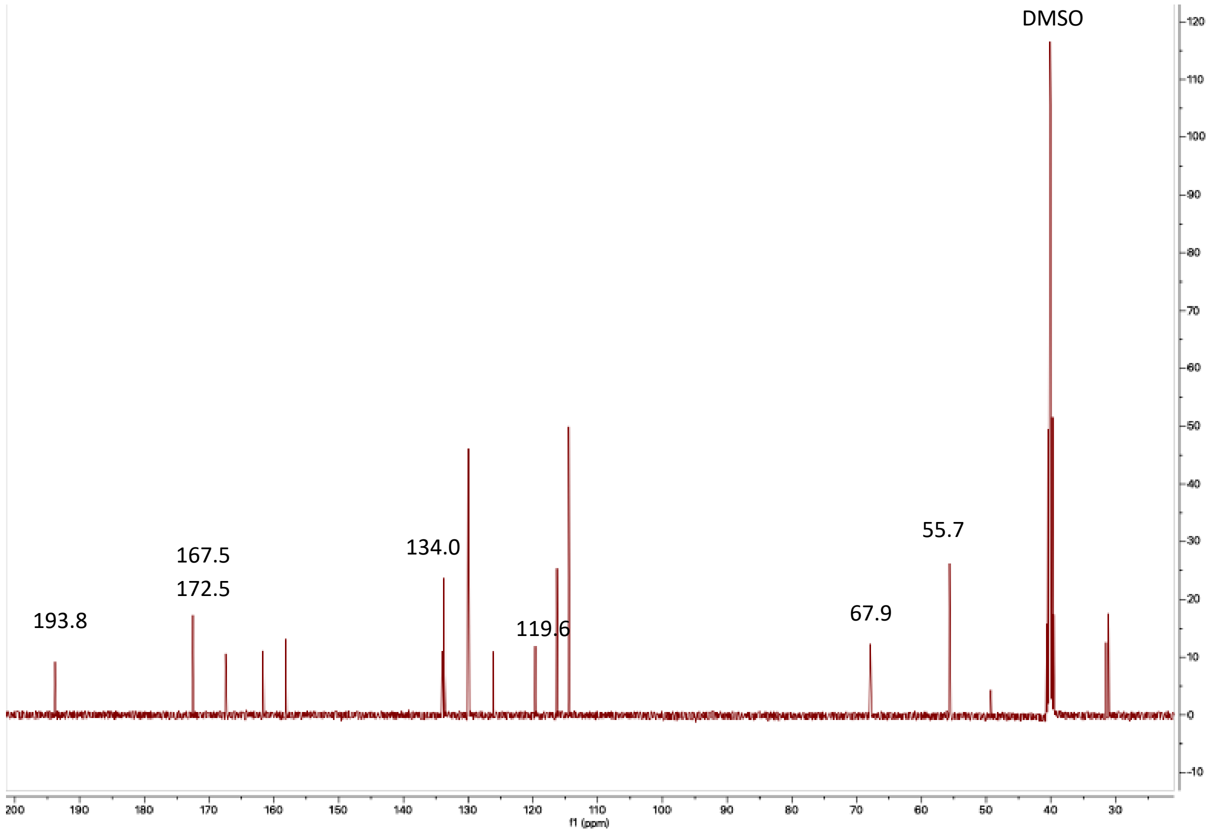

Figure S26. ^{13}C-NMR spectrum of compound **2e** (125.73 MHz, DMSO-d_6)

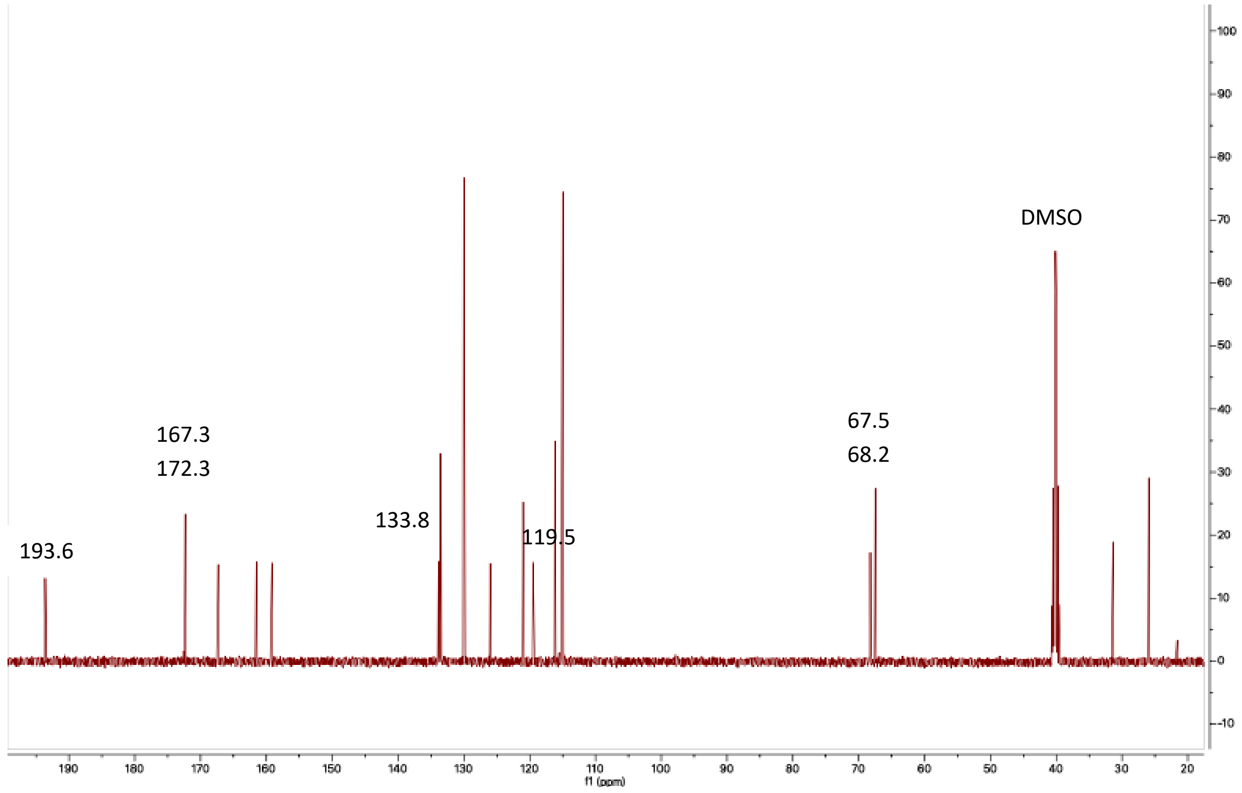

Figure S27. ^{13}C-NMR spectrum of compound **2f** (125.73 MHz, DMSO-d_6)

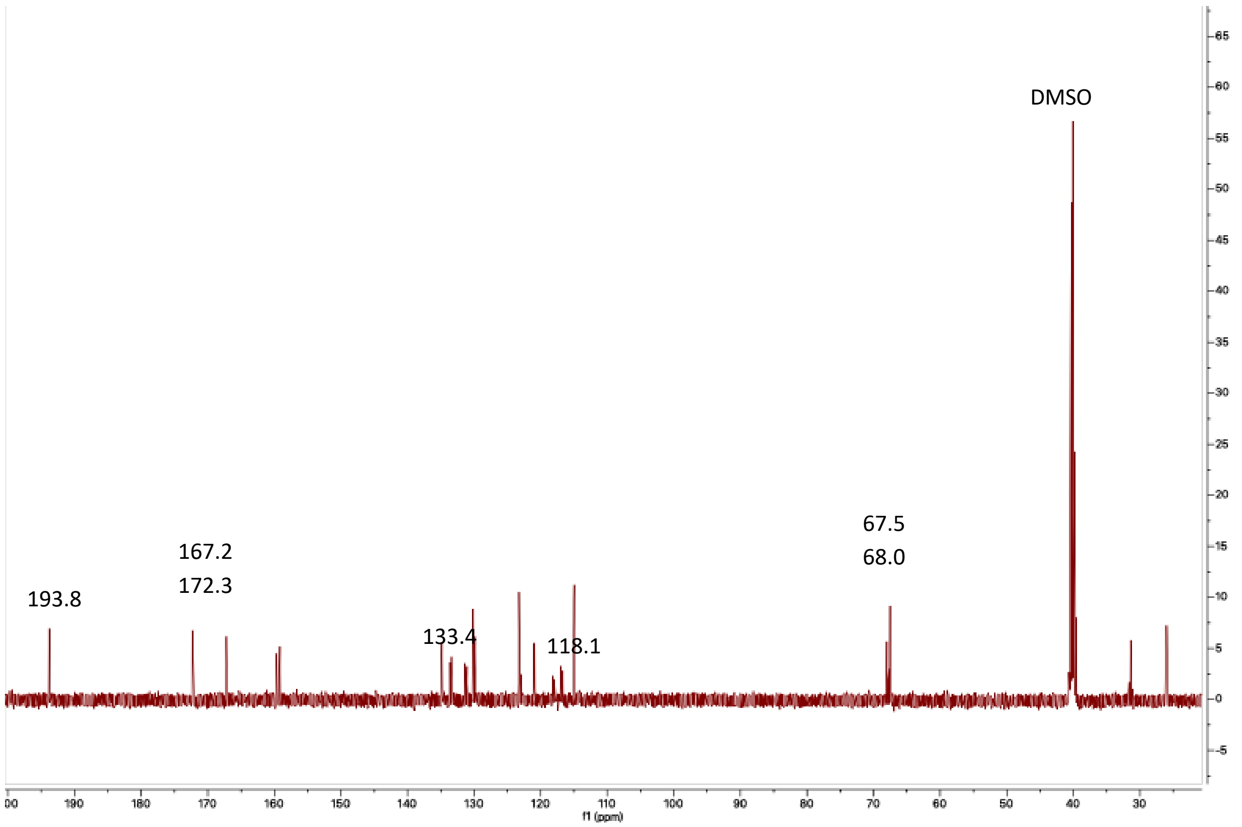

Figure S28. ^{13}C-NMR spectrum of compound **2g** (125.73 MHz, DMSO-d_6)

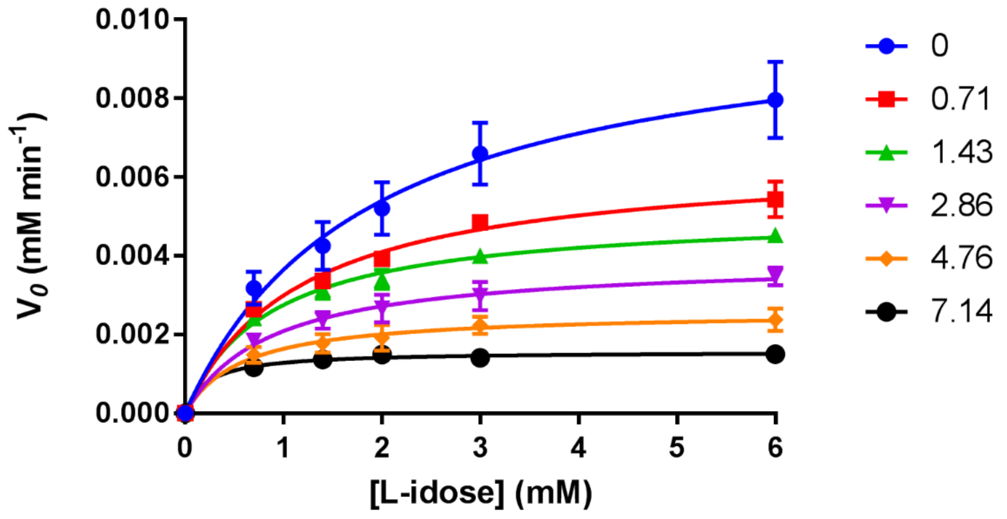

Figure S29. Rate measurements of the AKR1B1 dependent reduction of L-idose in the presence of compound **2e**. The activity of AKR1B1 (10 mU in the assay) was measured using, as substrate, the indicated L-idose concentrations, in the presence of the µM concentrations of compound **2e** reported alongside. Error bars (when not visible are within the symbol size) represent the standard deviations of the mean from at least three independent experiments.

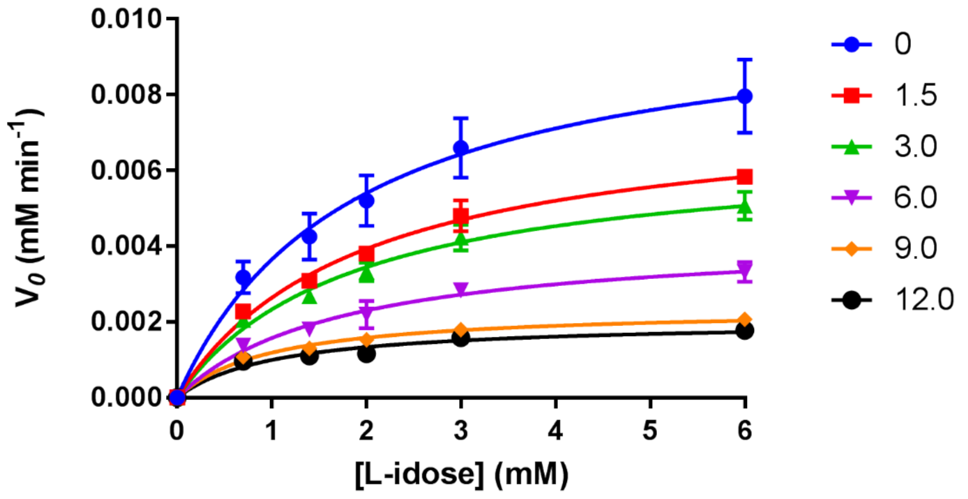

Figure S30. Rate measurements of the AKR1B1 dependent reduction of L-idose in the presence of compound **2f**. The activity of AKR1B1 (10 mU in the assay) was measured using, as substrate, the indicated L-idose concentrations, in the presence of the µM concentrations of compound **2f** reported alongside. Error bars (when not visible are within the symbol size) represent the standard deviations of the mean from at least three independent experiments.

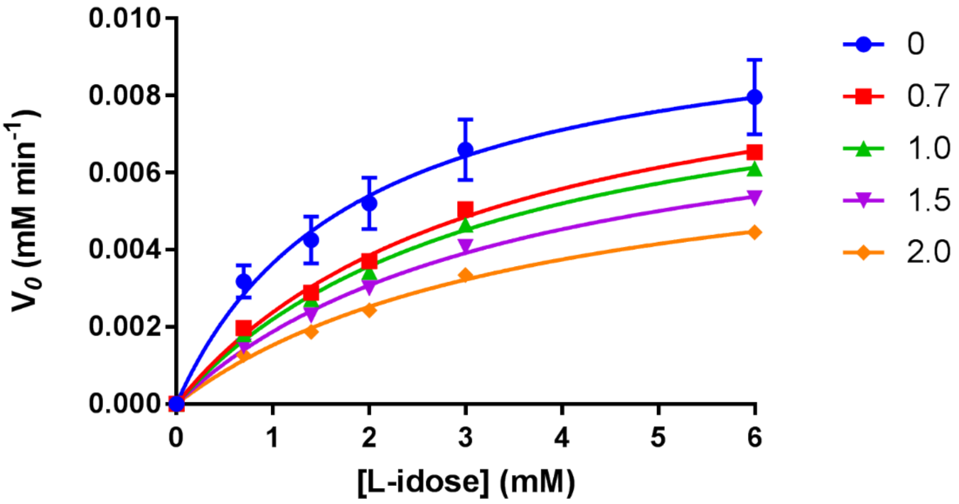

Figure S31. Rate measurements of the AKR1B1 dependent reduction of L-idose in the presence of compound **2g**. The activity of AKR1B1 (10 mU in the assay) was measured using, as substrate, the indicated L-idose concentrations, in the presence of the µM concentrations of compound **2g** reported alongside. Error bars (when not visible are within the symbol size) represent the standard deviations of the mean from at least three independent experiments.

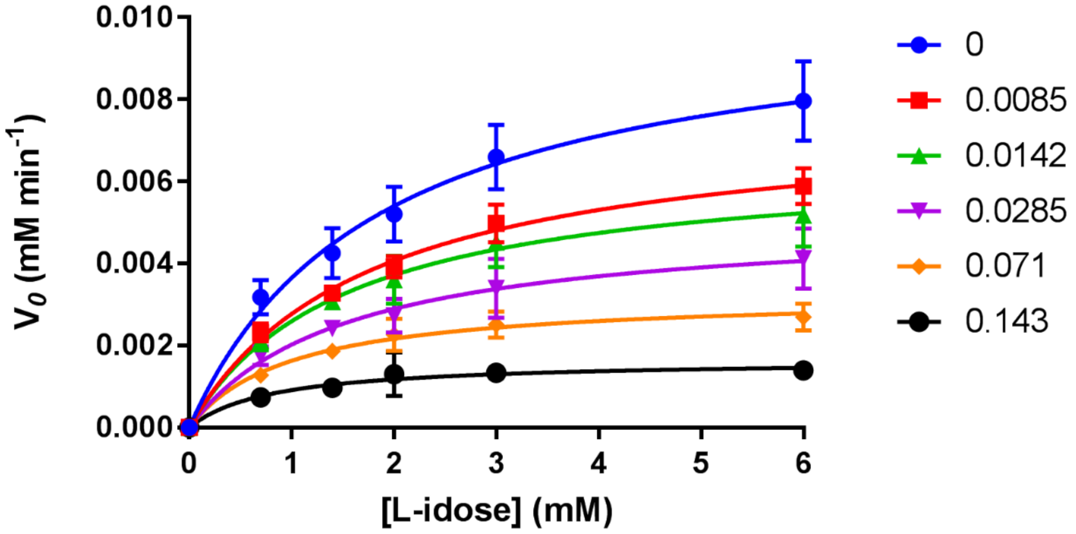

Figure S32. Rate measurements of the AKR1B1 dependent reduction of L-idose in the presence of compound **1g**. The activity of AKR1B1 (10 mU in the assay) was measured using, as substrate, the indicated L-idose concentrations, in the presence of the µM concentrations of compound **1g** reported alongside. Error bars (when not visible are within the symbol size) represent the standard deviations of the mean from at least three independent experiments.

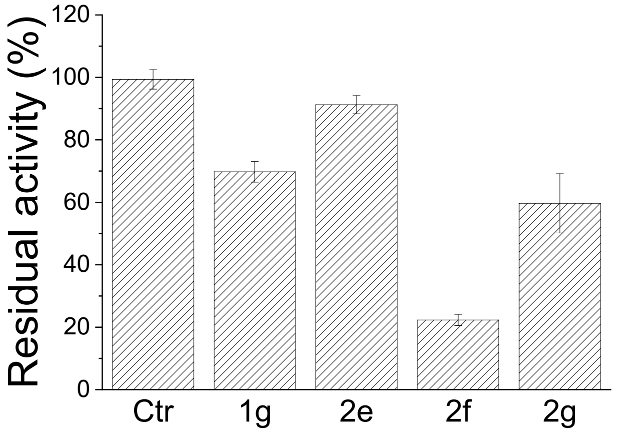

Figure S33. Dilution assay. An aliquot of PTP1B was incubated for 1h at 37°C in the presence of saturating concentrations of compounds **1g** and **2e-g**. Then, an aliquot of solution was diluted 700 folds in the assay buffer containing 2.5 mM pNPP to evaluate residual activity of the enzyme. After 20 min assay, the absorbance of samples was read at 405 nm using a spectrophotometer.

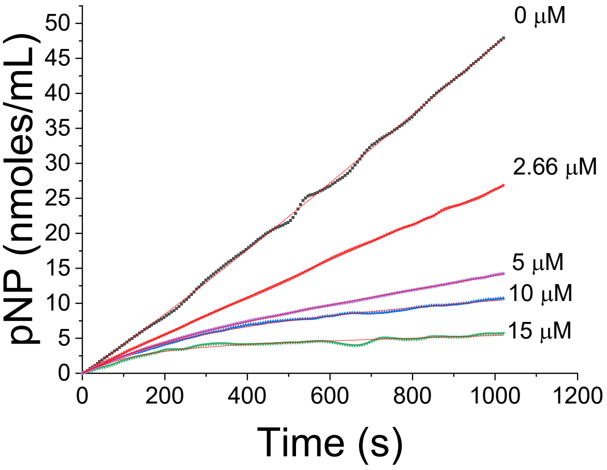

Figure S34. Continuous inhibition of PTP1B by compound **1g** at pH 7.0 and 25°C. Substrate (5 mM, pNPP) hydrolysis was monitored by measuring the release of p-nitrophenol (pNP) into the solution, recording the absorbance of the samples at 400 nm. Subsequently, using the ε_{mM} value (18 $mM^{-1}cm^{-1}$) the absorbance data were converted to pNP concentration values. All reactions were initiated by adding enzyme (0.8 nM PTP1B) to the assay solution. The final concentrations of compound **1g** used are indicated in the figure.

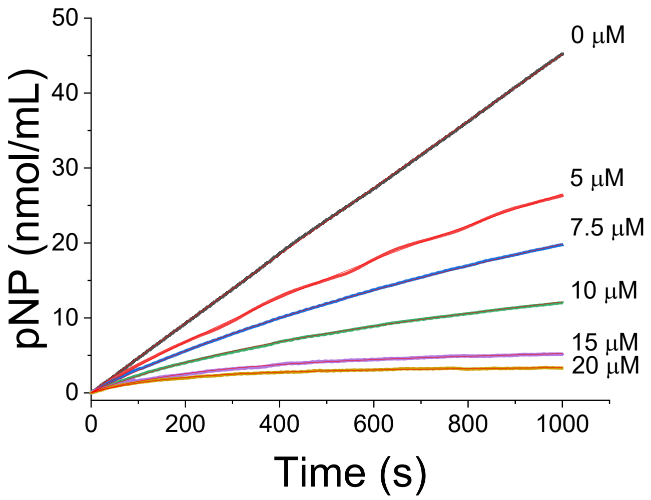

Figure S35. Continuous inhibition of PTP1B by compound **2f** at pH 7.0 and 25°C. Substrate (5 mM, pNPP) hydrolysis was monitored by measuring the release of p-nitrophenol (pNP) into the solution, recording the absorbance of the samples at 400 nm. Subsequently, using the ε_{mM} value (18 $mM^{-1}cm^{-1}$) the absorbance data were converted to pNP concentration values. All reactions were initiated by adding enzyme (0.8 nM PTP1B) to the assay solution. The final concentrations of compound **2f** used are indicated in the figure.

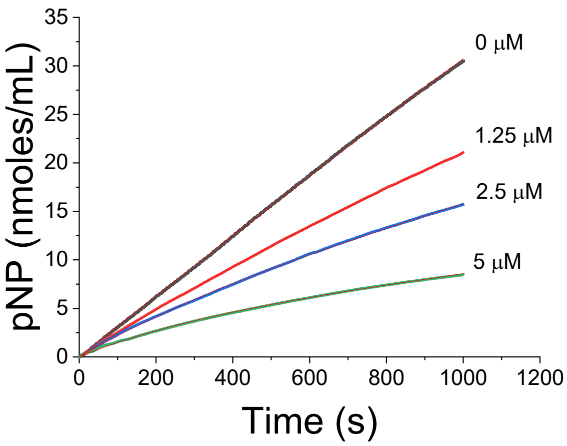

Figure S36. Continuous inhibition of PTP1B by compound **2g** at pH 7.0 and 25°C. Substrate (5 mM, pNPP) hydrolysis was monitored by measuring the release of p-nitrophenol (pNP) into the solution, recording the absorbance of the samples at 400 nm. Subsequently, using the ε_{mM} value (18 $mM^{-1}cm^{-1}$) the absorbance data were converted to pNP concentration values. All reactions were initiated by adding enzyme (0.8 nM PTP1B) to the assay solution. The final concentrations of compound **2g** used are indicated in the legend of the figure.

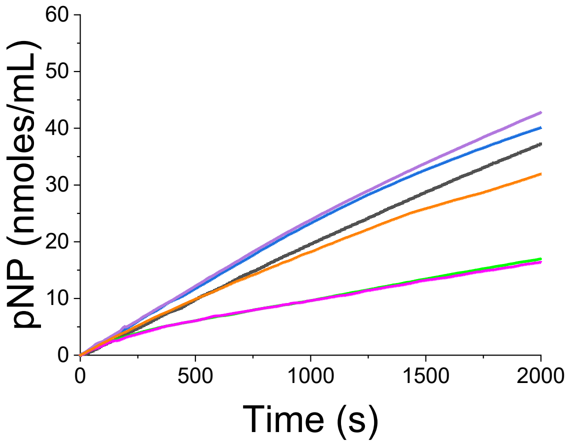

Figure S37. Continuous inhibition of PTP1B by compound **1g** in presence of increasing concentrations of the substrate**.** The hydrolysis of substrate (pNPP) was monitored by measuring the release of p-nitrophenol (pNP) into the solution in the presence of a fixed concentration (5 µM) and increasing amount of pNPP. The coulured curves represent the hydrolysis rate of solutions containing 5 mM pNPP (orange symbols), 5 mM pNPP + 5 µM compound **1g** (green symbols); 10 mM pNPP (black symbols), 10 mM pNPP (black symbols) + 5 µM compound **1g** (magenta symbols); 20 mM pNPP (purple symbols) and 20 mM pNPP + 5 µM compound **1g** (blu symbols). All reactions were initiated by adding enzyme (0.8 nM PTP1B) to the assay solution.

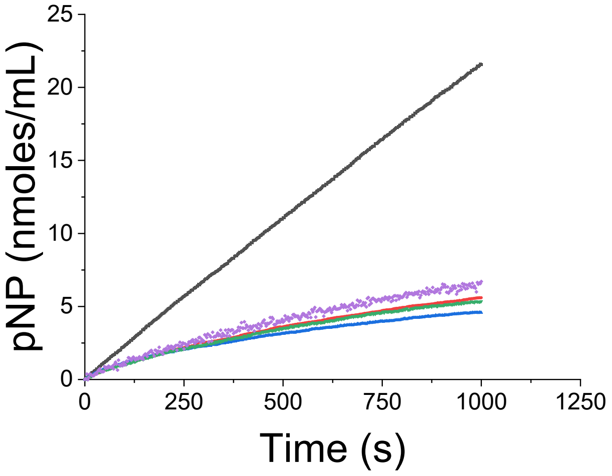

Figure S38. Continuous inhibition of PTP1B by compound **2f** in presence of increasing concentrations of substrate. The hydrolysis of substrate (pNPP) was monitored by measuring the release of p-nitrophenol (pNP) into the solution in the presence of a fixed concentration (10 µM) of compound **2f** and of 7.5 mM pNPP (blu symbols), 15 mM pNPP (green symbol), 20 mM pNPP (red symbols) or 40 mM pNPP (purple symbols). The hydrolysis rate obtained in the presence of 40 mM pNPP alone (without inhibitor) was described by the dark symbols. All reactions were initiated by adding enzyme (0.8 nM PTP1B) to the assay solution.

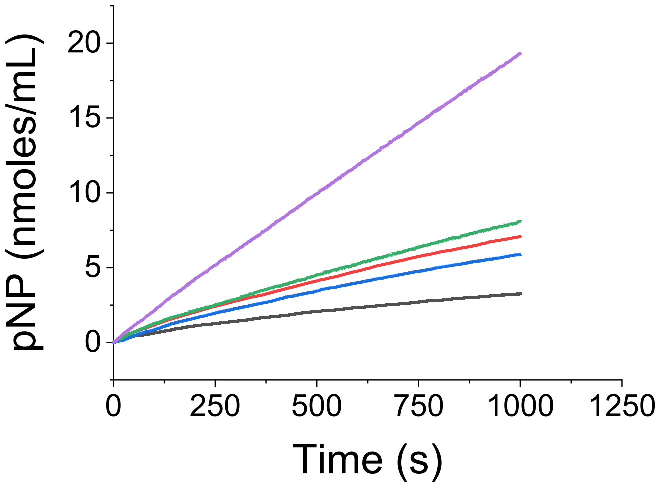

Figure S39. Continuous inhibition of PTP1B by compound **2g** in presence of increasing concentrations of substrate. The hydrolysis of substrate (pNPP) was monitored by measuring the release of p-nitrophenol (pNP) into the solution in the presence of a fixed concentration (5 µM) of compound **2g** and of 2.5 mM pNPP (black symbols), 5 mM pNPP (blu symbol), 10 mM pNPP (red symbols) or 20 mM pNPP (green symbols). The hydrolysis rate obtained in the presence of 20 mM pNPP alone (without inhibitor) was described by the purple symbols. All reactions were initiated by adding enzyme (0.8 nM PTP1B) to the assay solution.

Mechanism of action of compound 2e

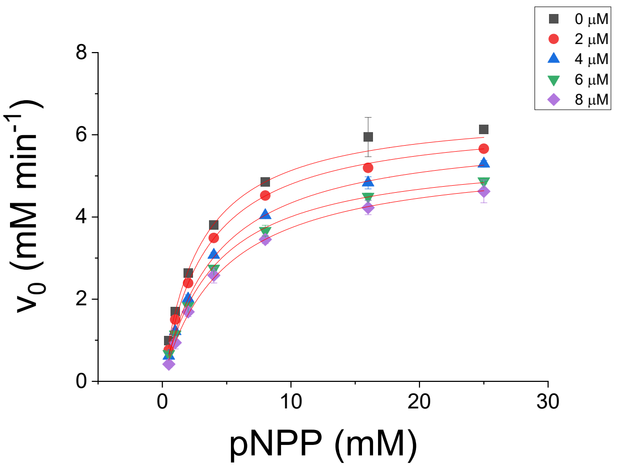

Figure S40. Dependence of Km and Vmax from the concentration of compound **2e**. To evaluate the impact of compound **2e** on both Km and Vmax, we determined the initial hydrolysis rate of pNPP at different substrate concentrations, and in the presence or not of increasing concentration of compound **2e**. The concentration of compound **2e** used were shown in the legend of the figure. The experimental data were fitted using the Michaelis-Menten equation. Each enzymatic assay was carried out in triplicate.

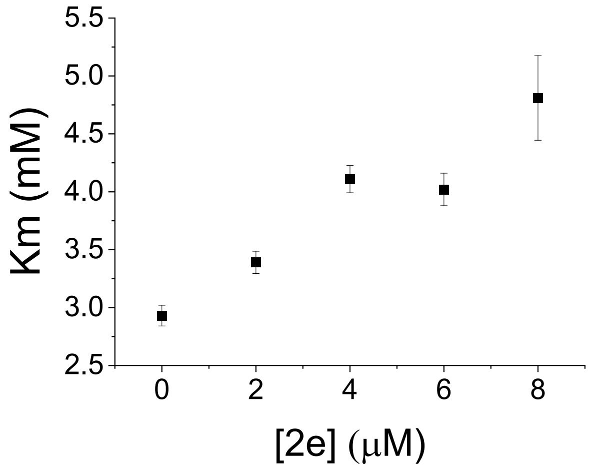

Figure S41. Dependence of Km from the concentration of compound **2e**. Data reported in the figure were obtained by fitting experimental data show in Figure 40 using the Michaelis-Menten equation

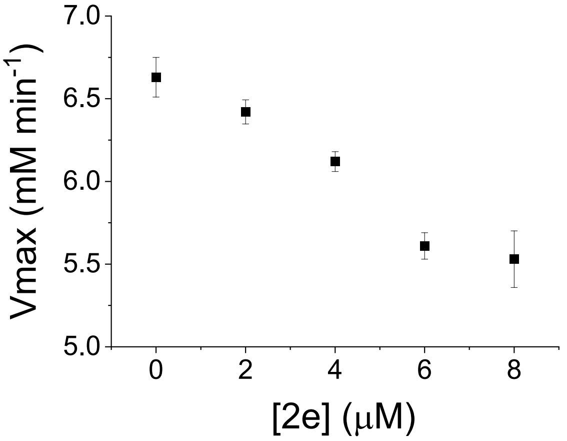

Figure S42. Dependence of Vmax from the concentration of compound **2e**. Data reported in the figure were obtained by fitting experimental data show in Figure 40 using the Michaelis-Menten equation

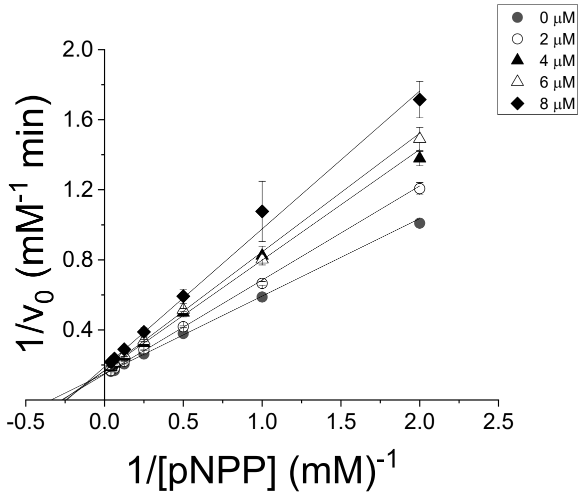

Figure S43. Lineweaver-Burk plot of compound **2e**. The experimental points reported in the figure were obtained manipulating the data reported in Figure S40. Data obtained were fitted using a linear fitting equation

Determination of Ki for compound 1g

Based on the data showed in Figures S34 and S37, we speculate that compound **1g** could behaves as a competitive slow-binding inhibitor (see Scheme S1),

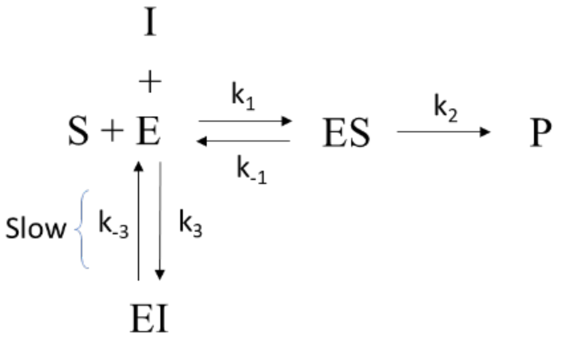

Scheme S1. Mechanism of action of compound **1g** (competitive slow-binding model).

where k_{-1}/k_1 represent the dissociation constant (Ks) of ES complex, and k_{-3}/k_3 ratio, the dissociation inhibitory constant (Ki) of EI complex.
To obtain more information, we fitted the experimental data reported in Figure 34 using the equation S1

$$[P]_t = v_s * t + \frac{(v_0 - v_s)(1 - e^{k_a t})}{k_a} \qquad \text{Eq. S1}$$

where $[P]_t$ is the concentration of the product of the reaction at time "t", v_0 and vs the initial and the steady state velocities, k_a the apparent first-order rate constant for development of steady state between free enzyme, inhibitor and EI complex in the presence of certain substrate and inhibitor concentrations. Then, we analysed the dependence of k_a from compound **1g** concentration (Figure S44).

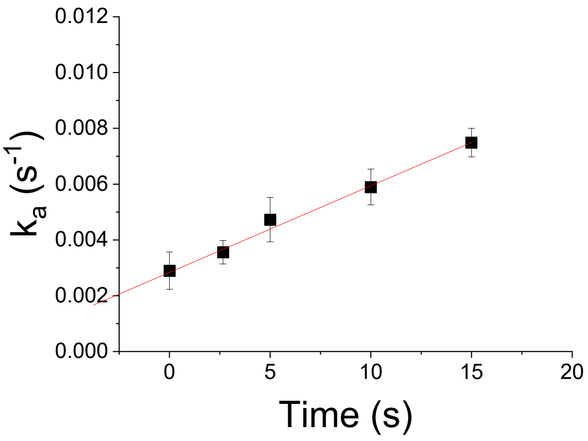

Figure S44. k_a secondary plot relative to compound **1g**. Data relative to the first-order rate constant for establishement of steady state were obtained fitting the data showed in Figure S34 with the equation S1.

Based on this model, the k_{-3} constant can be calculated as the intercept of the straight line with the "y" axis, whereas the k_3 can be determined by slope of the straight line, following the equation S2 [S1].

$$slope = \frac{k_3}{1 + \frac{[S]}{K_m}} \qquad \text{Eq. S2}$$

Therefore, the k_{-3}, k_3 and Ki resulted 0.00284 s^{-1}, 9,314*$10^{-4}$$\mu M^{-1}s^{-1}$, and 3.0 μM, respectively.

Determination of Ki for compound 2f

Data show in Figure S35 were fitted using equation S1 to determine both k_a and v_0 values at different concentrations of compound **2f**. Then we analysed the dependence of k_a, and v_0 from compound **2f** (Figure S45).

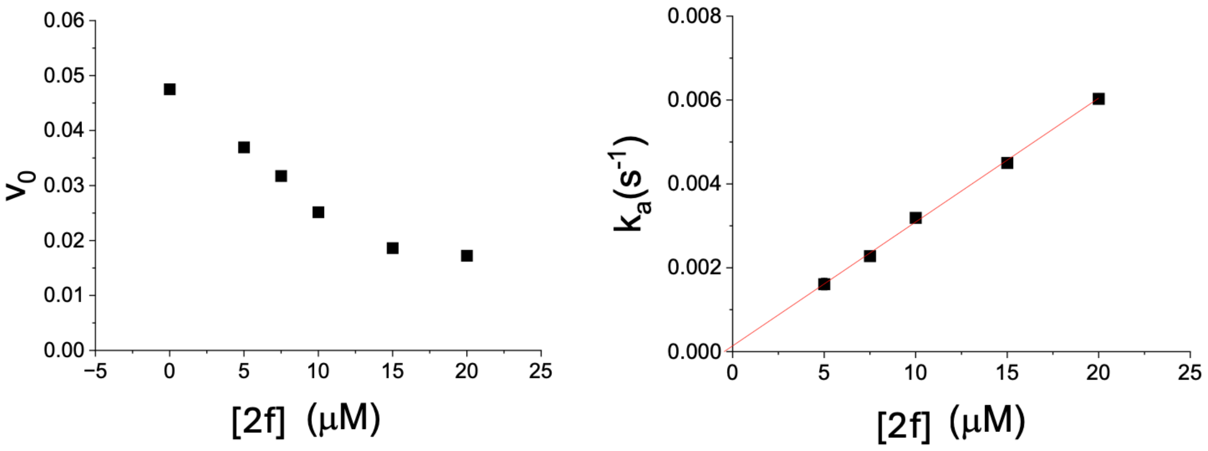

Figure S45. v_0 (left), and k_a secondary (right) plots relative to compound **2f**.

We observed that v_0 is hyperbolically dependent on [I], whereas k_a is a linear function of compound **2f**. This suggest that compound **2f** behaves as a slow-binding mixed type non-competitive inhibitor [S2] (see Scheme S2).

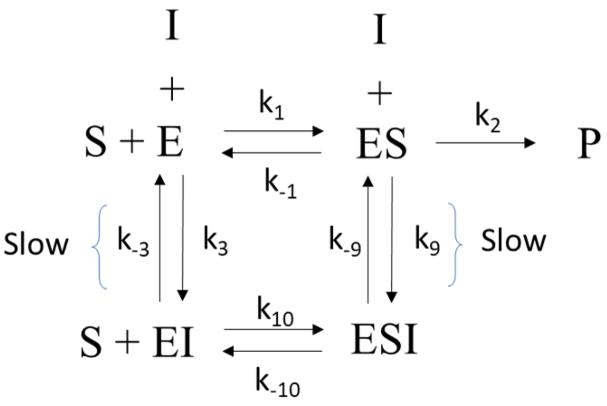

Scheme S2. Mechanism of action of compound **2f.**

In this scheme, k_{-1}/k_1 represent the dissociation constant (Ks) of ES complex, k_{-3}/k_3, the dissociation inhibitory constant (Ki) of EI complex, and k_{-9}/k_9, the dissociation inhibitory constant (αKi) of ESI ternary complex. Based on this model, the k_{-3} constant can be calculated as the intercept of the straight line showed in Figure S45B with the "y" axis, whereas the k_3 can be determined by slope of the straight line, following the equation S3 [S1].

$$slope = \frac{k_3}{1 + \frac{[S]}{K_m}} \qquad \text{Eq. S3}$$

Therefore, the k_{-3} resulted 1.39 $*10^{-4}$ s^{-1} and 8.9 10^{-4} $\mu M^{-1}s^{-1}$, respectively, while the Ki value, determined as the k_{-3}/k_3 ratio, resulted 0.16 µM. Moreover, based on this mechanism of action, the Ki' (αKi) should be calculated using the equation S4:

$$v_0 = \frac{V_{max}\frac{[S]}{K_m}}{1 + \frac{[I]}{K_i} + \frac{[S]}{K_m} * \left(1 + \frac{[I]}{\alpha K_i}\right)} \qquad \text{Eq. S4}$$

Fitting the data reported in Figure S45A with equation S4, the Ki' value resulted to be 6.9 µM.

Determination of Ki for compound 2g

Data show in Figures S36 and S39 suggested that compound **2g** behaves as a slow-binding non-competitive inhibitor (Scheme S2). Therefore, using equation S1, we calculated both v_0 and k_a values fitting the data reported in Figure S36.

Analysing the dependence of v_0 and k_a from the concentration of compound **2g**, we found that v_0 is hyperbolically dependent on [I], whereas the k_a values show a biphasic line behaviour (Figure S46), suggesting that the inhibitory activity of compound **2g** results from the interaction of more than one inhibitor molecule with the enzyme.

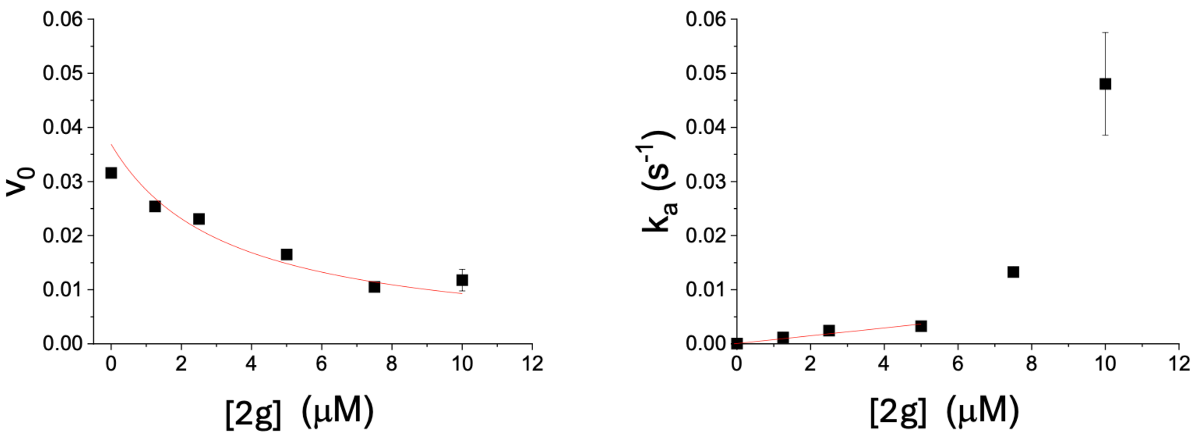

Figure S46: v_0 (left), and k_a secondary (right) plots relative to compound **2g**.

However, at low concentrations, the dependence of k_a on **2g** concentration appears to be linear and can be described by the model reported in Scheme S2. Therefore, fitting the first three k_a values with a straight line, we can calculate both the values of k_{-3}, k_3, and Ki referring to the site with greater affinity that resulted $5.8*10^{-5}$, $8.9*10^{-4}$ and 0.03 μM, respectively. Moreover, by using equation S4, we calculated a Ki' value of 2.2 μM.

References

S1. Masson, P; Lushchekina, S.V. Slow-binding inhibition of cholinesterases, pharmacological and toxicological relevance. *Arch. Biochem. Biophys.* **2016**, *593*, 60-68.

S2. Proceedings of the 5th international Beilstein symposium on experimental standard conditions of enzyme characterizations (pp.55-73), Publisher: Logos Verlag, Editors: M. G. Hicks, C. Kettner.

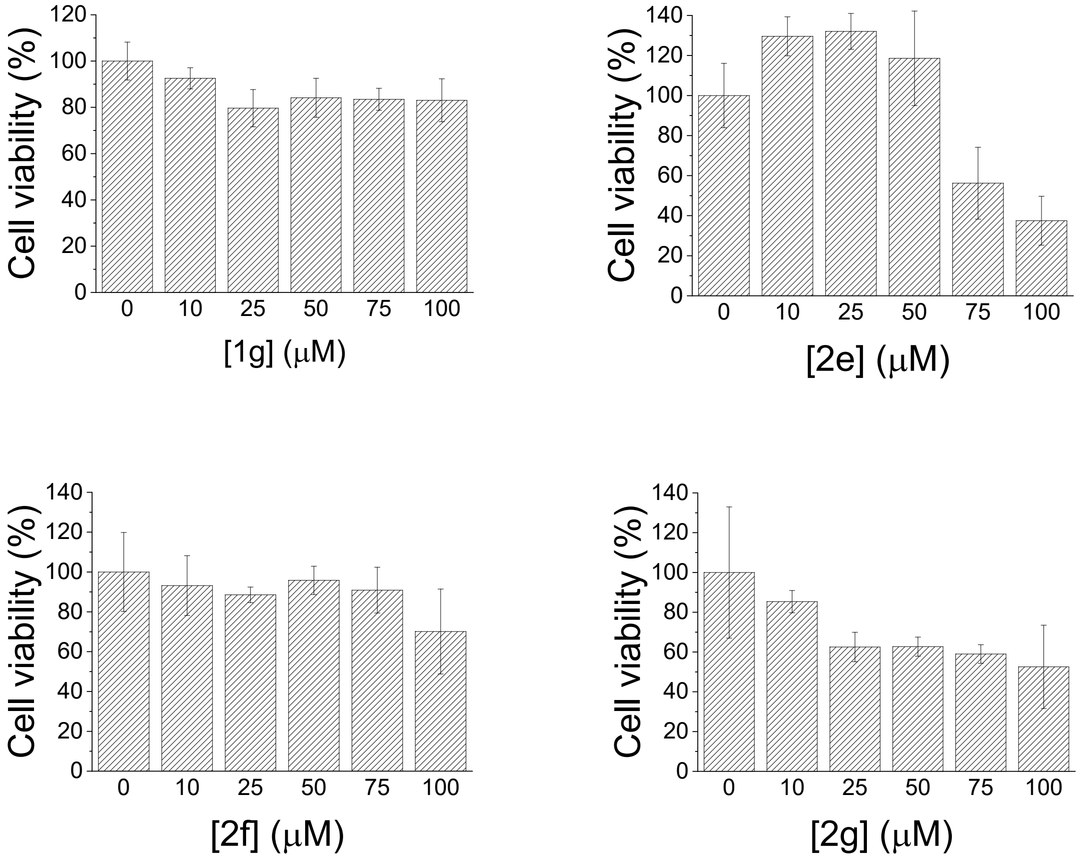

Figure S47. Toxicity assay of compound**s 1g** and **2e-g** on C2C12 cells. Murine muscle cells were seeded in 24-well plates and incubated at 37 °C. After 24 hours, the complete medium was replaced with fresh medium containing increasing concentrations of each compound, and the cells were incubated for an additional 24 hours at 37 °C. After this period, the medium was removed and replaced with fresh medium containing 0.5 mg/mL MTT reagent. Following a 1-hour incubation at 37 °C, the plates were collected, and the cells were washed with PBS to remove excess reagent. To determine cell viability, the cells were lysed using DMSO, which also dissolved the formazan crystals formed inside the cells. The absorbance of the resulting solutions was measured at 595 nm using a spectrophotometer. All data were normalized to the control assay. Each experiment was performed in quadruplicate. The data shown in the figure represent the mean ± SD.

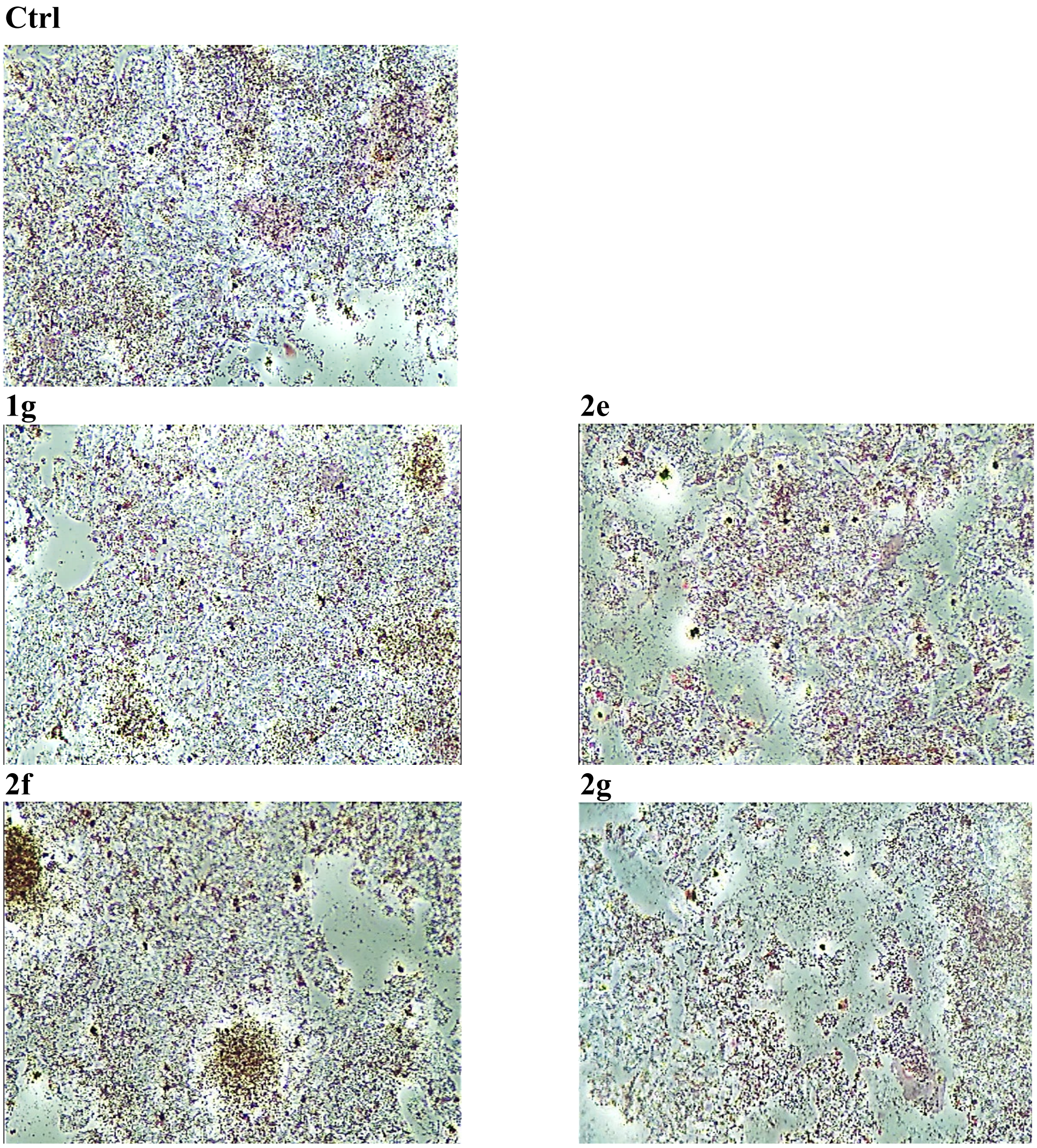

Figure S48. Representative images of liver cells stained with Oil Red O dye for the lipid accumulation assay. Liver cells grown in 24-well plates were treated with 0.4 mM oleic acid (OA) for 24 h and subsequently exposed to compounds 1g and 2e–g for an additional 72 h. Intracellular lipids were then visualized using Oil Red O staining. Before quantification, images of the liver cells were acquired using phase-contrast microscopy. The intracellular lipid content was determined by Oil Red O extraction and quantification using a spectrophotometer. The data obtained are reported in Figure 15 of the main text of the manuscript.

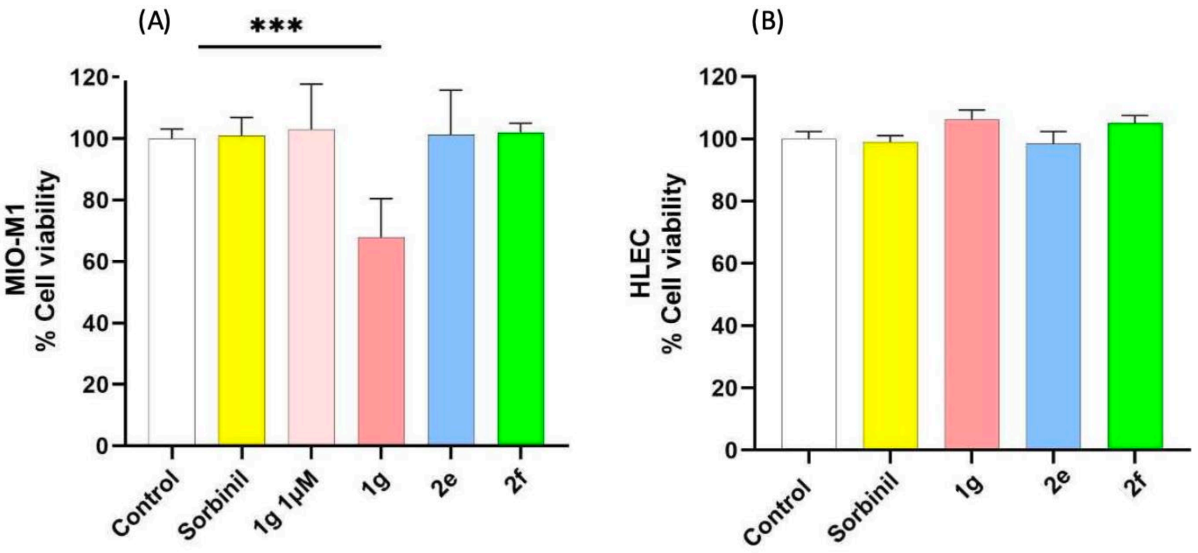

Figure S49. Effect of AKR1B1 inhibitors on cell viability. MIO-M1 (Panel A) and HLE cells were incubated at 37 °C for 48 h in proper media (see Methods) in the presence of 0.05% DMSO alone (control) or in the presence of the indicated compounds. If not otherwise stated, the final concentration of inhibitors was 5 µM. Cell viability was measured as described in Methods Section and is reported as % of the control value. Values are reported as the mean ± SEM of six independent measurements. Statistical analysis was performed using one-way Anova with Dunnet post hoc test. Significance was evaluated with respect to control cells. (***: $p<0.001$)
